# Reinforcement Learning in Patients With Mood and Anxiety Disorders vs Control Individuals: A Systematic Review and Meta-analysis

**DOI:** 10.1001/jamapsychiatry.2022.0051

**Published:** 2022-03-02

**Authors:** Alexandra C. Pike, Oliver J. Robinson

**Affiliations:** 1 Anxiety Lab, Neuroscience and Mental Health Group, Institute of Cognitive Neuroscience, University College London, London, United Kingdom; 2 Research Department of Clinical, Educational and Health Psychology, University College London, London, United Kingdom

## Abstract

**Importance:**

Computational psychiatry studies have investigated how reinforcement learning may be different in individuals with mood and anxiety disorders compared with control individuals, but results are inconsistent.

**Objective:**

To assess whether there are consistent differences in reinforcement-learning parameters between patients with depression or anxiety and control individuals.

**Data Sources:**

Web of Knowledge, PubMed, Embase, and Google Scholar searches were performed between November 15, 2019, and December 6, 2019, and repeated on December 3, 2020, and February 23, 2021, with keywords (*reinforcement learning*) AND (*computational* OR *model*) AND (*depression* OR *anxiety* OR *mood*).

**Study Selection:**

Studies were included if they fit reinforcement-learning models to human choice data from a cognitive task with rewards or punishments, had a case-control design including participants with mood and/or anxiety disorders and healthy control individuals, and included sufficient information about all parameters in the models.

**Data Extraction and Synthesis:**

Articles were assessed for inclusion according to MOOSE guidelines. Participant-level parameters were extracted from included articles, and a conventional meta-analysis was performed using a random-effects model. Subsequently, these parameters were used to simulate choice performance for each participant on benchmarking tasks in a simulation meta-analysis. Models were fitted, parameters were extracted using bayesian model averaging, and differences between patients and control individuals were examined. Overall effect sizes across analytic strategies were inspected.

**Main Outcomes and Measures:**

The primary outcomes were estimated reinforcement-learning parameters (learning rate, inverse temperature, reward learning rate, and punishment learning rate).

**Results:**

A total of 27 articles were included (3085 participants, 1242 of whom had depression and/or anxiety). In the conventional meta-analysis, patients showed lower inverse temperature than control individuals (standardized mean difference [SMD], −0.215; 95% CI, −0.354 to −0.077), although no parameters were common across all studies, limiting the ability to infer differences. In the simulation meta-analysis, patients showed greater punishment learning rates (SMD, 0.107; 95% CI, 0.107 to 0.108) and slightly lower reward learning rates (SMD, −0.021; 95% CI, −0.022 to −0.020) relative to control individuals. The simulation meta-analysis showed no meaningful difference in inverse temperature between patients and control individuals (SMD, 0.003; 95% CI, 0.002 to 0.004).

**Conclusions and Relevance:**

The simulation meta-analytic approach introduced in this article for inferring meta-group differences from heterogeneous computational psychiatry studies indicated elevated punishment learning rates in patients compared with control individuals. This difference may promote and uphold negative affective bias symptoms and hence constitute a potential mechanistic treatment target for mood and anxiety disorders.

## Key Points

**Question:**

Are there differences in reinforcement learning between patients with mood and anxiety disorders and control individuals?

**Findings:**

In this systematic review and meta-analysis, a novel computational simulation method showed differences in reinforcement learning between patients and control individuals. In particular, patients showed elevated punishment learning rates compared with control individuals.

**Meaning:**

These findings show that patients may be more likely than control individuals to modify their behavior in response to punishments, suggesting a possible mechanistic treatment target for negative affective bias symptoms.

## Teaser

This meta-analysis evaluates differences in reinforcement-learning behaviors between patients with depression and anxiety and control individuals.

## Introduction

Anxiety and depression are major individual and public health burdens.^1,2,3^ However, current treatment options have relatively low recovery rates (ranging from 28% to 52%^4,5,6^), and there are limited novel treatment prospects on the horizon. Part of the difficulty in developing and improving treatments is that we have an incomplete understanding of the mechanisms underlying anxiety and depression. However, a growing number of proposed mechanisms of mood and anxiety symptoms have emerged from a field known as computational psychiatry.^7,8,9,10,11^

The basic premise of computational psychiatry is that variations in how the brain performs computations (eg, in learning, perception, and decision-making) may, over time, generate emergent symptoms that are observed in various psychiatric disorders. For example, one theory is that individuals with a higher learning rate for negative stimuli (ie, punishment learning rate) might learn more from each negative event they experience, producing the negative affective bias that is frequently associated with depressive and anxious disorders.^12,13^ This theory situates the computational approach within clinical psychology concepts that date back to the 1960s^14^ and provides a mechanistic and falsifiable hypothesis for how clinical phenomena like negative affective bias may emerge.

The most common class of computational models tested in this field to date are reinforcement-learning models. Reinforcement learning can be defined as learning to obtain rewards and avoid punishments,^15^ and this type of computational model has some notable strengths. Namely, quantities computed by these models may be encoded in the phasic firing of dopamine neurons,^16^ providing a bridge between brain and behavior.^15^ Moreover, reinforcement-learning models can accurately mimic highly complex human behaviors.^17,18^ Further, there is a large body of evidence^19,20^ suggesting that those with depression and anxiety may show differences in processing rewards and/or punishments. Reinforcement-learning models may allow us to better understand this phenomenon.

We are now at the point where the body of case-control research investigating reinforcement-learning parameters in mood and anxiety disorders is sufficiently extensive that looking for overall patterns is possible. However, findings are varied. For example, different studies have argued that anxiety or depression may be associated with increased punishment learning rates^21,22,23^ or reduced reward sensitivity.^24^ While either of these differences would produce a negative bias toward the processing of punishments rather than rewards, the specifics can actually have considerable implications for how we treat such symptoms. For instance, reduced reward sensitivity in patients would require treatments that focused on how much the individual liked experiencing positive events, while treatments for elevated punishment learning rates would seek to encourage individuals to avoid immediately changing their behavior in response to negative outcomes.

The aim of this meta-analysis is therefore to assess consistencies across these reinforcement-learning studies and generate more highly powered estimates of the underlying group differences,^25^ hypothesizing that there will be a difference in reinforcement-learning parameters across groups. We first present the results of a conventional meta-analysis. However, this analysis was unsatisfactory for the modeling approaches used in computational psychiatry, as studies use both different tasks and different models to obtain their results.^26^ Is there a more principled way to combine the effect sizes from different tasks, models, and parameters?

To this end, a benefit of the modeling approach is that rather than simply taking a summary statistic over participants, individual-level trial-by-trial data are used to generate a proposed model of the underlying mechanisms. This generative model also provides precise predictions about how each individual’s behavior might generalize outside of the specific reported context. It is therefore possible to invert a reported model and simulate data for participants, even on tasks that they did not perform in the original study. As such, we can simulate performance for participants across studies on standardized benchmarking tasks, removing task inconsistencies across studies. We can then compare parameters across consistent models in our newly standardized data, removing model inconsistencies across studies. This method can increase the generalizability of these parameters, as we obtain model parameter estimates in this common space and use them to meta-analytically estimate parameter differences across groups. Thus, the aim of this article is to leverage the unique advantages of computational modeling to create a novel simulation-based meta-analytic method, which can be used to test the hypothesis that there are case-control differences in reinforcement learning across mood and anxiety disorders.

## Methods

**Figure 1.**
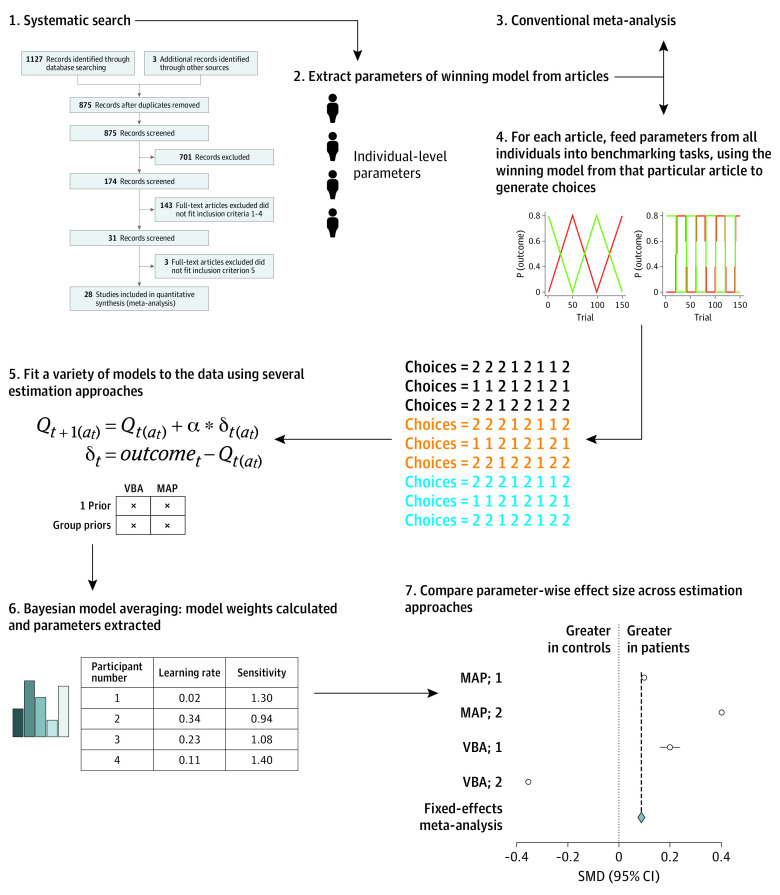
Study Procedure Note that the methods used in (5) were maximum a posteriori (MAP) with either 1 prior across all participants (MAP; 1) or separate priors for each group (MAP; 2); or variational bayesian analysis (VBA) with 1 prior (VBA; 1) or separate group priors (VBA; 2).

The procedure used in our meta-analysis is summarized below and in Figure 1, and explained in more detail in the eMethods in the Supplement.

### Article Selection and Extraction of Model Parameters

Articles were included if they met the following criteria:

1. Human participants.
2. Reported choice data from a cognitive task with monetary or point-based rewards or punishments.
3. Fit reinforcement-learning models to choice data.
4. Used a case-control design comparing control individuals with individuals with mood and/or anxiety disorders.
5. Reported sufficient statistics of all parameters in the models or made individual-level parameters available.

A standard systematic search was performed according to the Meta-analysis of Observational Studies in Epidemiology (MOOSE) reporting guideline (eAppendix 1 in the Supplement) between November 15, 2019, and December 6, 2019 (A.C.P.), and independently verified (O.J.R.). The search was repeated on December 3, 2020, and February 23, 2021. Further details may be found in the eMethods in the Supplement.

### Extraction of Winning Model Parameters From Articles

We used individual parameter estimates where available (eMethods in the Supplement) from the best-fitting model reported by the studies. Where these were not available in the article or a repository, we contacted the corresponding author to request them.

### Conventional Meta-analysis

We used a modified version of the Newcastle-Ottawa scale to assess study quality, with details and results reported in the eMethods in the Supplement. We performed a series of random-effects meta-analyses on raw values of the most commonly reported reinforcement-learning parameters from the included studies. Heterogeneity and publication bias were assessed and are reported in the eResults in the Supplement.

### Simulation Meta-analysis

In parallel with the conventional meta-analysis, we also performed a novel simulation meta-analysis. We describe this approach briefly here and in Figure 1 and in more detail in the eMethods in the Supplement.

In brief, we took the originally reported models from each article (eAppendix 2 in the Supplement) and used the model parameters reported for each participant to simulate choice behavior on 5 new benchmarking tasks. In other words, we used the generative models reported in the articles to anticipate the choices participants might have made if they had done the same 5 tasks without adjusting behavioral strategy (ensuring that all choices were in the same task space). We then fit a selection of reinforcement-learning models (overlapping with the models across all the original articles) to this new choice data set, and then extracted parameters using bayesian model averaging according to the strength of fit of each model to the data. This ensured all results were in the same model space. This enabled us to test the primary hypothesis: whether any parameters differed between groups.

There are a number of different methods of parameter estimation commonly used in computational psychiatry. To determine whether our findings were robust to analytic method, we used 4 different analytic strategies to estimate parameters. The 4 analytic strategies were combinations of maximum a posteriori and variational bayesian estimation and assumed either that all patients and control individuals came from the same underlying population (a single empirical prior was used for each parameter) or that they came from 2 separate underlying populations (2 priors were used, 1 for each group). We performed fixed-effects meta-analyses on the most common parameters for illustrative purposes to allow us to visualize consistency of effect sizes across approaches.

## Results

### Systematic Search

After a systematic search, 27 articles were included.^21,23,24,27,28,29,30,31,32,33,34,35,36,37,38,39,40,41,42,43,44,45,46,47,48,49,50^ The total number of participants included was 3085, 1242 of whom were patients with mood and anxiety disorders. A PRISMA diagram and a summary of the studies included, as well as an assessment of study quality, can be found in the eResults in the Supplement.

### Conventional Meta-analysis

**Figure 2.**
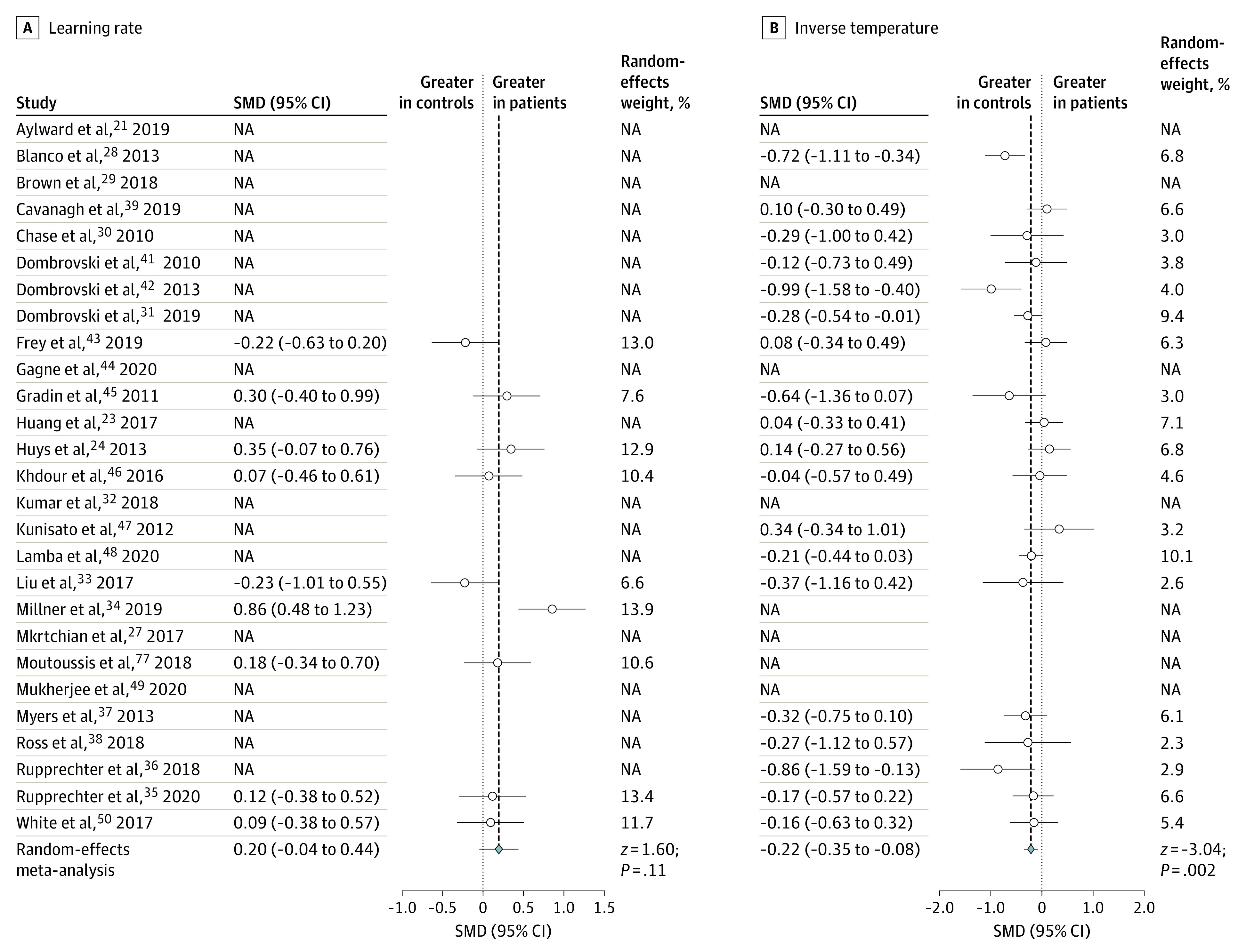
Forest Plots for the Conventional Meta-analysis Comparing Learning Rate and Inverse Temperature The figure shows standardized mean differences (Hedges *g*) between patients and control individuals. Where the relevant parameter was not included in the original article, the standardized mean difference is marked as not applicable (NA). SMD indicates standardized mean difference.

**Figure 3.**
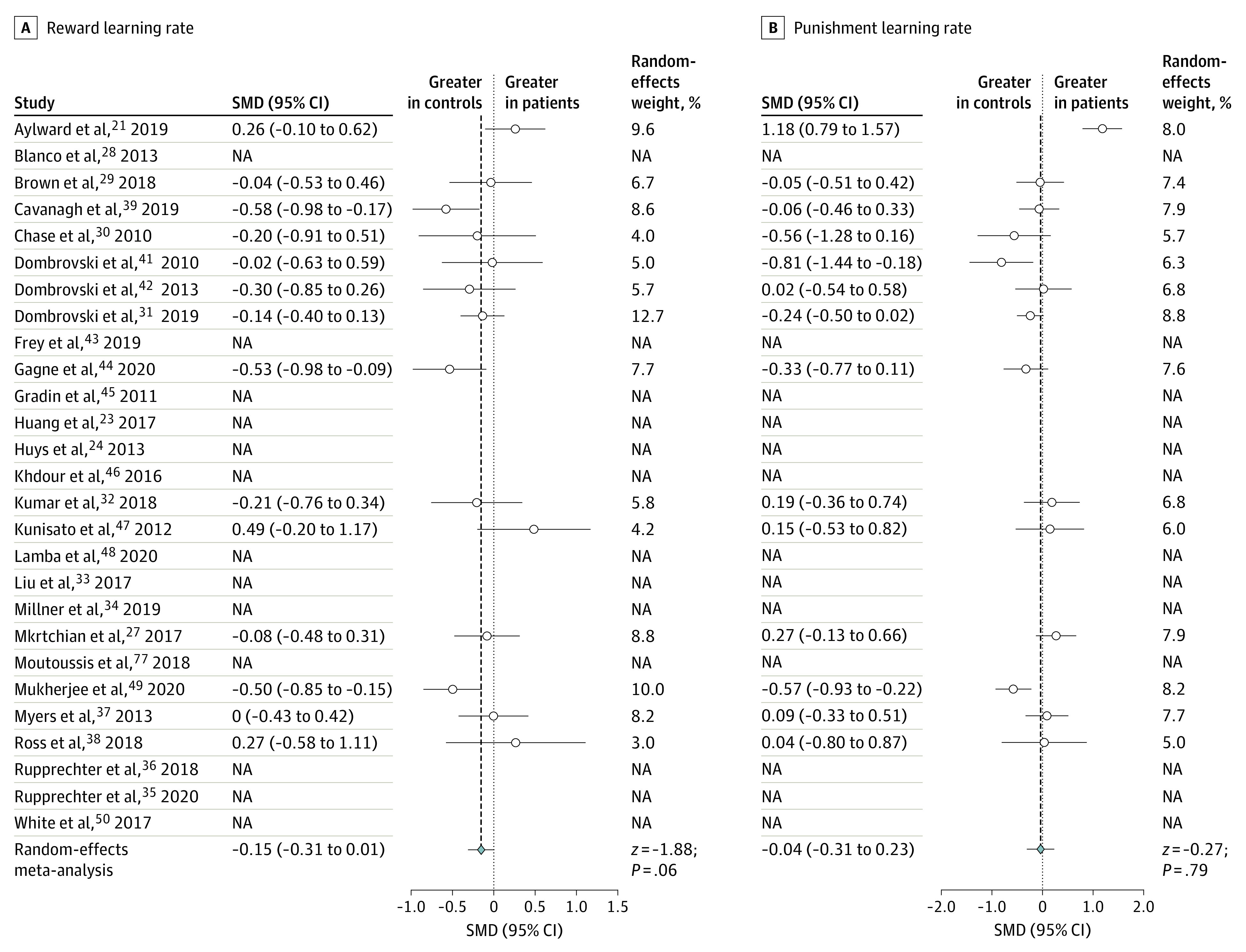
Forest Plots for the Conventional Meta-analysis Comparing Reward Learning Rate and Punishment Learning Rate The figure shows standardized mean differences (Hedges *g*) between patients and control individuals. Where the relevant parameter was not included in the original article, the standardized mean difference is marked as not applicable (NA). SMD indicates standardized mean difference.

After individual-level parameters had been extracted, we performed a series of random-effects meta-analyses to examine whether any of the most commonly reported parameters showed differences between patients and control individuals. There was no parameter that all articles had in common, highlighting the importance of our simulation approach for inference across all included studies. There was no significant standardized mean difference in a single learning rate parameter (9 of 27 articles reported this parameter: standardized mean difference, 0.196 [95% CI, −0.044 to 0.437]; Figure 2A). However, inverse temperature (or temperature, which we converted to inverse temperature) was elevated in control individuals (represented in 19 of 27 articles: standardized mean difference, −0.215 [95% CI, −0.354 to −0.077]; Figure 2B). Some articles reported learning rates that were separated by valence: there was no significant standardized mean difference in these parameters (reward learning rate was represented in 14 of 27 articles: standardized mean difference, −0.152 [95% CI, −0.310 to 0.006]; Figure 3A; punishment learning rate was also represented in 14 of 27 articles: standardized mean difference, −0.037 [95% CI, −0.306 to 0.232]; Figure 3B). There was evidence of moderate to substantial heterogeneity based on the values of the between-study variance of true effect sizes (τ^2^) and the approximate proportion of total variability (*I*^2^) (eResults in the Supplement).

### Simulation Meta-analysis

We used bayesian model averaging to obtain parameter estimates from each model in proportion to empirically determined model weights based on bayesian information criterion values. Subsequently, we performed 4 multivariate analyses of variance, 1 corresponding to each different parameter estimation method (dependent variables included all parameters for which there was at least 1 estimate), including group, study, and task as main effects. Each of these indicated that there was a main effect of group (Table), suggesting that there was a general difference in reinforcement learning between patients and control individuals regardless of estimation method.

**Table.** Results From the Multivariate Analysis of Variance^a^

Analysis method	Main association	Approximate *F*	*df*		*P* value
			Numerator	Denominator	
**VBA**					
1^b^	Group	971.30	16	15 273 788	<.001
	Task	60 255.67	64	61 095 164	<.001
	Study	14 795.04	416	244 380 848	<.001
2^c^	Group	10 590.50	16	15 273 788	<.001
	Task	47 039.51	64	61 095 164	<.001
	Study	11 683.04	416	244 380 848	<.001
**MAP**					
1^b^	Group	672.91	16	15 273 788	<.001
	Task	20 178.88	64	61 095 164	<.001
	Study	3636.98	416	244 380 848	<.001
2^c^	Group	1707.41	16	15 273 788	<.001
	Task	20 696.29	64	61 095 164	<.001
	Study	6560.50	416	244 380 848	<.001

Abbreviations: MAP, maximum a posteriori; VBA, variational bayesian analysis.

^a^ Full univariate results for all parameters can be found in the eResults in the Supplement.

^b^ Parameters were estimated using a hierarchical bayesian approach with only a single prior over groups.

^c^ Parameters were estimated using a different prior for each group.

There were also effects of study and task. We describe the effect of task further in the eResults in the Supplement. Briefly, recovery for separate reward and punishment learning rates was notably worse in benchmarking tasks in which rewards and punishments were nonindependent. In a supplementary analysis, we show that our findings held when only including the benchmarking tasks with good recovery. However, it is also possible that this issue is present in the raw parameter data that we used in this meta-analysis: not all tasks in the original article had orthogonal rewards and punishments.

**Figure 4.**
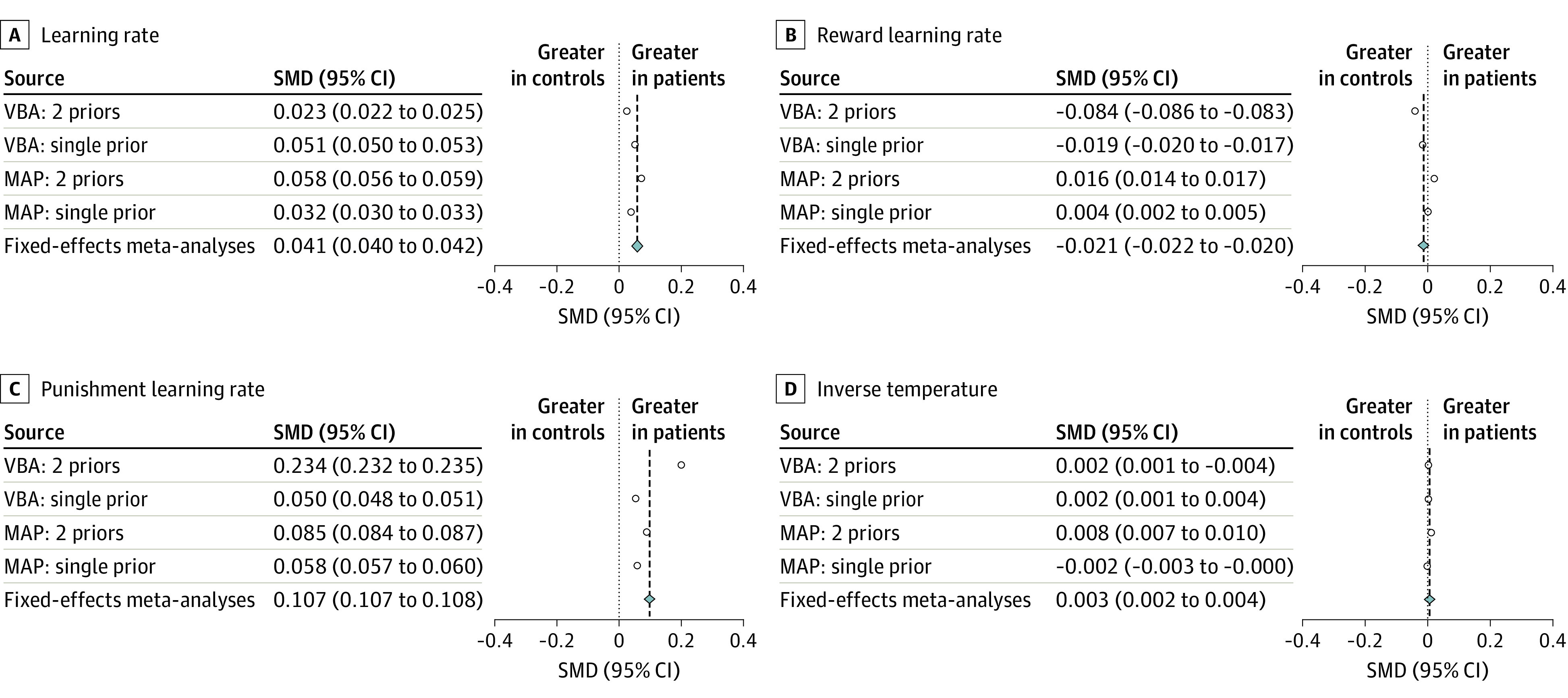
Forest Plots of the Cohen *d* Effect Sizes for the 4 Most Highly Represented Parameters From the Simulation Meta-analysis Fixed-effects meta-analyses were performed over the different analytic approaches. Each effect size represents a different analytic approach. Note that 95% CIs are not visible here as they overlap with the effect sizes. MAP indicates maximum a posteriori analysis; VBA, variational bayesian analysis.

We examined the effect of group for the parameters that were represented most frequently after bayesian model averaging. Statistics are shown in the eResults in the Supplement, and a summary of the effect sizes for each approach can be seen in Figure 4.

Here, we report the standardized mean differences for the 4 most highly represented parameters from our simulation meta-analysis using bayesian model averaging, combined using a fixed-effects meta-analysis. Across analysis methods (Figure 4), we saw a meaningful increase in punishment learning rates (standardized mean difference, 0.107 [95% CI, 0.107 to 0.108]) in patients vs control individuals and a slight decrease in reward learning rates (standardized mean difference, −0.021 [95% CI, −0.022 to −0.020]) with single learning rates also showing a slight increase (standardized mean difference, 0.041 [95% CI, 0.040 to 0.042]). Inverse temperature, which appeared to be different in a conventional meta-analysis, showed only a negligible difference (standardized mean difference, 0.003 [95% CI, 0.002 to 0.004]).

Subgroup analyses that investigate how these results varied by participant group, and meta-regressions controlling for study quality, year of publication, and parameter-level uncertainty, are in the eResults in the Supplement.

## Discussion

Our conventional meta-analysis suggested the only difference in reinforcement-learning parameters between patients and control individuals was in inverse temperature, with patients showing lower inverse temperature. However, the limitations of conventional methods when applied to computational modeling research were apparent: many articles did not have parameters in common. Using our novel meta-analytic method to estimate parameters for all articles across consistent task space and model space, we found meaningfully higher punishment learning rates and slightly lower reward learning rates in patients than in control individuals. This was seen alongside negligible group differences in inverse temperature.

The primary finding from our simulation meta-analysis was that those with mood and anxiety disorders showed a different balance between reward and punishment learning rates compared with control participants. Specifically, patients updated their learned values meaningfully more than control individuals after receiving a punishment and slightly less than control individuals after receiving a reward outcome. This association with learning rates was not apparent in our conventional meta-analysis; however, only half (14 of 27) of the original studies included the parameters (separate reward and punishment learning rates) that we required to test this using conventional methods. This highlights a key strength of our new simulation approach, as we were able to test for differences in these parameters across all studies.

The second key finding is that we did not observe any robust evidence of meaningful differences in inverse temperature or outcome sensitivity across patients and control individuals using our novel simulation method. It is worth bearing in mind that these parameters incorporate noisiness, participant exploration, and sensitivity to outcomes and thus are perhaps not pure estimations of either choice stochasticity or outcome sensitivity. This null finding is also made unclear by the poorer recovery of sensitivity effect sizes using our pipeline (eResults in the Supplement), and the fact that a larger association with inverse temperature was observed when controlling for study quality in a meta-regression. However, on the basis of our results, we tentatively suggest that how individuals learn and change their behavior to outcomes may be more important than other factors, such as how much individuals like or dislike outcomes. Further replication using tasks and models designed to robustly estimate these parameters will be necessary to confirm this interpretation, but it is interesting that our findings contrast with some accounts of depression and anxiety,^24,51^ which propose that depression is associated with reduced reward sensitivity (eg, anhedonia as a diagnostic criterion) and that anxiety is associated with increased punishment sensitivity (eg, biased attention or memory for threats).

Our findings may help refine our understanding of negative affective bias,^12^ in which patients focus on negative outcomes or occurrences. Specifically, our results allow us to tentatively distinguish between 2 potential causes of negative affective bias: greater subjective valuation of negative outcomes and different learning in response to negative outcomes.^13^ This meta-analysis found that patients with mood and anxiety disorders learned more from each instance of a negative outcome and showed no differences in how much they disliked these outcomes.

Furthermore, the results we have presented may be relevant to how clinicians target cognitive interventions. Rather than encouraging individuals with depression or anxiety to downweigh the subjective experience of negative outcomes or to sit with and tolerate resulting distress (as a necessary prerequisite for subsequently altering behavioral responses to distress, ie, in dialectical behavior therapy),^52^ interventions should focus directly on modifying how an individual changes their behavior in response to that negative outcome.^21^ For example, a therapist could try to encourage the individual to pause and not immediately change their behavior after something bad happens to provide the space to contextualize the negative outcome. This focus on punishment learning rates rather than punishment sensitivity could also help us understand how some common interventions work. For instance, flooding in exposure therapy (eg, where an agoraphobic individual is taken to a busy city center) may be effective through preventing the individual from performing their habitual behavioral responses to a negative outcome, rather than by tuning down their aversive response (ie, punishment sensitivity) to the exposure (eg, the crowd of people).

One of the strengths of reinforcement-learning models is that key quantities (ie, reward prediction errors) predicted by these models are thought to be reflected by neural activity.^16,53,54,55,56,57^ Notably, the learning rate may be an emergent property of neuromodulators, and in particular catecholamines, such as dopamine and noradrenaline.^58,59^ This has implications for drug interventions for depression and anxiety: if the balance of catecholamines modulates learning rates, pharmacological agents that affect learning rates may be of benefit to patients. Much previous work has focused on neuromodulators in depression and anxiety, following articulation of the monoamine hypothesis in the 1960s.^60,61,62^ Indeed, many of the first-line treatments for these disorders are selective serotonin reuptake inhibitors,^63,64^ which are associated with both serotonin and dopamine.^65^ However, many of these agents were discovered serendipitously, and the mechanisms by which they act on mood and anxiety symptoms are still unclear.^66^ As a result, there are few intermediate end points that have been validated for use in drug discovery. The results of this meta-analysis may point to a genuine intermediate end point: learning rates. In particular, individualized measures of learning rate balance could be obtained using straightforward behavioral tasks, thus allowing dose personalization and early indications of drug efficacy for individuals. This end point is also translationally valuable, as learning rates can also be measured in animals, potentially allowing preclinical drug discovery work.^67^ This meta-analysis therefore provides a possible first step toward connecting different levels of analysis in mental health research, from behavioral symptoms to the underlying neurobiology and pharmacology.

### Limitations

This study has several limitations. A core assumption of the field of computational psychiatry is that parameters and models generalize across tasks, samples, and model parameterizations (highlighted by the use of the same terms, such as learning rate, in different studies and models).^26^ We relied on this assumption for our conventional meta-analysis, although for our simulation meta-analysis we only required that parameters generalized across tasks and samples. Specifically, we assumed that it was possible to use a model that captured behavior on one task to simulate behavior on another. It is unlikely, however, that this is straightforwardly possible,^26^ which is reflected in the consistent effect of study we found on all parameter values. Particularly concerning is recent empirical evidence suggesting that parameters may not be stable within individuals either over time^68^ or across different tasks.^69^ In addition, parameters may not be stable even within a task; learning rates are known to adjust with the volatility of the environment,^13,70,71^ which also changes between tasks, along with other variables (eg, responses, timing, outcomes, and contingency structure). Furthermore, parameters defined for different tasks with different underlying statistics may play different roles in new tasks. Understanding how the parameters derived from one task relate to another—perhaps by establishing task-specific parameter norms or by testing the same individuals across multiple tasks—will allow stronger inferences about parameters to be drawn in this kind of meta-analytic approach. If parameters from one task do not relate at all to each other, this will be a serious issue for the use of reinforcement-learning models in computational psychiatry. An implicit assumption is that these parameters are representative of underlying generative processes across tasks and are thus more relevant to real-world behavior than summary statistics, such as mean accuracy. Perhaps a more realistic assumption is that parameters do generalize, but imperfectly, between tasks. This can be observed in related families of models, such as drift diffusion models, the parameters of which do mostly generalize across tasks,^72^ including in clinical populations.^73^ It is nevertheless promising that we were able to observe consistent parameter effect sizes across different analytic methods despite this substantial source of noise.

The results from our conventional meta-analysis differ from our findings using our novel simulation method. Our conventional meta-analysis found differences in inverse temperature, but not with learning rate, namely reduced inverse temperature in patients. Reduced inverse temperature may reflect noisy choice data, which encompasses strategies not based on reinforcement learning, exploratory behavior, or simply nonspecific difficulties in attention and concentration^8,74^ that are often observed clinically. Future work might therefore measure general executive function alongside reinforcement learning to disentangle the contribution of learning-specific associations from overall cognitive function.^75,76^ However, the conventional meta-analysis was also limited by the lack of commonality across parameters, reducing the amount of usable data, and by lack of generalizability across task space and model space, which was the motivation behind the development of our novel method. Future work will be needed to fully assess and compare the conventional method with our novel one (although see the eResults in the Supplement for simulations indicating the effect sizes from our novel meta-analysis are generally underestimates).

On a related note, it is possible that our findings were driven, at least in part, by our selection of benchmarking tasks. As illustrated in the eResults in the Supplement, recovery depends on the structure of the task. Future work might adopt additional benchmarking tasks to further probe the robustness of meta-analytic differences to task specification.

Additionally, in this novel method, we did not carry forward all the information we had about parameter-level uncertainty to our final inference. However, the results of a meta-regression using parameter-level uncertainty (eResults in the Supplement) showed that the effect of group on punishment learning rate was robust to this source of noise.

Moreover, there was considerable heterogeneity in the included studies. This may be driven by the different participant groups, tasks, and models included in these studies, but another important source of variance might be the variety of different methods used in parameter estimation. Parameters estimated in original studies may have been subject to hierarchical fitting or not, may be regularized or not, may have been constrained or not, and the original authors may or may not have tested parameter and model recovery and stability. This should not prohibit meta-analytic inference, but is an additional source of noise that should temper confidence in meta-analytic estimates.

## Conclusions

Overall, this study provides support for the hypothesis that reinforcement learning differs across patients with mood and anxiety disorders and control individuals. Specifically, we demonstrated elevated punishment learning rates and reduced reward learning rates in patients. We concluded that negative affective bias in mood and anxiety disorders may be driven by patients being too quick to update their behavior in response to negative outcomes. Moreover, by providing a formal computational account of this process, we were able to associate these symptoms with different levels of analysis (eg, neurobiological and pharmacological) and gain a mechanistic insight into how psychological therapy may work.

## Notes

Supplement. eMethods eResults eReferences eAppendix 1. MOOSE Checklist for Meta-analyses of Observational Studies eAppendix 2. Original models and modifications
