# Supplementary material for: Reinforcement Learning in Patients With Mood and Anxiety Disorders vs Control Individuals: A Systematic Review and Meta-analysis
Source: JAMA Psychiatry. 2022 Mar 2;79(4):313–22. doi: 10.1001/jamapsychiatry.2022.0051 (PMC8892374; doi:10.1001/jamapsychiatry.2022.0051)
Supplement: Supplement. — eMethods eResults eReferences eAppendix 1. MOOSE Checklist for Meta-analyses of Observational Studies eAppendix 2. Original models and modifications [file jamapsychiatry-e220051-s001.pdf]

Supplemental Online Content

Pike AC, Robinson OJ. Reinforcement learning in patients with mood and anxiety disorders vs control individuals: a systematic review and meta-analysis. *JAMA Psychiatry*. Published online March 2, 2022. doi:10.1001/jamapsychiatry.2022.0051

eMethods
eResults
eReferences
eAppendix 1. MOOSE Checklist for Meta-analyses of Observational Studies
eAppendix 2. Original models and modifications

This supplemental material has been provided by the authors to give readers additional information about their work.

© 2022 Pike AC et al. *JAMA Psychiatry.*

Supplementary Materials

Contents

Supplementary Methods and Results 3

1 Supplementary Methods 3

1.1 Search strategy/Paper Selection 3

1.2 Individual-Level Parameter Generation 4

1.3 Conventional Meta-Analysis 4

1.3.1 Assessment of study quality 4

1.3.2 Scoring for study quality 5

1.3.3 Assessment of heterogeneity 5

1.3.4 Assessment of publication bias/small study effects 5

1.4 Feed parameters from all participants into simulated tasks 5

1.4.1 Number of trials 7

1.5 Repeat process for all papers and concatenate all choices 8

1.6 Fit a variety of models to the data 8

1.7 Bayesian Model Averaging: Model Weights Calculated 12

1.8 Bayesian Model Averaging: Extract Weights For All Participants 12

1.9 Repeat For Other Tasks and Estimation Methods 13

1.9.1 Variational Bayes 13

1.9.2 Maximum A-Posteriori 13

1.9.3 Markov-Chain Monte-Carlo (Unused) 13

1.9.4 Number of Priors 13

1.10 Perform Omnibus Multivariate Inference and Parameter-Wise Inference 14

1.11 Robustness Meta-Analysis 14

2 Supplementary Results 14

2.1 Systematic Search Results 14

2.2 Assessment of Study Quality 20

2.3 Conventional Meta-Analysis: Heterogeneity and Publication Bias 21

© 2022 Pike AC et al. *JAMA Psychiatry.*

2.4 Effect Size Recovery 21
2.5 Full Univariate Results.. 22

© 2022 Pike AC et al. *JAMA Psychiatry.*

2.5.1 Variational Bayes, Single Prior 22
2.5.2 Variational Bayes, Group Priors 23
2.5.3 Maximum A-Posteriori, Single Prior 25
2.5.4 Maximum A-Posteriori, Group Priors 26
2.6 Sub-Group Analyses 27
2.6.1 Learning Rate 27
2.6.2 Reward learning rate 27
2.6.3 Punishment learning rate 27
2.6.4 Inverse temperature 27
2.7 Meta-Regressions 28
2.7.1 Study quality 28
2.7.2 Year of publication 28
2.7.3 Parameter uncertainty 29
2.8 Task Effects 29
2.9 Task Sensitivity Analysis 30
2.10 Winner-Takes-All Model Analyses 31
2.10.1 Methods 31
2.10.2 Variational Bayes, Single Prior 32
2.10.3 Variational Bayes, Separate Priors 36
2.10.4 Maximum A-Posteriori, Single Prior 39
2.10.5 Maximum A-Posteriori, Separate Priors 42
2.11 Recoverability analysis on generated tasks 45
3 Supplementary References 50

MOOSE Checklist for Meta-analyses of Observational Studies 55

Original models and modifications 57
1 Aylward *et al.*, *Nat Hum Behav*. 3, 1116–1123 (2019). 58
2 Blanco, Otto, Maddox, Beevers, Love, *Cognition*. 129, 563–568 (2013). 58
3 Brown *et al.*, *eLife*. 7, e30150 (2018). 59
4 Cavanagh, Bismark, Frank, Allen, *Comput Psychiatr*. 3, 1–17 (2019). 60
5 Chase et al., Psychological Medicine. 40, 433 (2010). 60
6 Dombrovski *et al.*, *Psychol. Med.* 45, 1413–1424 (2015). 61

© 2022 Pike AC et al. *JAMA Psychiatry.*

7 Dombrovski et al., American Journal of Psychiatry. 167, 699–707 (2010). 61
8 Dombrovski, Hallquist, Brown, Wilson, Szanto, *Biological Psychiatry*. 85, 506–516 (2019)........ 61
9 Dombrovski, Szanto, Clark, Reynolds, Siegle, *JAMA Psychiatry*. 70, 1020 (2013)........ 62
10 Frey, Frank, McCabe, *Psychol. Med.*, 1–8 (2019). 62
11 Gagne, Zika, Dayan, Bishop, *eLife*. 9, e61387 (2020). 63
12 Gradin *et al.*, *Brain*. 134, 1751–1764 (2011). 64
13 Huang, Thompson, Paulus, *Biological Psychiatry*. 82, 440–446 (2017). 65
14 Huys, Pizzagalli, Bogdan, Dayan, *Biology of Mood & Anxiety Disorders*. 3, 12 (2013). 65
15 Khdour *et al.*, *Front Integr Neurosci*. 10, 20 (2016)........ 66
16 Kumar *et al.*, *Neuropsychopharmacology*. 43, 1581–1588 (2018). 66
17 Kunisato et al., Journal of Behavior Therapy and Experimental Psychiatry. 43, 1088–1094 (2012). 67
18 Lamba, Frank, FeldmanHall, *Psychol Sci*. 31, 592–603 (2020). 67
19 Liu, Valton, Wang, Zhu, Roiser, *Social Cognitive and Affective Neuroscience*. 12, 1520–1533 (2017). 68
20 Millner *et al.*, *Journal of Abnormal Psychology*. 128, 106–118 (2019). 69
21 Mkrtchian, Aylward, Dayan, Roiser, Robinson, *Biological Psychiatry*. 82, 532–539 (2017). 69
22 Moutoussis *et al.*, *PLOS ONE*. 13, e0201451 (2018). 70
23 Mukherjee, Filipowicz, Vo, Sattherwaite, Kable, *Journal of Abnormal Psychology* (2020)........ 71
24 Myers *et al.*, *PLOS ONE*. 8, e72508 (2013). 71
25 Rupprechter *et al.*, *Brain*. 143, 1946–1956 (2020). 72
26 Rupprechter, Stankevicius, Huys, Steele, Seriès, *Sci Rep*. 8, 13798 (2018)........ 72
27 White *et al.*, *AJP*. 174, 110–117 (2017). 73

Supplementary Methods and Results

1 Supplementary Methods

We used the following procedure (see subsections below) to estimate meta-analytic effect sizes.

1.1 Search strategy/Paper Selection

Searches of the Web of Knowledge, PubMed, Embase and Google Scholar using a web browser were performed by ACP between the 15/11/2019 and the 6/12/2019. OJR cross-checked this search in September 2020. This search was repeated by ACP on the 3/12/2020 and the 23/02/2021. Our search

© 2022 Pike AC et al. *JAMA Psychiatry.*

strategy is identical to one that has been shown to give optimal retrieval[1], with the exception that we substituted PubMed for MEDLINE as it indexes the references found in MEDLINE references, plus additional ones. As in Bramer et al.[1], we screened only the first 200 Google Scholar references returned using the relevance ranking. We used the keywords: ("reinforcement learning") AND (computational OR model) AND (depression OR anxiety OR mood). Other papers were added to this search after discussions with co-authors. After screening all the abstracts returned, we reviewed the full text of the remaining papers, rejecting those did not meet the inclusion criteria highlighted in the main text. If papers met all inclusion criteria except the fifth (availability of sufficient statistics or individual-level parameters), we contacted the corresponding author by email. We also contacted corresponding authors seeking individual-level parameters if they reported only sufficient statistics. We did not attempt to infer these parameters from figures or graphs if they were not reported in-text, due to the inaccuracy this would likely introduce. Only one paper found in the initial search was not in English, but this did not report a clinical group so was excluded on this basis. Abstracts of unpublished work were not included; preprints were.

1.2 Individual-Level Parameter Generation

In the case of studies for which individual-level parameters from the winning model were not available, but sufficient statistics of the distribution were (n=1/27), we generated 'individual level' parameters for the same number of agents as there were participants in each group in the study, based on the statistics provided by the publication. Parameters that were irrelevant for the benchmarking tasks were set at the mean for each participant (e.g. 'go bias' in a task without no-go trials), or not included if the relevant equation was not necessary for generating choice data (e.g. the component of a model which generated reaction time estimates[2]).

1.3 Conventional Meta-Analysis

We performed random-effects meta-analyses for the four most commonly-reported parameters, using the 'meta' package in R[3].

1.3.1 Assessment of study quality

We used a modified version of the case-control version of the Newcastle-Ottaway Scale for Assessing the Quality of Nonrandomized Studies in Meta-Analyses[4,5].

Selection

1. Disorder Definition: Is the case definition adequate?
A) Cases were defined as MDD/GAD/PTSD/other specific diagnosis according to DSM or ICD criteria according to a validated assessment tool or by an experienced clinician.
B) Cases were defined as MDD/GAD/PTSD/other specific diagnosis according to DSM or ICD criteria but the method for assessing disorder status was not stated.
C) Cases were described as 'clinically depressed/anxious/similar' but no further description was given.
2. Disorder Generality: Was a General sample of cases tested?
A) A General sample of the specific disorder was tested.
B) Recruitment of cases was restricted to a specific sub-sample (e.g. veterans, suicidal ideation).
C) No description given.
3. HC Selection: Selection of Controls
A) Controls were selected from the same population as cases.

© 2022 Pike AC et al. *JAMA Psychiatry.*

B) Controls were not selected from the same population as cases.
C) No description.
4. HC Definition: Definition of Controls
A) HC were clearly defined as having no current or past psychopathology.
B) HC were clearly defined as having no current psychopathology.
C) Controls were not clearly defined as having no current or past psychopathology.

Comparability (Comparability of cases and controls on the basis of the design or analysis)
1. Does the study control for Age: Yes/No/Unclear/In supplementary analyses only
2. Does the study control for Gender: Yes/No/Unclear/In supplementary analyses only
3. Does the study control for IQ: Yes/No/Unclear/In sensitivity analyses only
4. **Does the study control for Personal or Household Income/Occupation**: Yes/No/Unclear/In supplementary analyses only

1.3.2 Scoring for study quality

In order to perform a meta-analysis in which items were scaled for quality, we created an approximately continuous variable which results from the sum of the items above. A was counted as 3 points, B as 2, and C as 1 in the selection criteria; Yes was counted as 3 points, Supplementary only as 2 points; and both No and Unclear as 1 point in the comparability criteria.

1.3.3 Assessment of heterogeneity

Heterogeneity was assessed using an estimate of the between-study variance of true effect sizes (τ^2) and the approximate proportion of total variability (I^2)[6].

1.3.4 Assessment of publication bias/small study effects

We investigated publication bias using Eggers' test, with the Pustejovsky-Rodgers standard error correction.

1.4 Feed parameters from all participants into simulated tasks

For this purpose, five different tasks were designed (representing tasks commonly encountered in the mood and anxiety disorder literature) and the parameters for each paper, in conjunction with the winning model from each paper (see Supplementary Section below entitled 'Original Models and Modifications'), were used to produce a set of simulated choices on these benchmarking tasks for each original participant (Supplementary Figure 1).

Notably, we designed some of the tasks to be both representative of the literature and to allow accurate parameter estimation. We included tasks in which reward and punishment were independent to optimize recovery of separate learning rates; but also included more typical tasks in which they are not, to avoid biasing our inference. In particular, recovery of separate reward and punishment learning rates and sensitivity parameters improves as these outcomes become less correlated. Task 2 was specifically designed to have no correlation, and task 4 has minimal correlation whilst retaining a reversal structure. Details on the correlations between reward and punishment for the four tasks can be found in Supplementary Table 1.

Many of the included papers used probabilistic tasks, often including some form of reversal (as in tasks 3 and 4[7–11]) or some form of multi-arm bandit (as in tasks 1 and 2[12–16]). We included a go/no-go task as

three of the papers are based around such a task, and thus it seemed conservative to ensure that this task was included. For greater detail on the tasks reported in the particular papers please see the Supplementary Results (Section 2.1 below) and the original manuscripts.

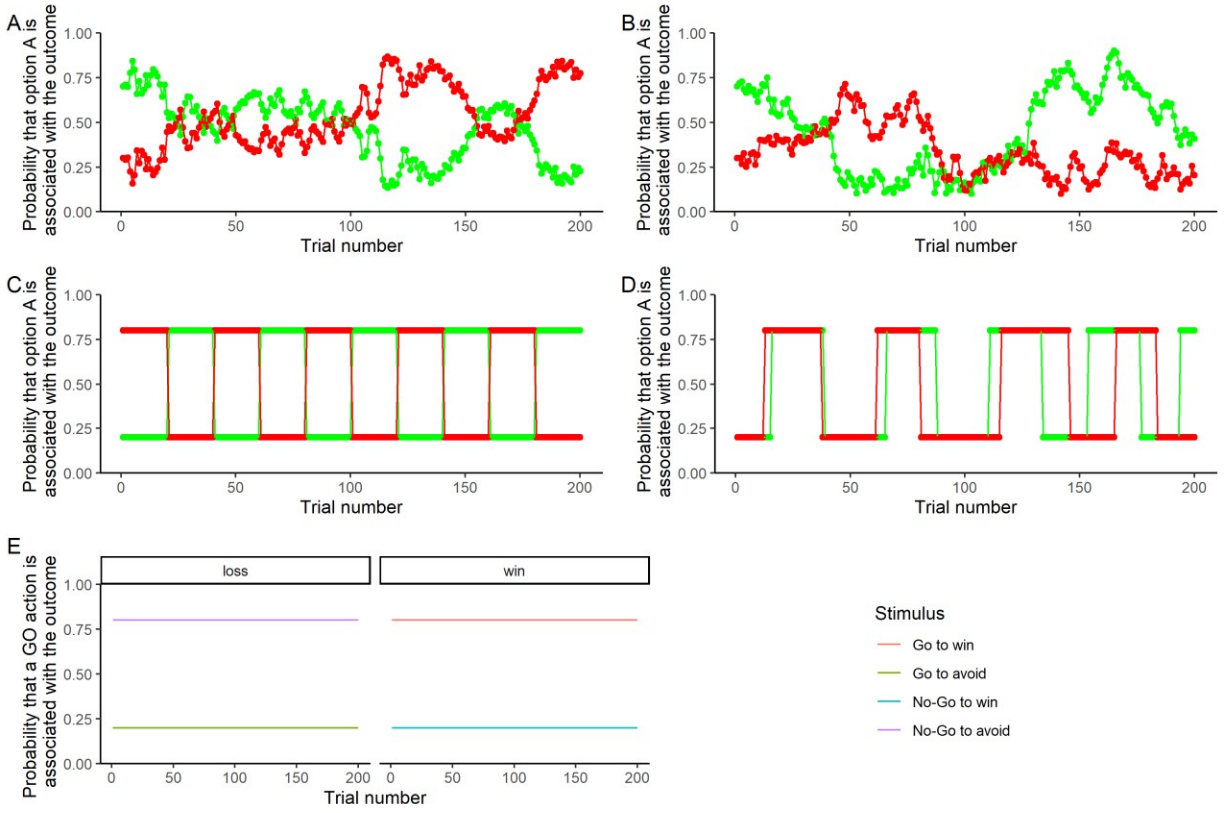

Supplementary Figure 1: The reinforcement structures of the different tasks used. A-D: Probability that one of the two stimuli is associated with reward (green line) or punishment (red line) for tasks 1 to 4 respectively. E) Probability that a `go' rather than a `no-go' response is associated with reward or punishment for task 5. A) The first task was a `restless' two-armed bandit task, with the probability of reward and punishment mirrored (reward probability = 1 - punishment probability) for a given option. Reward and punishment probabilities were therefore exactly inverted. The probability of reward for the first option was initialized at 0.7, and for the other option at 0.3. The probability of punishment for the first option was initialized at 0.3, and the probability of punishment for the second option at 0.7. These probabilities changed per trial by a Gaussian random walk, with mean 0 and standard deviation of 0.025. This random walk was bounded at probabilities of 0.9 and 0.1. B) The second task was identical to the first one, but separate reward and punishment random walks were generated. Therefore, the reward and punishment probabilities were independent of each other. C) The third task was a two-armed bandit with straightforward reversals - there were 10 reversals equally spaced within 200 trials, during which the probability of reward and probability of punishment alternated from 0.8 to 0.2. Reward and punishment were mirrored, and the probabilities of option 2 were the opposite of those for option 1. D) The fourth task was a two-armed bandit with straightforward reversals, which differs from the third task in that the timing of the reversals for reward and punishment were rounded values independently drawn from a normal distribution centered on the number of reversals divided by the number of trials, with a standard deviation of 5.E) The fifth task was a go/no-go task, in which four stimuli were presented. Each stimulus was associated with a different action-outcome pairing: `go to win' (in which selecting the stimulus led to a reward outcome, not selecting it led to a neutral outcome), `go to avoid losing' (selecting a stimulus leads to a neutral outcome, but not selecting leads to a punishment), `no-go to win' (not selecting a stimulus results in a reward, selecting it results in a neutral outcome), and `no-go to avoid losing' (not selecting a stimulus results in a neutral outcome, selecting it results in a punishment outcome). The probabilities of all these outcomes were kept constant: for `go to win' and `no-go to avoid losing' trials, the chance that the outcome (reward or punishment) would occur was 80%, for the other two stimuli it was 20%. Note that win and punishment trials are concurrent (not separated by blocks) and are presented separately for visualization purposes.

Task	Correlation	P value

© 2022 Pike AC et al. *JAMA Psychiatry.*

**1**	-1.000	<0.001
**2**	-0.034	0.634
**3**	-1.000	<0.001
**4**	-0.387	<0.001
**5**	NA	NA

Supplementary Table 1: Correlations between reward and punishment timecourses for tasks 1:5, and p values. All degrees of freedom were 198, as there were 200 trials. Task 5 did not have rewards and punishments presented on the same trials, so the correlation was 0.

1.4.1 Number of trials

We used variational inference to analyse parameter recovery for 500 participants on the most popular model (a model with one learning rate, one inverse temperature, one decay and one perseverance term; see Section 2.10 below) with different numbers of trials (20, 50, 100, 200, 300, 400, 500, 1000, 2000) on tasks of different lengths created using the same method as that used for the creation of task 2 above. We calculated the correlation between synthetic parameters, generated from realistic distributions, and also plot the 'confusion' for recovered parameters: i.e. the correlation coefficient between different parameters for the same participant (Supplementary Figure 2: Recovery *and confusion of parameter estimates using a model with one learning rate, one inverse temperature, one decay and one perseverance parameter with different numbers of trials, for two different task types. A) tasks generated using the same method as Task 1, B) tasks generated using the same method as Task 2. Dashed lines represent the mean for each type of correlation coefficient, shaded areas represent the standard error. Each point represents the correlation between two sets of parameters. Points in blue indicate the correlation coefficients between generated synthetic parameters and recovered parameters; values closer to 1 reflect a more accurate recovery process, with close correlations between synthetic and recovered parameters. Points in red indicate the correlation coefficients between different recovered parameters; values deviating from 0 reflect a large amount of trade-off between estimated parameters, which is typically due either to a poorly specified model or insufficient data, and values close to 0 reflect minimal trade-off. The vertical dotted lines indicate where the number of trials = 200, the number used in this paper. Notably, recovery does not improve significantly beyond 200 trials even for a task with weaker parameter recovery (task 1, panel C).*

High coefficients for recovery indicate that there is sufficient data for reliable estimation to occur, and low coefficients for confusion indicate that there is sufficient data that parameters are not trading off against each other. Notably, recovery seems to stabilize at around 200, and confusion is minimized at around this point. Recovery might be slightly improved by including greater numbers of trials, but this would incur a computational cost (i.e. the dataset to be fitted would be larger, requiring greater

© 2022 Pike AC et al. *JAMA Psychiatry.*

resources) that would not be justified by the increase in accuracy.

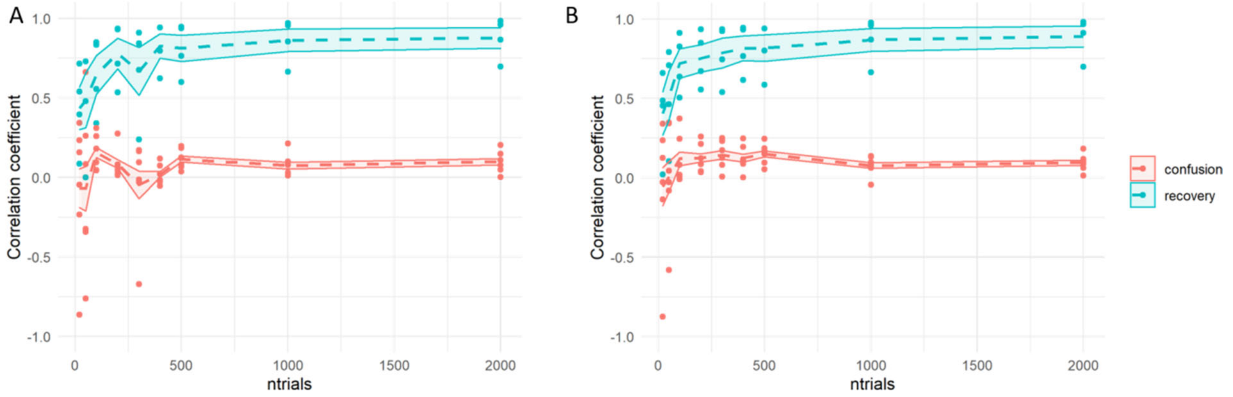

Supplementary Figure 2: Recovery and confusion of parameter estimates using a model with one learning rate, one inverse temperature, one decay and one perseverance parameter with different numbers of trials, for two different task types. A) tasks generated using the same method as Task 1, B) tasks generated using the same method as Task 2. Dashed lines represent the mean for each type of correlation coefficient, shaded areas represent the standard error. Each point represents the correlation between two sets of parameters. Points in blue indicate the correlation coefficients between generated synthetic parameters and recovered parameters; values closer to 1 reflect a more accurate recovery process, with close correlations between synthetic and recovered parameters. Points in red indicate the correlation coefficients between different recovered parameters; values deviating from 0 reflect a large amount of trade-off between estimated parameters, which is typically due either to a poorly specified model or insufficient data, and values close to 0 reflect minimal trade-off. The vertical dotted lines indicate where the number of trials = 200, the number used in this paper. Notably, recovery does not improve significantly beyond 200 trials even for a task with weaker parameter recovery (task 1, panel C).

1.5 Repeat process for all papers and concatenate all choices

Choice data were generated from all the agents from all the publications for all the simulated tasks. For each task, choice data generated based on different publications were concatenated together.

1.6 Fit a variety of models to the data

A variety of models were fit to the choice data. These models cover a wide model space, and overlap with the models from the original papers (see Supplementary Table 3 and the section below entitled 'Original Models and Modifications'). These models are standard reinforcement-learning models and are consistent with those described in the original papers. Models are summarized in Supplementary Table 2, and discussed more fully below.

Model	Learning rate	Sensitivity	Noise	Pavlovian Bias terms	Other terms
**1lr1b**	1 α		1 β		
**1lr2b**	1 α		2 β		
**2lr1b**	2 α		1 β		
**2lr2b**	2 α		2 β		
**1lr1s**	1 α	1 ρ			
**1lr2s**	1 α	2 ρ			
**2lr1s**	2 α	1 ρ			
**2lr2s**	2 α	2 ρ			
**1lr1s1lapse**	1 α	1 ρ	1 ξ		

**1lr2s1lapse**	1 α	2 ρ	1 ξ		
**2lr1s1lapse**	2 α	1 ρ	1 ξ		
**2lr2s1lapse**	2 α	2 ρ	1 ξ		
**1lr1s1lapse1bias***	1 α	1 ρ	1 ξ	1 go	
**1lr1s1lapse2bias***	1 α	1 ρ	1 ξ	1 go, 1 approach-avoid	
**1lr1s1lapse3bias***	1 α	1 ρ	1 ξ	1 go, 1 approach, 1 avoid	
**2lr2s1lapse1bias***	2 α	2 ρ	1 ξ	1 go	
**2lr2s1lapse2bias***	2 α	2 ρ	1 ξ	1 go, 1 approach-avoid	
**2lr2s1lapse3bias***	2 α	2 ρ	1 ξ	1 go, 1 approach, 1 avoid	
**1lr1b1d**	1 α		1 β		1 decay
**1lr1b1d1p**	1 α		1 β		1 decay, 1 perseveration
**2lr1b1d**	2 α		1 β		1 decay
**2lr1b1d1p**	2 α		1 β		1 decay, 1 perseveration

Supplementary Table 2: Models fit to generated choice data. Briefly, these models all included learning rates (either one for all trials, or two, where rewards and punishments were learnt about separately). Some also included sensitivity terms, which scale the reward or punishment either by the same value (if there is a single sensitivity term in that model) or by two different values (for rewards and punishments). Many of the models included either a temperature($\boldsymbol{\beta}$) or a lapse parameter($\boldsymbol{\xi}$), both of which parameterize decision-making noise, so there is not a perfect mapping between expected values of stimuli and the choices made on each trial. Six models (marked with an asterisk) were used for task 5 only as they incorporate biases for 'go' actions (i.e. the tendency of each participant to perform a 'go' response rather than a 'no-go' response) and biases towards approaching (go) and avoiding (no-go) rewarding and punishing stimuli respectively. We also used four models with a choice kernel, which is designed to capture participants' tendency to repeat their actions. These tasks and models are closely related to the majority of tasks and models used in the papers, which mostly included learning from probabilistic feedback, and generally included learning rates, sensitivity or inverse temperature parameters, and occasionally other task-specific parameters. More detail on the original tasks and best-fitting models can be seen in Supplementary Table 3.

All of the models contain at least one learning rate (α), which is bounded between 0 and 1, where a higher number reflects greater updating of values by the prediction error. Prediction error is defined as the difference between the expected and received value, as displayed in Supplementary Equation 1.

Supplementary Equation 1

$$\delta_t = outcome_t - predicted_t$$

We updated learnt values using Q-learning[17], in which the learning rate (α) acts as a multiplier on prediction error using the form shown in Supplementary Equation 2 for a given action (denoted as *'a'*):

Supplementary Equation 2

$$Q_{t+1(a_t)} = Q_{t(a_t)} + \alpha * \delta_{t(a_t)}$$
$$\delta_t = outcome_t - Q_{t(a_t)}$$

This update was only performed for stimuli which were chosen (i.e. no fictive updating took place for the unchosen stimulus).

© 2022 Pike AC et al. *JAMA Psychiatry.*

Some of the simpler models also have an inverse temperature parameter (β) which converts values into choice probabilities using a softmax. This parameter can range from 0 to infinity. As it tends to infinity, choices tend towards being identical with the higher value action on every try, but as it tends to zero, stochasticity/exploration increase, such that the agent does not choose the most highly-valued action on every trial (Supplementary Equation 3).

Supplementary Equation 3

$$P_{t(a_t)} = \frac{exp(Q_{t(a_t)} * \beta)}{\sum_{a=1}^{n} e\,xp(Q_{t(a_t)} * \beta)}$$

Some models have separate parameters for reward and punishment. Separate 'reward' and 'punishment' Q values are then calculated for each action, which are updated each trial regardless of whether a reward or punishment is actually received, and combined together. Notably, this is different to models in which a single Q-value is learnt for each action, and the learning rate used varies depending on either the valence of the prediction error or whether a reward or punishment was delivered – here, separate values for reward and punishment for each action are maintained. An example for a trial in which action '*a*' was chosen is shown in Supplementary Equation 4, where $reward_t$ indicates the reward outcome (could be 1 or 0), and $punishment_t$ the punishment outcome (could be -1 or 0).

Supplementary Equation 4

$$Q_{t+1(a_t),reward} = Q_{t(a_t),reward} + \alpha_{reward} * \delta_{t(a_t),reward}$$
$$Q_{t+1(a_t),punishment} = Q_{t(a_t),punishment} + \alpha_{punishment} * \delta_{t(a_t),punishment}$$
$$\delta_{t(a_t),reward} = reward_t - Q_{t(a_t),reward}$$
$$\delta_{t(a_t),punishment} = punishment_t - Q_{t(a_t),punishment}$$

$$Q_{t+1(a_t)} = Q_{t+1(a_t),reward} + Q_{t+1(a_t),punish}$$

If there were two separate temperature parameters for reward and punishment, the Q values for reward and punishment were then divided by them respectively before entry into the softmax equation. Otherwise, the Q values were summed together and then multipliedby β, as in Supplementary Equation 3.

Some more complex models also included sensitivity terms (ρ). These terms scale the value of the outcomes received to make them more or less valuable than they actually are, and typically range from 0 to infinity, with a higher sensitivity parameter indicating that outcomes are weighted more heavily (Supplementary Equation 5).

Supplementary Equation 5

$$Q_{t+1(a_t)} = Q_{t(a_t)} + \alpha * \delta_{t(a_t)}$$
$$\delta_{t(a_t)} = \rho * outcome_t - Q_{t(a_t)}$$

These sensitivity parameters can also be separate for reward and punishment, as is shown below in Supplementary Equation 6.

© 2022 Pike AC et al. *JAMA Psychiatry.*

Supplementary Equation 6

$$Q_{t+1(a_t),reward} = Q_{t(a_t),reward} + \alpha * \delta_{t(a_t),reward}$$
$$Q_{t+1(a_t),punishment} = Q_{t(a_t),punishment} + \alpha * \delta_{t(a_t),punishment}$$
$$\delta_{t(a_t),reward} = \rho_{reward} * win_t - Q_{t(a_t),reward}$$
$$\delta_{t(a_t),punishment} = \rho_{punishment} * loss_t - Q_{t(a_t),punishment}$$

It is not recommended to use both a sensitivity parameter and a temperature(β) parameter together as they are known to trade-off against each other[18]. In some models, therefore, we convert values to choice probabilities without using any temperature parameter, and in others, we introduce a lapse parameter (ξ), which also adjusts the shape of the softmax function but is considered to covary less with other model parameters (Supplementary Equation 7).

Supplementary Equation 7

$$P_{t(a_t)} = (1-\xi) * \frac{exp(Q_{t(a_t)})}{\sum_{a=1}^{n} e\,xp(Q_{t(a_t)})} + \frac{\xi}{2}$$

Some additional models are used solely for task 4 (see Section 2.4), as it is a go/no-go task, rather than a bandit. There are four possible states (omitted from earlier equations for simplicity, denoted henceforth as '*s*') in this task, each containing one stimulus, and participants make either a 'go' or a 'no-go' response. These include additional bias terms which have been found helpful to explain choice behaviour in this task (see e.g. Mkrtchian et al.[19]). One bias term that is used is an action-bias, which captures the tendency of participants to make a 'go' response rather than a 'no-go' response when presented with a stimulus (Supplementary Equation 8). This is an additive term to the value of the 'go' action. Lower-case '*q*' is the final value of the state/action pair, including the action-bias. Note that '*Q*' does not include the action-bias, and thus this bias is not included in value-updates (i.e. it is only used once, and not added again every time a 'go' action is chosen). Further, as shown in the last line of the equation, if *a* = 'no-go', actbias=0 (i.e. this bias only applies for 'go' responses).

Supplementary Equation 8

$$q_{t(a_t,s_t)} = Q_{t(a_t,s_t)} + actbias$$
$$Q_{t+1(a_t,s_t)} = Q_{t(a_t,s_t)} + \alpha * \delta_{t(a_t,s_t)}$$
$$\delta_{t(a_t,s_t)} = outcome_t - Q_{t(a_t,s_t)}$$
$$a = nogo \Longrightarrow actbias = 0$$

Other possible terms are Pavlovian bias terms. These are multiplied by the overall value of the state (not the state-action pair). If there is a single Pavlovian bias term, this is used regardless of what the state value is. Here, again, the biases are only applied if the action is 'go' (Supplementary Equation 9).

© 2022 Pike AC et al. *JAMA Psychiatry.*

Supplementary Equation 9

$$q_{t(a_t,s_t)} = Q_{t(a_t,s_t)} + actbias + pavbias * value_{t(s_t)}$$
$$Q_{t(a_t,s_t)} = Q_{t(a_t,s_t)} + \alpha * \delta_{t(a_t,s_t)}$$
$$value_{t+1(s_t)} = value_{t(s_t)} + \alpha * (outcome_t - value_{t(s_t)})$$
$$\delta_{t(a_t,s_t)} = outcome_t - Q_{t(a_t,s_t)}$$
$$a = nogo \Longrightarrow actbias = 0$$
$$a = nogo \Longrightarrow pavbias = 0$$

Task 4 is designed such that the 'go-to-win' and 'no-go-to-win' stimuli are only associated with reward outcomes or no outcomes, whereas 'go-to-avoid' and 'no-go-to-avoid' stimuli are only associated with loss outcomes or no outcomes. This means that if two Pavlovian bias terms are used, therefore, the 'approach' bias is used only for the states associated with the 'go-to-win' and 'no-go-to-win' stimuli, and the 'avoid' bias is used for the 'go-to-avoid' and 'no-go-to-avoid' stimulus states. The equations therefore look much as in Supplementary Equation 9, except that the *pavbias* becomes *approachbias* in the two states associated with rewards, and *avoidbias* in the other two states. As previously, other parameters (i.e. learning rates and sensitivity terms) can also be separated for reward and punishment. If this is the case, reward and punishment Q values are updated separately as in Supplementary Equation 4 or Supplementary Equation 6, then combined before being passed into the softmax function.

We also incorporated a choice kernel into some of our models, as this may capture participants' tendencies to repeat their previous actions[20,21]. This type of model also converts learnt values into choice probabilities. Here, a choice trace is introduced to the softmax, as in Supplementary Equation 10, and in some models is weighted by a perseverance parameter (otherwise, this weight is just set to 1, in models using a choice kernel but with no perseverance). High values of the perseverance parameter reflect a large influence of previous choices on the current choice. This choice kernel is updated on each trial as in Supplementary Equation 11, such that if the action is chosen, the choice trace is updated with the difference between 1 and the trace, scaled by a 'decay' parameter, and if the action is unchosen, the choice trace is updated based on the difference between 0 and the trace, also scaled by the 'decay' parameter.

Supplementary Equation 10

$$P_{t(a_t)} = \frac{exp(Q_{t(a_t)} * \beta - \varphi * C_{t(a_t)})}{\sum_{a=1}^{n} exp(Q_{t(a_t)} * \beta - \varphi * C_{t(a_t)})}$$

Supplementary Equation 11

$$where\ (a_t) is\ chosen \rightarrow C_{t+1(a_t)} = C_{t(a_t)} + decay * (1 - C_{t(a_t)})$$

$$where\ (a_t) is\ unchosen \rightarrow C_{t+1(a_t)} = C_{t(a_t)} + decay * (0 - C_{t(a_t)})$$

1.7 Bayesian Model Averaging: Model Weights Calculated

Bayesian Model Averaging (BMA) is a form of model selection that allows uncertainty over the winning model to be taken into account. We used Bayesian Model Averaging (BMA) to extract parameters from each model in proportion to how well it fit the data: a method which incorporates uncertainty as to the

© 2022 Pike AC et al. *JAMA Psychiatry.*

best-fitting model, rather than using a winner-takes-all approach (although we also report results using this approach, see Supplementary Section 2.8 below). We calculated approximate model weights using BIC values separately for each analytic strategy, and converted them by subtracting each BIC value from the maximum BIC value, and dividing by the sum to ensure they added to 1.

We also performed the variational Bayes analyses using two other weighting methods: the LOO package's[22] pseudo-BMA weights, and stacking[23] weights, to examine the generalizability of our results. The resulting multivariate ANOVAs were still highly significant (all *p* values <0.001), indicating that there is a substantial difference in reinforcement-learning parameters despite the precise method of model weighting chosen.

1.8 Bayesian Model Averaging: Extract Weights For All Participants

Subsequently, we drew parameters from the posterior distribution of that model with a frequency corresponding to the BMA model weights. Note that as maximum a posteriori estimation gives a single point estimate for each participant, our multiple draws of parameters in Bayesian Model Averaging were repeated instances of the same value, whereas the multiple draws from models fit using variational Bayes are equivalent to weighted samples from the approximate posterior.

The posterior means of all possible parameters in for all tasks were analysed together, in a 2(Group: patients, controls) x 5 (Task) multivariate ANOVA. Values which were 'NA' (i.e. that parameter wasn't present in that model) were all imputed as 1s, though note that replacing these with any other number did not change the result. Note that, to convert BIC scores into weights, we subtracted all BIC values from the maximum BIC value, and then divided the output of this calculation by the sum of the calculation, resulting in weights that summed to 1.

1.9 Repeat For Other Tasks and Estimation Methods

Analytic degrees of freedom are frequently encountered in fields such as neuroimaging, and are also prevalent in computational modelling. Ergo, we use four common approaches in order to demonstrate the robustness of our findings to analytic choices. We also highlight why we did not choose to use some other possible approaches.

Specifically, we used hierarchical Bayesian variational inference[24] and maximum a-posteriori estimation, and also used either a single overall prior for each parameter, or a prior that was calculated separately for each group (patients vs. controls). Note that our generally preferred approach, which yields the most reliable estimates, Markov-chain Monte-Carlo sampling, proved impractical for a sample of this size.

1.9.1 Variational Bayes

We estimated the posterior distributions of parameters using variational inference, implemented in R (v4.0.2) and RStan (v2.19.3). We chose to use variational inference rather than Markov-Chain Monte-Carlo (MCMC) sampling, as variational Bayes produces estimates much more rapidly (especially important when estimating parameters for a large sample size, as in this case where MCMC sampling turned out to be computationally impractical) with limited loss of accuracy[25]. For parameters bounded between 0 and 1 (learning rates, lapse parameters), we used non-centered parameterizations, transformed using the Phi_approx function in Stan, with the location parameter drawn from a $Normal(0,3)$ distribution, and the scale parameter drawn from a $Cauchy(0,5)$ distribution. We

assumed $Gamma$ distributions for parameters bounded between 0 and +∞ (sensitivity parameters, inverse temperature parameters), with shape and inverse scale parameters drawn from a $Normal(1,5)$ distribution. For unbounded parameters, we again used non-centered parameterizations, with the location parameter drawn from a $Normal(0,3)$ distribution, and the scale parameter drawn from a $Cauchy(0,5)$ distribution.

1.9.2 Maximum A-Posteriori

We also used a maximum a-posteriori approach. First, we ran maximum likelihood estimation, using fmincon (in MATLAB 2018b), which performs gradient descent to find the parameter combination that maximizes the likelihood of the sequence of choices made by each participant. To attempt to counteract the problem of localized solutions, we used 10 random starting points, and selected the parameters that produced the highest likelihood from these. Subsequently, we empirically estimated the distribution of the priors using betafit (for parameters bounded between 0 and 1), normfit (for bias parameters only) or gamfit (for parameters bounded between 0 and +∞), and then ran fmincon again (also with 10 starting points), and added the log likelihood for each parameter to the log probability mass function of the prior for that parameter value.

1.9.3 Markov-Chain Monte-Carlo (Unused)

The time taken to run a single model for a single group with MCMC ranged from 2-14 days using a computer with four cores, so the analysis for the whole set of five tasks and 12-18 models, if performed using MCMC, would conservatively take over 100 days. Therefore, we chose not to use this approach.

1.9.4 Number of Priors

It has been suggested that estimating separate priors for each group may lead to false positives[26], if priors with different means artificially inflate the differences between groups. However, using a single prior may artificially reduce the difference between group means, resulting in false negatives. It has been recently shown that the error in estimating effect size is greater when using a single prior than when using separate group priors, and therefore it may be more robust to run estimations using separate priors[25]. However, as both approaches are still common and have limitations, we use both in this paper. Therefore, for both variational Bayes and maximum a-posteriori approaches, priors are either specified as applying to all participants (regardless of group), or are separated by group.

1.10 Perform Omnibus Multivariate Inference and Parameter-Wise Inference

Subsequently, we performed four multivariate analyses (corresponding to the four different analytic choices mentioned above: one for each combination of number of priors and estimation method).

These multivariate analyses aimed to test the hypothesis that there is a difference in reinforcement-learning parameter between patients and controls. We included all parameters as dependent variables if there was at least one instance of that parameter after parameter extraction using Bayesian Model Averaging. Factors were Group (2 levels: patients, controls), Study (27 levels) and the within-subject factor of Simulated Task (5 levels).

We then further investigated parameter-specific effects by examining the effect of group for the five parameters that were most strongly represented in the Bayesian Model Averaging analysis (i.e. the most frequently included parameters). For the full univariate results, see Section 2.5 below.

© 2022 Pike AC et al. *JAMA Psychiatry.*

1.11 Robustness Meta-Analysis

After performing the four multivariate analyses (corresponding to analytic strategy) noted above, we probed how robust the parameter effects were to these differences in analysis specification. We plotted the effect sizes resulting from the univariate analyses of two broad parameter classes: learning rates, and sensitivity parameters, generating illustrative fixed-effects meta-analytic estimates to draw overall conclusions[3]. We also fractionated these effects into diagnostic sub-groups (depression, anxiety, PTSD and mixed anxiety/depression) see Supplementary Section 2.6 below.

2 Supplementary Results

2.1 Systematic Search Results

The systematic search of databases returned 1127 results, and we added 3 extra papers that were identified as missing by the authors. We screened all resulting abstracts. After this screen, we reviewed the full text of the remaining papers, rejecting papers that did not meet the inclusion criteria above. If papers met all inclusion criteria except 5 (reporting of parameters), we contacted the corresponding author via email. We also contacted the corresponding author of papers which reported sufficient statistics of parameters (e.g. mean and standard deviation), but not individual-level parameters. 18 sets of individual-level data were sent to us as a result. Notably, there were 3 papers that met all the inclusion criteria but did not report either sufficient statistics or individual-level values of all the parameters in the model used, and either responded saying they were unable to send us the data (n=2) or did not respond after two email enquiries (n=1). These papers are noted below. Subsequently, 28 papers were included: two studies reported fully overlapping data, resulting in a set of 27 studies, detailed below in Supplementary Table 3. More details about the original models used can be seen in the below section entitled ‘Original Models and Modifications’. The whole search process is displayed in the PRISMA diagram (Supplementary Figure 3).

© 2022 Pike AC et al. *JAMA Psychiatry.*

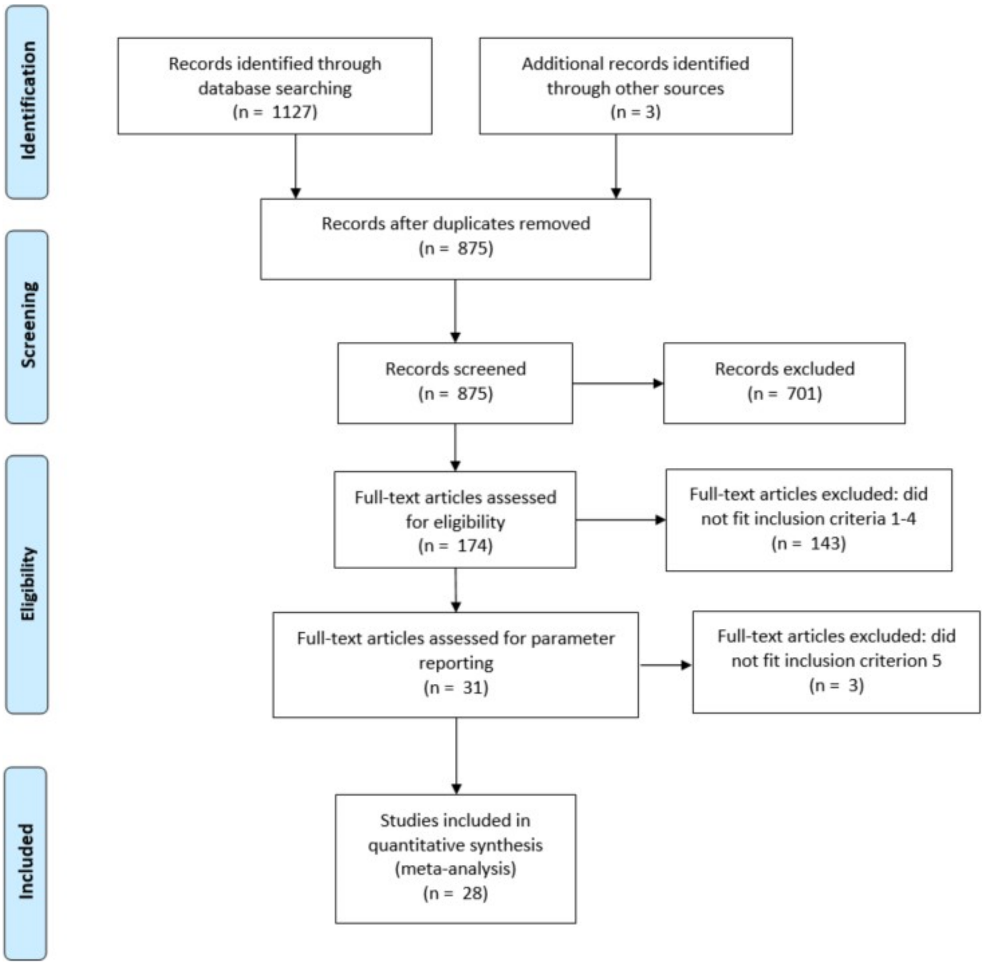

Supplementary Figure 3: PRISMA flow diagram[27] for papers included in this study.

© 2022 Pike AC et al. *JAMA Psychiatry.*

Authors and year	Total n	Patient n	Clinical Group	Task	Learning rates	Lapse/ temperature	Sensitivity	Other	Notes
Aylward et al. 2019[16]	132	44	Mood/ anxiety disorders	Four-armed bandit	2 (reward and punishment)	Lapse	Decay		
Blanco et al. 2013[28]	133	38	Depression (>16 on CES-D)	Leapfrog	0 (set at 1)	Temperature			Naive RL
Brown et al. 2018[15]	68	39	Veterans with PTSD, or non-veterans with MDD	Two-armed bandit	2 (reward and punishment)	2 inverse temperatures (fixed)	2 (reward and punishmen t)	Associability (for punishment only)	Reward and punishment blocks separate
Cavanagh et al. 2019[29]	121	46	MDD – either low BDI (<7) or high BDI (≥13)	Probabilistic selection task (stimulus pairs)	2 (reward and punishment)	Inverse temperature			
Chase et al. 2010[30]	35	18	MDD outpatients	Probabilistic selection task (stimulus pairs)	2 (reward and punishment)	Temperature (called exploration/exp loitation)			Learning rates differ as function of feedback
Dombrovski et al. 2010[7]	54	40	Suicide attemptors, ideators, MDD	Probabilistic reversal learning task	2 (reward and punishment)	Inverse temperature		Memory	If participants also in 2013 dataset, were removed
Dombrovski et al. 2013/15[8,11]	53	33	Suicide attemptors, ideators, MDD	Probabilistic reversal learning task	2 (reward and punishment)	Inverse temperature		Memory	
Dombrovski et al. 2019[31]	295	221	Suicide attemptors, ideators, MDD	Probabilistic 3-choice task	2 (reward and omission)	Temperature		Decay	
Frey et al. 2019[32]	92	40	High and low BDI scores (>17, <7)	Social probabilistic task with	1	Temperature		Choice bias, outcome valuation	

				fixed contingencies					
Gagne et al. 2020[33]	86	32	GAD or MDD (determined by SCID)	Volatile probabilistic decision making task	7 learning rates	7 inverse temperature	3 subjective magnitude parameter (sensitivity)	7*Mixture parameter, 1 update rate for choice kernel, 3 inverse temperature for choice kernel	Written as components – when 7, effects were (baseline, reward and punishment, good outcome and bad outcome, volatile and stable), when 3, effects were (baseline, reward and punishment), or 1 (just baseline)
Gradin et al. 2011[34]	32	15	MDD diagnosis (removed schizophrenia participants)	Instrumental reward learning	1	Inverse temperature			
Huang et al. 2017[9]	122	77	Anxiety: low (OASIS score ≤8) or high (OASIS score ≥9)	Change point detection	2 (baseline, and higher when maximum value changes)	Inverse temperature			VMax model (learning rate increases if option with maximal value changes)
Huys et al. 2013[18]	224	25	MDD	Probabilistic reward task	1		Outcome sensitivity,	Belief, initial value	'Belief' model

							Instruction sensitivity		
Khdour et al. 2016[35]	73	55	GAD, Social Anxiety Disorder, Panic Disorder	Probabilistic classification task	1	1			No individual-level parameters
Kumar et al. 2018[14]	51	25	MDD	Two-armed bandit	2 (reward and punishment)	2 (reward and punishment)			
Kunisato et al. 2012[36]	18	55	GAD, Social Anxiety Disorder, Panic Disorder	Probabilistic classification task	1	Inverse temperature			
Lamba et al. 2020[13]	354	97	Anxiety (score of ≥10 on GAD-7)	Three-armed bandit		Inverse temperature		Bias, 4 Decay (positive and negative outcomes, and for baseline and as a function of change points)	Dynamic Bayesian RL
Liu et al. 2017[37]	38	21	MDD outpatients	Probabilistic selection task (stimulus pairs)	1	Temperature			
Millner et al. 2019[2]	129	85	Veterans with suicidal thoughts and behaviours	Go/No-go fractal task	1			$\beta 0$, $\beta 1$, w1, w2, ω, τ,	RL-DDM model
Mkrtchian et al. 2017[19]	101	43	Mood/anxiety disorders	Go/No-go task	2 (reward and punishment)	Lapse	2 (reward and punishmen t)	Action bias; 2 Pavlovian bias (approach/avo id)	
Moutoussis et al. 2018[26]	61	39	MDD outpatients	Go/No-go task	1	Lapse	2 (reward and punishmen t)	Action bias; Pavlovian bias	

Mukherjee et al. 2020[10]	128	64	MDD	Probabilistic Reversal task	2 (reward and punishment)	4 (actions + fractals, reward + punishment)	0	8 (four bias terms for reward and punishment)	
Myers et al. 2013[38]	87	48	Veterans with PTSD (≥50 on PTSD-Checklist – Military version) or without (≤50)	Probabilistic classification task	2 (reward and punishment)	Inverse temperature		Free parameter for no feedback i.e. not 0)	
Ross et al. 2018[12]	29	15	Women with PTSD after assaultative violence	Two-arm bandit task	2 (reward and punishment)	Inverse temperature			Anticorrelated updating
Rupprechter et al. 2018[39]	32	15	MDD	Pavlovian conditioning task		Inverse temperature		Memory	Leaky beta model
Rupprechter et al. 2020[40]	433	26	MDD	Probabilistic reward learning task (with no-choice)	2 (choice and no-choice options)	Inverse temperature			
White et al. 2017[41]	79	41	GAD	Passive avoidance task	1	Inverse temperature			Not full sample available

Supplementary Table 3: Details of the studies included in the meta-analytic simulation.

**Authors**	**Title**	**Journal**	**Year**	**DOI/URL**
**Rothkirch et al.**	Neural mechanisms of reinforcement learning in unmedicated patients with major depressive disorder	Brain	2017	10.1093/brain/awx025
**Huys et al.**	Psychiatry: Insights into depression through normative decision-making models	Advances in neural information processing systems	2009	**http://papers.nips.cc/paper/3563-psychiatry-insights-into-depression-through-normative-decision-making-models.pdf**
**Beevers et al.**	Influence of depression symptoms on history-independent reward and punishment processing	Psychiatry Research	2013	https://doi.org/10.1016/j.psychres.2012.09.054

Supplementary Table 4: Details of studies that fit criteria 1-4, but did not report sufficient statistics or individual-level parameter values for all parameters in the reported reinforcement-learning model, and were unable to provide dataset on request or did not respond to our request.

2.2 Assessment of Study Quality

Authors and year	Selection				Comparability				Total
	Disorder definition	Disorder Generality	HC selection	HC definition	Age	Gender	IQ	Inc/Occ	
Aylward et al. 2019[16]	A	A	A	A	Y	Y	Y	N	22
Blanco et al. 2013[28]	A	A	A	C	N	N	N	N	14
Brown et al. 2018[15]	A	B (veterans)	A	A	Y	Y	S	N	20
Cavanagh et al. 2019[29]	A	A	A	A	N	N	N	N	16
Chase et al. 2010[30]	A	B (recent onset)	B	A	Y	Y	Y	N	20
Dombrovski et al. 2010[7]	A	B (elderly)	A	A	Y	Y	Y	N	21
Dombrovski et al. 2013/15[8,11]	A	B (elderly)	A	C	Y	Y	Y	N	19
Dombrovski et al. 2019[31]	A	B (elderly)	A	A	S	S	S	N	18
Frey et al. 2019[32]	A	A	A	C	Y	N	N	N	16
Gagne et al. 2020[33]	A	A	A	B	U	U	U	U	15
Gradin et al. 2011[34]	A	A	A	C	Y	Y	Y	N	20
Huang et al. 2017[9]	A	A	A	C	U	U	U	U	14
Huys et al. 2013[18]	B	A	C	C	N	N	N	N	11
Khdour et al. 2016[35]	A	A	B	B	Y	Y	Y	N	20
Kumar et al. 2018[14]	A	A	A	A	Y	Y	N	N	20
Kunisato et al. 2012[36]	A	A	A	C	Y	Y	N	N	18
Lamba et al. 2020[13]	A	A	A	C	N	N	N	N	14
Liu et al. 2017[37]	A	A	B	A	Y	Y	Y	N	21
Millner et al. 2019[2]	A	B (suicidal)	A	A	Y	Y	Y	N	21
Mkrtchian et al. 2017[19]	A	A	A	A	N	N	N	N	18
Moutoussis et al. 2018[26]	A	A	B	C	Y	Y	N	N	17
Mukherjee et al. 2020[10]	A	A	B	A	Y	Y	Y	N	21
Myers et al. 2013[38]	A	B (veterans)	A	B	Y	Y	N	N	18
Ross et al. 2018[12]	A	C	C	A	Y	Y	N	N	16
Rupprechter et al. 2018[39]	A	A	A	A	Y	Y	Y	N	22
Rupprechter et al. 2020[40]	A	A	A	B	N	N	N	N	15
White et al. 2017[41]	A	C	C	A	Y	Y	Y	N	18

Supplementary Table 5: Table illustrating our assessment of study quality along the dimensions of selection and comparability. The criteria used in assigning these scores are described in Section 1.3.1 above.

2.3 Conventional Meta-Analysis: Heterogeneity and Publication Bias

There was evidence of heterogeneity in studies that estimated learning rate (τ^2 = 0.267; I^2 = 54.5%), inverse temperature (τ^2 = 0.035; I^2 = 40.9%), reward learning rate (τ^2 = 0.033; I^2 = 39.0%), and punishment learning rate (τ^2 = 0.197; I^2 = 79.0%).

For learning rate, the number of studies included was too small to reliably test for small-study effects (k=9). Eggers' test was not significant for inverse temperature (intercept=-0.1165 [-0.4968, 0.2638], t_{17}=-0.50, p=0.625), reward learning rate (intercept=--0.365 [-0.881, 0.151], t_{12}=0.81, p=0.433) or punishment learning rate (intercept=0.0205 [-0.888, 0.929], t_{12}=-0.12, p=0.905).

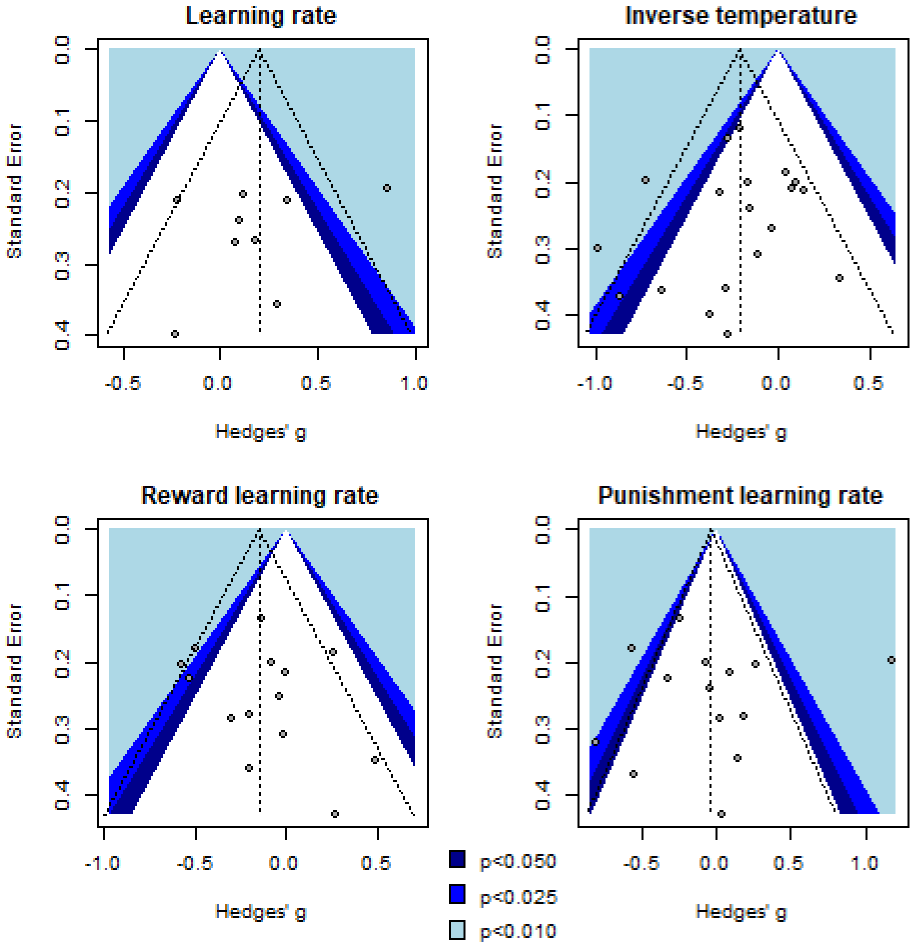

Supplementary Figure 4: Funnel plots for the difference between patients and controls on commonly-reported reinforcement-learning parameters, with contours showing alpha values of p<0.05, p<0.025, p<0.01.

2.4 Effect Size Recovery

To test the sensitivity of this method, we generated parameters for three different 'ground truth' models: one with one learning rate and one inverse temperature, one with two learning rates and one inverse temperature, and a final model with one learning rate, two sensitivity parameters, and a lapse parameter. We then used these ground truth models to generate choices, and subsequently we fitted

the set of models listed in the Supplementary Methods section above (Section 1.6) using variational Bayes with separate group-level priors.

We subsequently performed our Bayesian Model Averaging pipeline, and calculated Cohen's d for parameters that were present in the 'ground-truth' model. Notably, we are largely able to replicate our ground truth differences (albeit with reduced effect sizes) where these are present (for the learning rate in the first model, the reward learning rate in the second, and punishment sensitivity in the latter). Recovery when the generating model is one with a learning rate, two sensitivity parameters and a lapse parameter is weaker, possibly as many of the models that we fit to the simulated data (8/16 for tasks 1-4, 14/22 for task 5) do not include sensitivity parameters, and, correspondingly, sensitivity parameters are not very highly represented in Bayesian Model Averaging.

Ground Truth Model	Parameter	Ground truth effect size	Bayesian Model Averaging effect size
**1lr1b**	Learning rate	0.501	0.884
	Inverse temperature	0.000	-0.009
**2lr1b**	Reward learning rate	0.500	0.250
	Punishment learning rate	0.000	0.005
	Inverse temperature	-0.001	0.000
**1lr2s1lapse**	Learning rate	0.000	0.002
	Reward sensitivity	-0.001	-0.001
	Punishment sensitivity	0.500	0.025
	Lapse	-0.001	0.051

Supplementary Table 6: Effect size recovery for the simulation method used in this paper based on parameters with a known effect size. Here, we used three ground truth models and defined effect size differences between patients and controls on the parameters included in these models (the 'Ground truth' effect size column. We ran these models forward to generate choices. We subsequently fit all the models listed in section 1.6 to these choices using variational Bayes with group-level priors, and then performed Bayesian Model Averaging. Then, we report the effect size of those ground-truth parameters after this pipeline has been run (Bayesian Model Averaging effect size column). Comparing the two effect size columns should give a sense of how well our simulation method is able to reproduce effect sizes from known 'ground truth' parameter distributions.

2.5 Full Univariate Results

2.5.1 Variational Bayes, Single Prior

Variable	Factor	F value	Df	Pr(>F)
**alpha**	group	1.214390e+03	1	0.0000000
**alpha**	task	1.351171e+04	4	0.0000000
**alpha**	study	2.317784e+04	26	0.0000000
**beta**	group	6.453653e+00	1	0.0110725
**beta**	task	4.074445e+03	4	0.0000000
**beta**	study	4.936379e+03	26	0.0000000
**beta_reward**	group	1.275076e+02	1	0.0000000
**beta_reward**	task	3.439008e+03	4	0.0000000
**beta_reward**	study	7.277380e+02	26	0.0000000

**beta_punishment**	group	1.287215e+02	1	0.0000000
**beta_punishment**	task	1.416035e+04	4	0.0000000
**beta_punishment**	study	4.023729e+03	26	0.0000000
**alpha_reward**	group	1.282416e+02	1	0.0000000
**alpha_reward**	task	1.606824e+04	4	0.0000000
**alpha_reward**	study	1.289527e+04	26	0.0000000
**alpha_punishment**	group	1.462630e+03	1	0.0000000
**alpha_punishment**	task	2.809896e+04	4	0.0000000
**alpha_punishment**	study	1.092679e+04	26	0.0000000
**sensitivity**	group	1.306259e+01	1	0.0003013
**sensitivity**	task	3.263366e+03	4	0.0000000
**sensitivity**	study	4.567831e+03	26	0.0000000
**sensitivity_reward**	group	9.975192e+01	1	0.0000000
**sensitivity_reward**	task	8.586441e+00	4	0.0000006
**sensitivity_reward**	study	1.494993e+03	26	0.0000000
**sensitivity_punishment**	group	2.086069e+02	1	0.0000000
**sensitivity_punishment**	task	2.048340e+04	4	0.0000000
**sensitivity_punishment**	study	2.529044e+04	26	0.0000000
**lapse**	group	1.485130e+01	1	0.0001163
**lapse**	task	3.704902e+05	4	0.0000000
**lapse**	study	2.051982e+03	26	0.0000000
**decay**	group	3.462608e+00	1	0.0627713
**decay**	task	1.900295e+04	4	0.0000000
**decay**	study	4.903090e+01	26	0.0000000
**perseverance**	group	1.796585e+03	1	0.0000000
**perseverance**	task	1.796745e+04	4	0.0000000
**perseverance**	study	1.371395e+04	26	0.0000000
**go_bias**	group	1.946854e+02	1	0.0000000
**go_bias**	task	6.352141e+05	4	0.0000000
**go_bias**	study	1.366210e+03	26	0.0000000
**pav_bias**	group	6.369062e-01	1	0.4248333
**pav_bias**	task	3.129545e+05	4	0.0000000
**pav_bias**	study	2.813346e+00	26	0.0000023
**app_bias**	group	2.451000e-04	1	0.9875079
**app_bias**	task	3.135052e+05	4	0.0000000
**app_bias**	study	4.625700e-03	26	1.0000000
**av_bias**	group	1.156381e+00	1	0.2822173
**av_bias**	task	3.037466e+05	4	0.0000000
**av_bias**	study	6.254684e+00	26	0.0000000

2.5.2 Variational Bayes, Group Priors

Variable	Factor	F value	Df	Pr(>F)
**alpha**	group	2.303450e+02	1	0.0000000
**alpha**	task	6.038212e+04	4	0.0000000
**alpha**	study	1.851452e+04	26	0.0000000
**beta**	group	8.476239e+00	1	0.0035982
**beta**	task	5.757897e+02	4	0.0000000

**beta**	study	4.132215e+02	26	0.0000000
**beta_reward**	group	6.795699e+00	1	0.0091378
**beta_reward**	task	4.395351e+00	4	0.0014896
**beta_reward**	study	1.375055e+00	26	0.0963363
**beta_punishment**	group	9.298980e+00	1	0.0022928
**beta_punishment**	task	5.030848e+00	4	0.0004722
**beta_punishment**	study	1.879699e+00	26	0.0042754
**alpha_reward**	group	3.105206e+03	1	0.0000000
**alpha_reward**	task	1.041607e+05	4	0.0000000
**alpha_reward**	study	1.576982e+04	26	0.0000000
**alpha_punishment**	group	3.234696e+04	1	0.0000000
**alpha_punishment**	task	1.671654e+05	4	0.0000000
**alpha_punishment**	study	8.955383e+03	26	0.0000000
**sensitivity**	group	3.957906e+00	1	0.0466517
**sensitivity**	task	2.659825e+00	4	0.0309314
**sensitivity**	study	4.684545e-01	26	0.9901133
**sensitivity_reward**	group	1.841662e+00	1	0.1747563
**sensitivity_reward**	task	3.442152e+00	4	0.0080714
**sensitivity_reward**	study	1.742414e+00	26	0.0109022
**sensitivity_punishment**	group	8.806860e+00	1	0.0030010
**sensitivity_punishment**	task	8.893274e+00	4	0.0000004
**sensitivity_punishment**	study	1.597199e+00	26	0.0274456
**lapse**	group	1.204013e+04	1	0.0000000
**lapse**	task	1.459241e+05	4	0.0000000
**lapse**	study	2.511141e+03	26	0.0000000
**decay**	group	7.901558e+01	1	0.0000000
**decay**	task	1.130152e+04	4	0.0000000
**decay**	study	5.820836e+01	26	0.0000000
**perseverance**	group	2.486160e+03	1	0.0000000
**perseverance**	task	2.157293e+04	4	0.0000000
**perseverance**	study	1.299013e+04	26	0.0000000
**go_bias**	group	2.914030e+02	1	0.0000000
**go_bias**	task	3.971967e+05	4	0.0000000
**go_bias**	study	9.929532e+02	26	0.0000000
**pav_bias**	group	2.281952e+00	1	0.1308871
**pav_bias**	task	2.090304e+05	4	0.0000000
**pav_bias**	study	1.423509e+01	26	0.0000000
**app_bias**	group	2.880080e+01	1	0.0000001
**app_bias**	task	7.594792e+04	4	0.0000000
**app_bias**	study	1.540500e-03	26	1.0000000
**av_bias**	group	4.267781e+00	1	0.0388416
**av_bias**	task	7.510847e+04	4	0.0000000
**av_bias**	study	3.456908e-01	26	0.9992044

2.5.3 Maximum A-Posteriori, Single Prior

Variable	Factor	F value	Df	Pr(>F)
**alpha**	group	9.847825e+02	1	0.0000000
**alpha**	task	1.839612e+04	4	0.0000000
**alpha**	study	1.175841e+04	26	0.0000000
**beta**	group	5.590719e+00	1	0.0180559
**beta**	task	1.517679e+02	4	0.0000000
**beta**	study	1.872758e+02	26	0.0000000
**beta_reward**	group	3.774078e+01	1	0.0000000
**beta_reward**	task	5.590561e+01	4	0.0000000
**beta_reward**	study	2.532236e+01	26	0.0000000
**beta_punishment**	group	3.538367e+01	1	0.0000000
**beta_punishment**	task	5.521180e+01	4	0.0000000
**beta_punishment**	study	2.633381e+02	26	0.0000000
**alpha_reward**	group	1.165428e+01	1	0.0006406
**alpha_reward**	task	9.309521e+04	4	0.0000000
**alpha_reward**	study	9.286729e+03	26	0.0000000
**alpha_punishment**	group	3.915375e+03	1	0.0000000
**alpha_punishment**	task	4.698586e+04	4	0.0000000
**alpha_punishment**	study	1.313498e+04	26	0.0000000
**sensitivity**	group	9.646359e+01	1	0.0000000
**sensitivity**	task	6.498502e+01	4	0.0000000
**sensitivity**	study	1.219736e+02	26	0.0000000
**sensitivity_reward**	group	3.673740e+01	1	0.0000000
**sensitivity_reward**	task	5.841093e+01	4	0.0000000
**sensitivity_reward**	study	1.269481e+02	26	0.0000000
**sensitivity_punishment**	group	1.097354e-01	1	0.7404456
**sensitivity_punishment**	task	4.011837e+01	4	0.0000000
**sensitivity_punishment**	study	5.486666e+01	26	0.0000000
**lapse**	group	6.462900e-03	1	0.9359253
**lapse**	task	1.774588e+05	4	0.0000000
**lapse**	study	1.913682e+03	26	0.0000000
**decay**	group	2.327576e+02	1	0.0000000
**decay**	task	5.416478e+04	4	0.0000000
**decay**	study	5.349856e+02	26	0.0000000
**perseverance**	group	2.585237e+01	1	0.0000004
**perseverance**	task	1.175514e+02	4	0.0000000
**perseverance**	study	8.625106e+01	26	0.0000000
**go_bias**	group	1.038521e+02	1	0.0000000
**go_bias**	task	1.468925e+01	4	0.0000000
**go_bias**	study	7.472874e+01	26	0.0000000
**pav_bias**	group	3.536827e+01	1	0.0000000
**pav_bias**	task	4.864079e+01	4	0.0000000
**pav_bias**	study	1.112456e+02	26	0.0000000

**app_bias**	group	3.581951e+01	1	0.0000000
**app_bias**	task	9.994258e+01	4	0.0000000
**app_bias**	study	4.723452e+01	26	0.0000000
**av_bias**	group	1.378884e+01	1	0.0002045
**av_bias**	task	1.541414e+02	4	0.0000000
**av_bias**	study	6.254147e+01	26	0.0000000

2.5.4 Maximum A-Posteriori, Group Priors

Variable	Factor	F value	Df	Pr(>F)
**alpha**	group	1.952081e+03	1	0.0000000
**alpha**	task	3.951651e+04	4	0.0000000
**alpha**	study	1.203627e+04	26	0.0000000
**beta**	group	1.305739e+02	1	0.0000000
**beta**	task	2.442069e+02	4	0.0000000
**beta**	study	1.417763e+02	26	0.0000000
**beta_reward**	group	1.251845e+02	1	0.0000000
**beta_reward**	task	3.914597e+02	4	0.0000000
**beta_reward**	study	1.362766e+03	26	0.0000000
**beta_punishment**	group	1.170984e+02	1	0.0000000
**beta_punishment**	task	3.812045e+02	4	0.0000000
**beta_punishment**	study	1.363351e+03	26	0.0000000
**alpha_reward**	group	1.665936e+02	1	0.0000000
**alpha_reward**	task	4.149728e+04	4	0.0000000
**alpha_reward**	study	1.459022e+04	26	0.0000000
**alpha_punishment**	group	5.514633e+03	1	0.0000000
**alpha_punishment**	task	4.587283e+04	4	0.0000000
**alpha_punishment**	study	1.471260e+04	26	0.0000000
**sensitivity**	group	7.727588e+01	1	0.0000000
**sensitivity**	task	4.162782e+01	4	0.0000000
**sensitivity**	study	2.591338e+01	26	0.0000000
**sensitivity_reward**	group	4.405627e-01	1	0.5068510
**sensitivity_reward**	task	1.036535e+02	4	0.0000000
**sensitivity_reward**	study	4.442463e+02	26	0.0000000
**sensitivity_punishment**	group	5.999143e+00	1	0.0143128
**sensitivity_punishment**	task	2.047053e+02	4	0.0000000
**sensitivity_punishment**	study	5.796791e+02	26	0.0000000
**lapse**	group	1.106212e+03	1	0.0000000
**lapse**	task	2.780625e+05	4	0.0000000
**lapse**	study	8.649435e+03	26	0.0000000
**decay**	group	6.174732e+02	1	0.0000000
**decay**	task	4.358027e+04	4	0.0000000
**decay**	study	1.447981e+03	26	0.0000000
**perseverance**	group	1.965309e+01	1	0.0000093
**perseverance**	task	6.503515e+01	4	0.0000000

**perseverance**	study	1.322247e+02	26	0.0000000
**go_bias**	group	2.175903e+01	1	0.0000031
**go_bias**	task	1.433563e+03	4	0.0000000
**go_bias**	study	5.250998e+01	26	0.0000000
**pav_bias**	group	7.420497e+01	1	0.0000000
**pav_bias**	task	5.015732e+01	4	0.0000000
**pav_bias**	study	6.823301e+01	26	0.0000000
**app_bias**	group	2.830973e+01	1	0.0000001
**app_bias**	task	4.213569e+01	4	0.0000000
**app_bias**	study	5.371783e+01	26	0.0000000
**av_bias**	group	4.771000e-03	1	0.9449317
**av_bias**	task	4.340361e+02	4	0.0000000
**av_bias**	study	6.216055e+01	26	0.0000000

2.6 Sub-Group Analyses

After performing an illustrative fixed-effects meta-analysis across analysis approaches for the most-represented parameters (learning rates and sensitivity parameters), we also performed subgroup analyses for different diagnoses – depression (k=15), anxiety (k=5), PTSD (k=4) and mixed anxiety/depression (k=3).

2.6.1 Learning Rate

Subgroup analyses indicated that this differed between diagnosis (Q_3= 29466.65, p<0.001): those with anxiety showed an increased learning-rate (0.0689[0.0672, 0.0706]) and those with depression showed a decreased learning rate (-0.0613[-0.0625, -0.0602]). Whilst a limited number of studies focused on PTSD or recruited a mixed anxiety/depression sample, the results from these also indicated an increased learning rate (PTSD: 0.0628 [0.0598; 0.0657]; mixed: 0.1193[0.1169; 0.1216]).

2.6.2 Reward learning rate

For reward learning rate, there was also a significant effect of diagnosis (Q_3 = 16681.01, p<0.001): here, those with depression also showed a reduction in learning rate (-0.0573 [-0.0584; -0.0562]), as did those with PTSD (-0.0056 [-0.0084; -0.0028]), but those with anxiety showed an increase (0.0665 [0.0649; 0.0681]), as did mixed depression and anxiety groups (0.0230 [0.0208; 0.0253]).

2.6.3 Punishment learning rate

Similarly, there was a significant effect of diagnosis on punishment learning rate (Q_3= 22255.95, p<0.001), although all groups showed an increase (depression: 0.0137 [0.0126; 0.0147]; anxiety: 0.1225 [0.1209; 0.1241]; PTSD: 0.1311 [0.1283; 0.1340]; mixed: 0.1588 [0.1565; 0.1610]).

2.6.4 Inverse temperature

There was an effect of diagnosis on inverse temperature: Q_3= 3822.06, p<0.001. All groups except PTSD showed reduced inverse temperature (PTSD: 0.0007 [-0.0024; 0.0038]; depression: -0.0469 [-0.0481; -0.0458]; anxiety: -0.0236 [-0.0254; -0.0218]; mixed: -0.1069 [-0.1093; -0.1044]).

2.7 META-REGRESSIONS

2.7.1 Study quality

We converted our modified Newcastle-Ottawa ratings into continuous scores, mean-centred them, and added them to meta-regressions. The estimates and their p-values are shown below (Supplementary Table 7). We also present the adjusted effect size and confidence intervals (i.e. the intercept when the covariate is mean-centred[42]). After controlling for study quality, the effects on reward and punishment learning rate remain significant; the effect on learning rate is no longer significant, and there is an effect on inverse temperature, such that patients show lower inverse temperature than controls.

	Effect of adding quality to meta-regression			Adjusted effect size of group on parameter value		
Parameter	Effect	Z value	p value	Adjusted effect size	Lower confidence interval	Upper confidence interval
Learning rate	-0.0314	-3.0044	0.0027	-0.0154	-0.0358	0.0050
Reward learning rate	-0.0388	-4.6086	<.0001	-0.0288	-0.0453	-0.0123
Punishment learning rate	-0.0236	-2.4986	0.0125	0.0577	0.0392	0.0762
Inverse temperature	-0.0090	-1.4569	0.1451	-0.0530	-0.0651	-0.0409

Supplementary Table 7: Estimated effects, z-values and p-values for the effect of study quality on effect sizes.

2.7.2 Year of publication

Similarly, we performed a meta-regression using mean-centred year of publication, the results for which are shown in Supplementary Table 8. Again, the effects on reward and punishment learning rate remain the same. The effect on learning rate is no longer significant, but there is an effect on inverse temperature, such that patients show lower inverse temperature than controls.

	Effect of adding year of publication to meta-regression			Adjusted effect size of group on parameter value		
Parameter	Effect	Z value	p value	Adjusted effect size	Lower confidence interval	Upper confidence interval
Learning rate	0.0130	3.9830	<.0001	-0.0154	-0.0358	0.0050
Reward learning rate	0.0104	3.9308	<.0001	-0.0288	-0.0454	-0.0122
Punishment learning rate	0.0070	2.3747	0.0176	0.0577	0.0391	0.0762
Inverse temperature	-0.0132	-6.8541	<.0001	-0.0529	-0.0649	-0.0409

Supplementary Table 8: Estimated effects, z-values and p-values for the effect of year of publication on effect sizes.

2.7.3 Parameter uncertainty

Relative parameter uncertainty summarizes the extent to which the estimates of the parameter are imprecise. Higher imprecision in a parameter estimate could indicate poor model fit, either due to a different underlying generative process, insufficient data, or inadequate parameterization. For each combination of task, model, and analysis method, we estimated the relative uncertainty for the parameters that were most represented in Bayesian Model Averaging (learning rate, reward learning rate, punishment learning rate, and inverse temperature). Relative uncertainty in estimating a parameter for a given participant is defined in the following supplementary equation:

$$Relative\ Uncertainty = \frac{Standard\ Deviation\ of\ Individual's\ Parameter}{Mean\ of\ Individual's\ Parameter}$$

Note that the standard deviation is available to us where a whole posterior distribution is estimated, rather than a point estimate. For this reason, it is not possible to calculate the relative uncertainty at the individual level for maximum a-posteriori estimation, and the uncertainty at the group level just reflects the spread of the parameter, rather than any uncertainty inherent to the estimation method/model. We therefore show results for a meta-regression including these relative uncertainty terms for parameter estimates using variational Bayes in Supplementary Table 9. The effect on punishment learning rate remains the same, whereas the effects on reward and single learning rate are no longer significant. There is no significant effect on inverse temperature.

	Effect of adding relative uncertainty to meta-regression			Adjusted effect size of group on parameter value		
Parameter	Effect	Z value	p value	Adjusted effect size	Lower confidence interval	Upper confidence interval
Learning rate	-0.0042	-0.1566	0.8756	0.0804	-0.0148	0.1755
Reward learning rate	0.0137	0.1413	0.8876	-0.0420	-0.1567	0.0727
Punishment learning rate	0.0009	0.0788	0.9372	0.1367	0.0019	0.2715
Inverse temperature	-0.1004	-1.0364	0.3000	0.0512	-0.0847	0.1871

Supplementary Table 9: Estimated effects, z-values and p-values for the effect of relative uncertainty in parameter estimation on effect sizes. Note that this meta-regression is only using effect sizes for variational Bayes estimation.

2.8 Task Effects

Notably, there were significant task effects on our parameter estimates, using either Bayesian Model Averaging or (see below) using a winner-takes-all approach. particular, it is worth noting that tasks which contain correlated rewards and punishments result in greater difficulty in estimating separate reward and punishment learning rates, as illustrated using the correlation between synthetic parameters and recovered parameters below (Supplementary Figure 5). It may be that studies using correlated rewards and punishments limited their ability to observe the valence-dependent effects we see in this meta-analysis, hence explaining the lack of learning rate effects on the conventional meta-analysis.

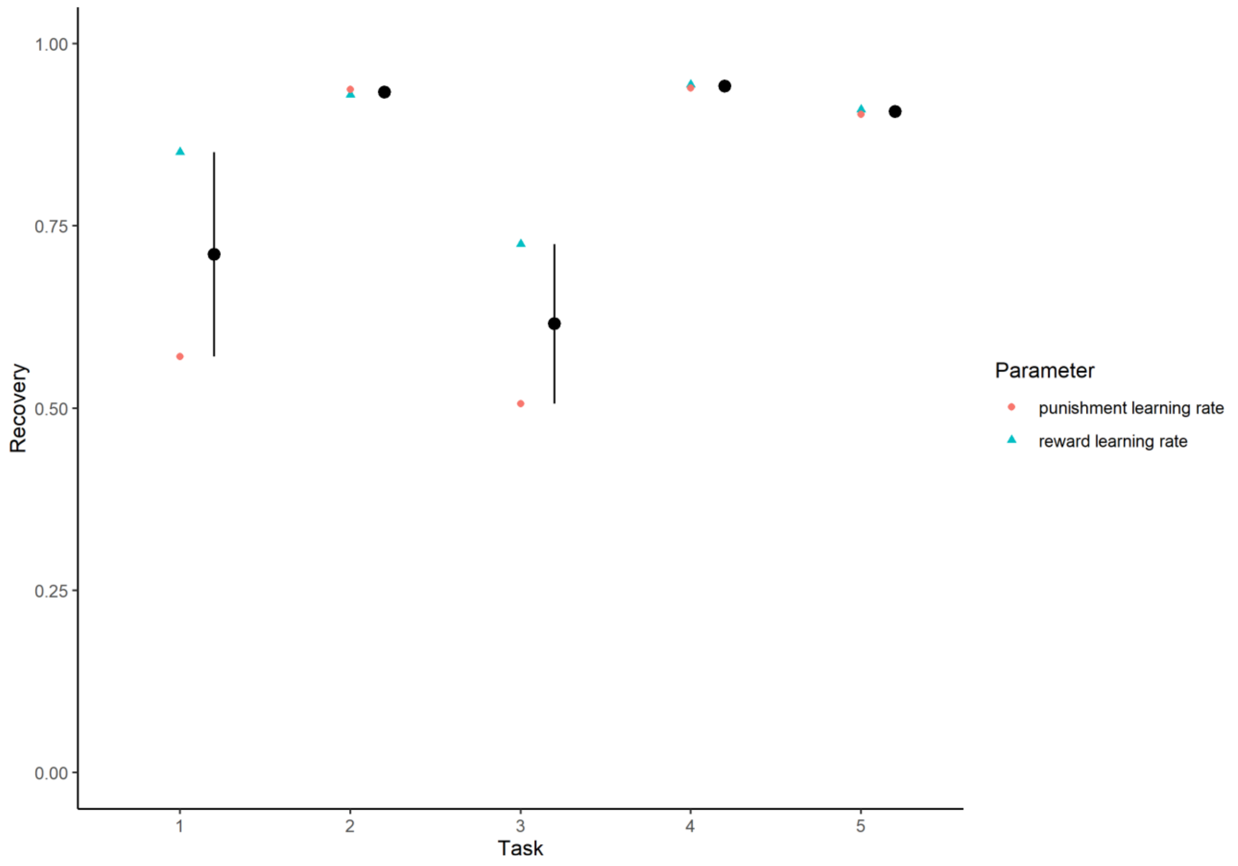

Supplementary Figure 5: The correlation coefficients between synthetic and recovered reward and punishment learning rates by task. Note that perfect recovery of these known parameters would result in a coefficient of 1. It is apparent that recovery varies substantially as a function of task. Tasks 1 and 3 have reward and punishment outcomes which are perfectly correlated; the others do not. As can be seen here, recovery (and hence our ability to trust the parameters) is considerably worse for tasks 1 and 3. Recovery was tested using variational Bayes and a model with two learning rates and an inverse temperature for illustrative purposes. Points in black (shifted right from the main tick marks) represent mean and standard error.

2.9 Task Sensitivity Analysis

Given that the recovery for reward and punishment learning rates was somewhat worse for tasks 1 and 3 (see Supplementary Figure 5), we also ran the simulation meta-analysis excluding these tasks. The results are very similar, indicating that our learning rate findings do not result from noise in the estimation of reward and punishment learning rates. Again, we see a meaningful increase in punishment learning rates (standardized mean difference = 0.0981 [0.0972; 0.0990]), a slight decrease in reward learning rates (-0.0133 [-0.0142; -0.0124]). If only a single learning rate was estimated in a model, this was slightly elevated in patients compared to controls (0.0585 [0.0575; 0.0594]). There was only a negligible effect on inverse temperature (0.0053 [0.0042; 0.0063]).

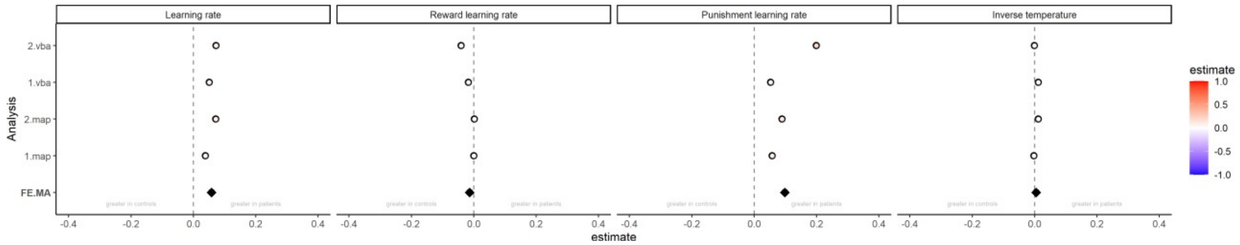

Supplementary Figure 6: Forest plots of the Cohen's d effect sizes for the four most highly-represented parameters from our Bayesian model averaging analysis, across the four analytic strategies we used. This is the same as Figure 3 in the main paper, except that only tasks 2, 4 and 5 (those with better reward/punishment learning rate estimation) are included. On the x-axis is the Cohen's *d* effect size value, and on the y axis is the type of analysis. The plots are split by parameter type and show: A) a small learning rate increase in patients. When learning rates are allowed to be separate for rewards and punishments, we can see B) a slight decrease in reward learning rate and a C) meaningful increase in punishment learning rate in patients compared to controls. D) Unlike the conventional meta-analysis, we see no meaningful difference in inverse temperature. 'VBA' stands for 'variational Bayesian analysis', and 'MAP' stands for 'maximum a posteriori' analysis; '1' refers to the cases where parameters were estimated using a hierarchical Bayesian approach with only a single prior over groups, '2' refers to the cases where a different prior was estimated for each group. At the bottom are the results of the fixed-effects meta-analyses ('FE MA'), with the point estimate for the standardized mean difference (Cohen's *d*) and the 95% confidence interval shown in black. A dashed line is displayed representing an effect size of *d*=0.

2.10 Winner-Takes-All Model Analyses

Here, we present more detail of the results of all analyses performed using different methodological choices, in order to examine the robustness of effects to analytic degrees of freedom. First, we present the results when one ANOVA is performed per task (on the overall winning model), rather than the omnibus ANOVA presented in the main text with parameters derived from all tasks. Secondly, we present the results of performing one ANOVA per task on parameters estimated from the per-task winning model, i.e. the model with the lowest sum of BIC for that task only. Thirdly, we present the results of ANOVAs performed on parameters estimated using a single prior across both groups. Finally, we present the results of ANOVAs estimated using maximum a-posteriori estimation rather than variational inference. A summary of these results can also be seen in Figure 4 in the main text.

2.10.1 Methods

2.10.1.1 Model Comparison

Model comparison was performed to select the model that most parsimoniously described the data (represented by the lowest BIC) across all tasks. We report the log Bayes Factor[43] of the improvement in BIC between the first and second best models. Model selection results are presented in full for each analysis method below. The generate-recover matrix for each task using variational estimation is shown in Supplementary Figure 19 and Supplementary Figure 22.

We used the Bayesian Information Criteria (BIC) to determine the best-fitting model (where the lower the BIC, the better the model fits the data). To compare models, we report the Bayes Factor, which can be calculated from total BIC scores using the following equation:

$$BF_{ab} = \exp\left(\frac{BIC_b - BIC_a}{2}\right)$$

where BF_{ab} refers to the Bayes Factor that indicates the strength of the evidence for model *a* relative to model *b*, and BIC_a refers to the total BIC for model *a*. In general, a Bayes Factor of 1-3 indicates anecdotal evidence, 3-10 indicates substantial evidence, 10-30 indicates strong evidence, and >100 indicates decisive evidence[43].

2.10.1.2 Inference

The posterior means of each separate parameter for all tasks were analysed together, in a 2(Group: patients, controls) x 27(Study) x 5 (Task) ANOVA. A Greenhouse-Geisser correction was used if the assumption of sphericity was violated. Effect sizes are reported as Cohen's d for the comparison across groups to aid interpretation. In Supplementary Section 2.4, we present a simulation showing that given a known between-group effect size for a specific parameter, this method can reliably detect a true between-group difference, albeit that it is likely to be an *overestimate* of the effect.

2.10.2 Variational Bayes, Single Prior

2.10.2.1 Model Selection

The winning model had a single learning rate, which govern the rate that learnt stimulus values are updated by prediction errors, and one inverse temperature parameter, which scales the learnt values of the stimuli, and thus also govern the extent to which participants behave deterministically. This model also had a choice kernel, with a perseverance parameter and a decay parameter. The decay parameter controls how many trials back participants keep track of when deciding whether to repeat a choice or not, and the perseverance parameter governs how likely they are to repeat a previous choice. This model is substantially better than the next model [logBF10 = 44624.72]. More detail on the other models we compared can be found in Section 1.6 of the supplement above, and more detail on the model comparison process can be found above in Section 2.10.1.1.

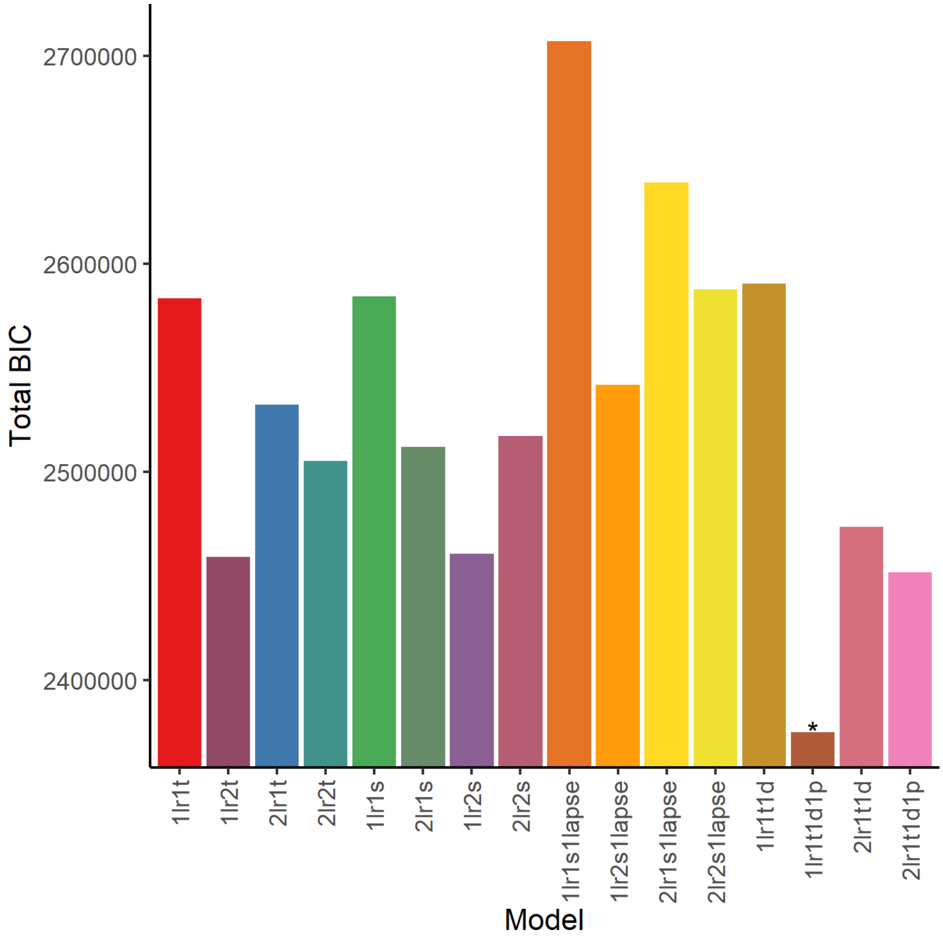

Supplementary Figure 7: Bar graph of BIC for each model after parameter estimation using variational Bayes with a single prior over all participants. The best fitting model is marked with an asterisk.

2.10.2.2 Learning rate

	F	num Df	den Df	Pr(>F)	ges
**pat_con**	0.334	1.000	3057.00	0.563	0.000
**study**	131.515	26.000	3057.00	0.000	0.417
**task**	818.949	3.667	11210.66	0.000	0.088
**pat_con:task**	0.420	3.667	11210.66	0.778	0.000
**study:task**	45.100	95.347	11210.66	0.000	0.122

Cohen's d: 0.05[0.02,0.09].

2.10.2.3 Inverse temperature

	F	num Df	den Df	Pr(>F)	ges
**pat_con**	5.437	1.000	3057.00	0.020	0.001
**study**	172.396	26.000	3057.00	0.000	0.532
**task**	243.727	3.745	11447.92	0.000	0.018
**pat_con:task**	0.548	3.745	11447.92	0.688	0.000
**study:task**	16.610	97.365	11447.92	0.000	0.031

Cohen's d: -0.08[-0.11,-0.05]

2.10.2.4 Decay

	F	num Df	den Df	Pr(>F)	ges
**pat_con**	9.319	1.000	3057.00	0.002	0.001
**study**	41.219	26.000	3057.00	0.000	0.139
**task**	226.608	3.537	10813.49	0.000	0.039
**pat_con:task**	0.674	3.537	10813.49	0.592	0.000
**study:task**	12.180	91.970	10813.49	0.000	0.053

Cohen's d: 0.01[-0.02,0.04]

2.10.2.5 Perseverance

	F	num Df	den Df	Pr(>F)	ges
**pat_con**	3.763	1.000	3057.00	0.052	0.001
**study**	95.571	26.000	3057.00	0.000	0.371
**task**	48.144	3.445	10530.32	0.000	0.004
**pat_con:task**	2.706	3.445	10530.32	0.036	0.000
**study:task**	33.444	89.561	10530.32	0.000	0.072

Cohen's d: -0.09[-0.13,-0.06]

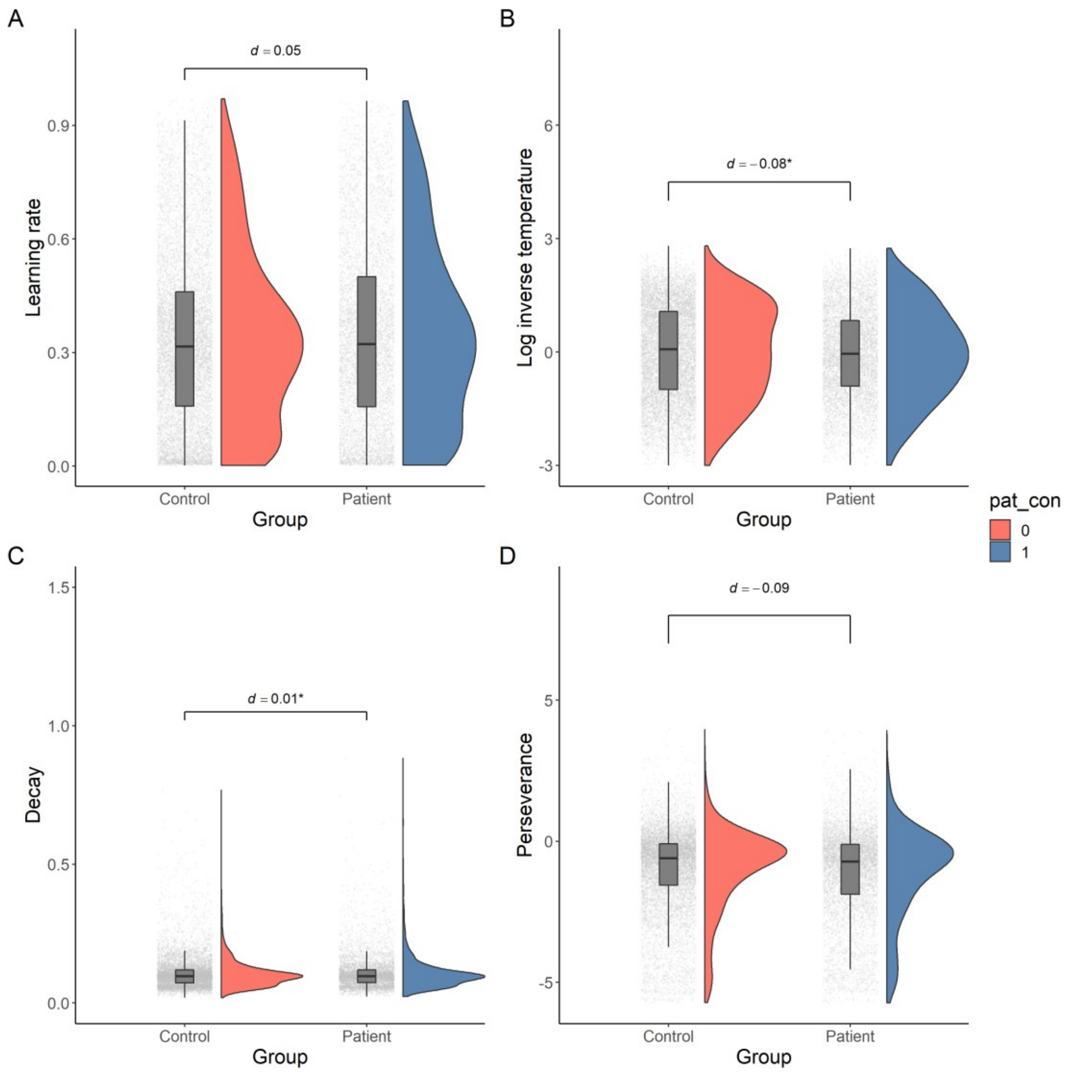

Supplementary Figure 8: Raincloud plots[44] showing the main effects of group on each parameter of the overall winning model fitted using variational Bayes with a single prior across all participants. A) Learning rate. B) Log reward inverse temperature. C) Decay. D) Perseverance. On the left of each tick mark, a boxplot is displayed (showing median, first and third quartiles, with whiskers showing the largest value no more than 1.5*IQR from the third quartile, and the smallest value no more than 1.5*IQR from the first quartile), with the individual data points plotted in grey beneath it. On the right is a half violin plot, showing the density of the distribution. The Cohen's *d* effect size for group effects is displayed above to aid interpretation, and asterisks represent significant main effects of group in the ANOVA.

2.10.2.6 Choice behaviour

Below, we show data that relates our choice bank data, generated from the original models, to data generated from this best-fitting model. We show that the trial-by-trial accuracy from our bank of choices (generated by feeding original parameters through each papers' best-fitting model for benchmarking tasks) tracks trial-by-trial accuracy for choices generated from the best-fitting model, shown averaged over different participants' choices for different tasks (Supplementary Figure 9).

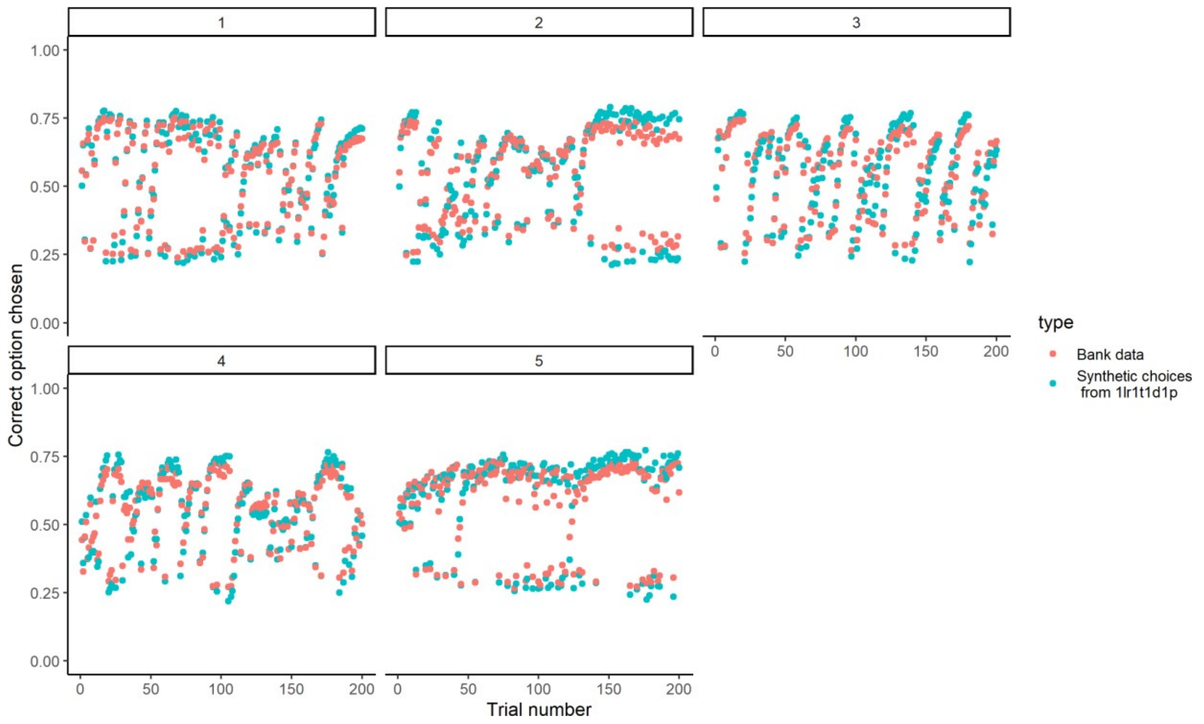

Supplementary Figure 9: Trial-by-trial accuracy (defined as choosing the option with the maximum value on each trial) across participants for each of the tasks, with both the choice bank data (from using original parameters and original models and feeding into the benchmarking tasks) and synthetic choices from the best-fitting model.

2.10.3 Variational Bayes, Separate Priors

2.10.3.1 Model Selection

The winning model had a single learning rate, which governs the rate that learnt stimulus values are updated by prediction errors, and a single inverse temperature, which scale the values of the outcomes received, and thus also govern the extent to which participants behave deterministically, alongside a decay term, which controls how many trials back participants keep track of when deciding whether to repeat a choice or not, and a perseverance parameter, which governs how likely they are to repeat a previous choice. This model is substantially better than the next model [logBF10 = 35807.99]. More detail on the other models we compared can be found in Section 1.6, and more detail on the model comparison process can be found above in Section 2.10.1.1.

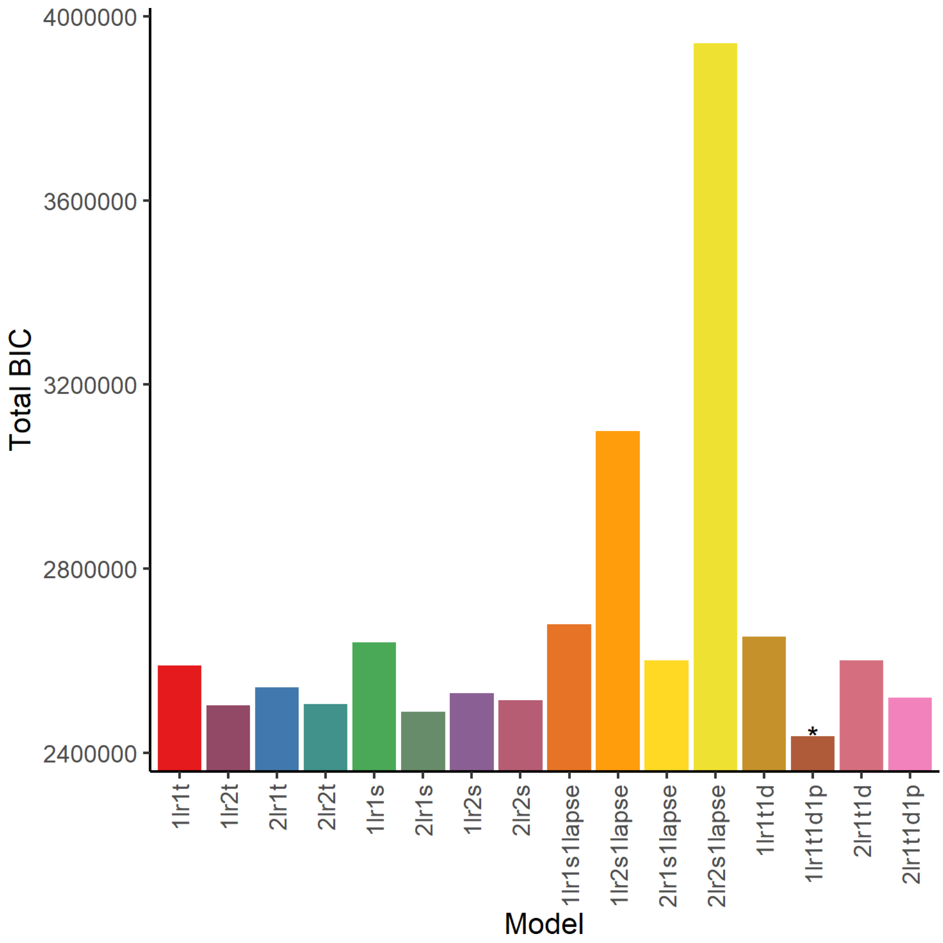

Supplementary Figure 10: Bar graph of BIC for each model after parameter estimation using variational Bayes with separate priors for each group. The best fitting model is marked with an asterisk.

2.10.3.2 Learning rate

	F	num Df	den Df	Pr(>F)	ges
**pat_con**	2.513	1.000	3057.00	0.113	0.001
**study**	131.888	26.000	3057.00	0.000	0.414
**task**	57.448	3.659	11185.12	0.000	0.007
**pat_con:task**	1044.756	3.659	11185.12	0.000	0.112
**study:task**	17.653	95.130	11185.12	0.000	0.053

Cohen's d: 0.11[0.08,0.14]

2.10.3.3 Inverse temperature

	F	num Df	den Df	Pr(>F)	ges
**pat_con**	10.376	1.000	3057.00	0.001	0.003
**study**	175.936	26.000	3057.00	0.000	0.534
**task**	59.281	3.672	11225.97	0.000	0.005
**pat_con:task**	93.071	3.672	11225.97	0.000	0.007
**study:task**	20.826	95.478	11225.97	0.000	0.040

Cohen's d: -0.1[-0.13,-0.07]

2.10.3.4 Decay

	F	num Df	den Df	Pr(>F)	ges
**pat_con**	104.163	1.000	3057.000	0	0.014
**study**	33.174	26.000	3057.000	0	0.106
**task**	127.014	3.066	9372.986	0	0.024
**pat_con:task**	959.508	3.066	9372.986	0	0.154
**study:task**	14.434	79.718	9372.986	0	0.066

Cohen's d: 0.3[0.27,0.34]

2.10.3.5 Perseverance

	F	num Df	den Df	Pr(>F)	ges
**pat_con**	3.496	1.000	3057.00	0.062	0.001
**study**	90.148	26.000	3057.00	0.000	0.360
**task**	14.728	3.425	10470.68	0.000	0.001
**pat_con:task**	95.756	3.425	10470.68	0.000	0.008
**study:task**	33.503	89.054	10470.68	0.000	0.071

Cohen's d: -0.09[-0.12,-0.06]

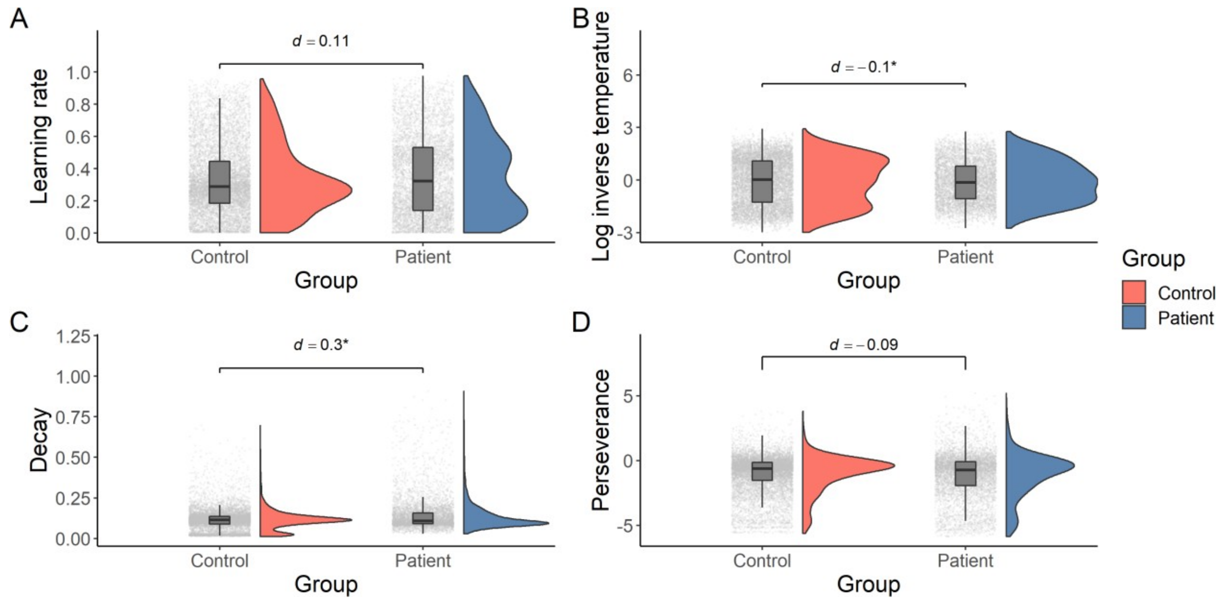

Supplementary Figure 11: Raincloud plots[44] showing the main effects of group on each parameter of the overall winning model fitted using variational Bayes with separate priors for each group. A) Reward learning rate. B) Punishment learning rate. C) Log sensitivity. On the left of each tick mark, a boxplot is displayed (showing median, first and third quartiles, with whiskers showing the largest value no more than 1.5*IQR from the third quartile, and the smallest value no more than 1.5*IQR from the first quartile), with the individual data points plotted in grey beneath it. On the right is a half violin plot, showing the density of the distribution. The Cohen's *d* effect size for group effects is displayed above to aid interpretation, and asterisks represent significant main effects of group in the ANOVA.

2.10.3.6 Choice behaviour

Below, we show data that relates our choice bank data, generated from the original models, to data generated from this best-fitting model. We show that the trial-by-trial accuracy from our bank of choices (generated by feeding original parameters through each papers' best-fitting model for benchmarking tasks) tracks trial-by-trial accuracy for choices generated from the best-fitting model, shown averaged over different participants' choices for different tasks (Supplementary Figure 12).

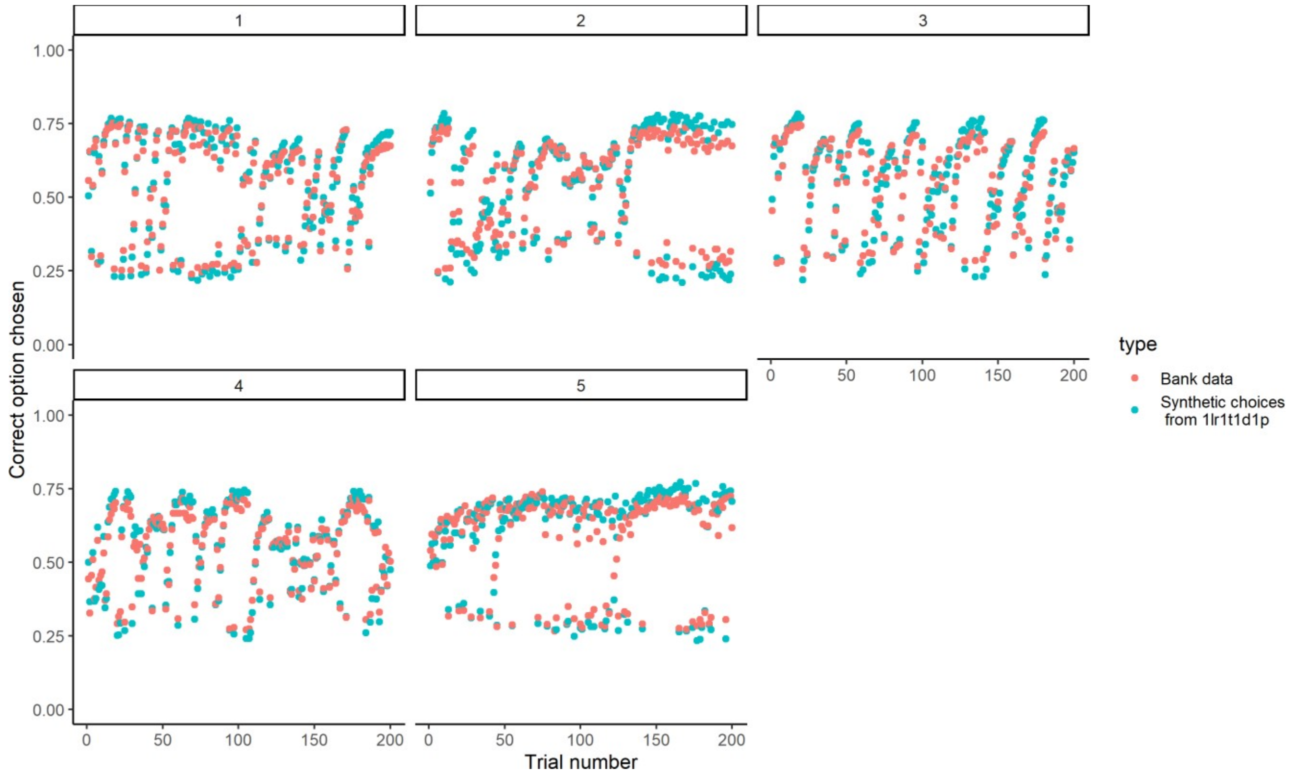

Supplementary Figure 12: Trial-by-trial accuracy (defined as choosing the option with the maximum value on each trial) across participants for each of the tasks, with both the choice bank data (from using original parameters and original models and feeding into the benchmarking tasks) and synthetic choices from the best-fitting model.

2.10.4 Maximum A-Posteriori, Single Prior

2.10.4.1 Model Selection

The winning model had a single learning rate, which governs the rate that learnt stimulus values are updated by prediction errors, a single inverse temperature parameter, which scales the learnt values, and thus also governs the extent to which participants behave deterministically, alongside a decay term, which controls how many trials back participants keep track of when deciding whether to repeat a choice or not, and a perseverance parameter, which governs how likely they are to repeat a previous choice. This model is substantially better than the next model [logBF10 = 16224.56]. More detail on the other models we compared can be found in Section 1.6, and more detail on the model comparison

process can be found above in Section 2.10.1.1.

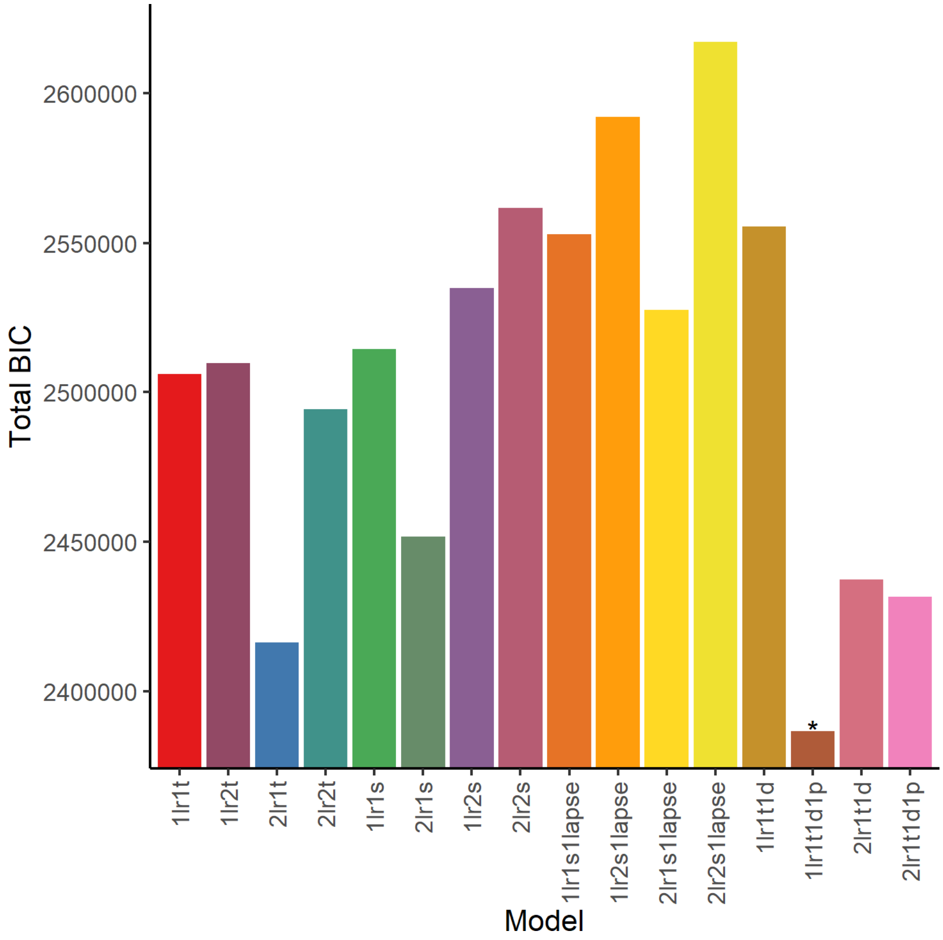

Supplementary Figure 13: Bar graph of BIC for each model after parameter estimation using maximum a-posteriori with a single overall prior. The best fitting model is marked with an asterisk.

2.10.4.2 Reward learning rate

	F	num Df	den Df	Pr(>F)	ges
**pat_con**	90.093	1.000	3057.00	0	0.013
**study**	18.647	26.000	3057.00	0	0.069
**task**	40.584	3.756	11481.03	0	0.007
**pat_con:task**	24.837	3.756	11481.03	0	0.004
**study: task**	4.235	97.647	11481.03	0	0.019

Cohen's d: 0.23[0.2,0.26]

2.10.4.3 Inverse temperature

	F	num Df	den Df	Pr(>F)	ges
**pat_con**	2.030	1.000	3057.000	0.154	0.000
**study**	1.068	26.000	3057.000	0.371	0.002
**task**	1.809	1.019	3114.515	0.179	0.000
**pat_con:task**	2.039	1.019	3114.515	0.153	0.001

**study:task**	1.095	26.489	3114.515	0.335	0.007

Cohen's d: 0.03[0,0.06]

2.10.4.4 Decay

	F	num Df	den Df	Pr(>F)	ges
**pat_con**	168.942	1.000	3057.000	0	0.016
**study**	6.526	26.000	3057.000	0	0.016
**task**	423.930	2.382	7281.062	0	0.088
**pat_con:task**	31.458	2.382	7281.062	0	0.007
**study:task**	3.354	61.926	7281.062	0	0.020

Cohen's d: 0.23[0.2,0.27]

2.10.4.5 Perseverance

	F	num Df	den Df	Pr(>F)	ges
**pat_con**	2.503	1.000	3057.00	0.114	0.000
**study**	1.474	26.000	3057.00	0.058	0.003
**task**	2.604	1.094	3345.71	0.104	0.001
**pat_con:task**	1.887	1.094	3345.71	0.169	0.000
**study:task**	1.551	28.455	3345.71	0.031	0.010

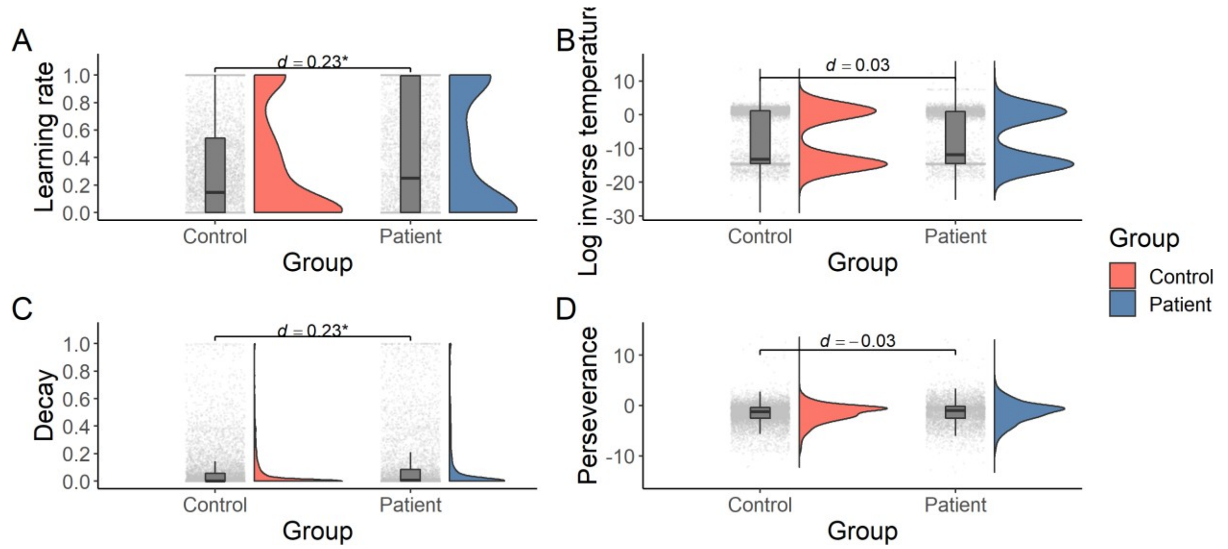

Supplementary Figure 14: Raincloud plots[44] showing the main effects of group on each parameter of the overall winning model fitted using a maximum a-posteriori approach with a single prior. A) Learning rate. B) Log inverse temperature. C) Decay. D) Perseverance. On the left of each tick mark, a boxplot is displayed (showing median, first and third quartiles, with whiskers showing the largest value no more than 1.5*IQR from the third quartile, and the smallest value no more than 1.5*IQR from the first quartile), with the individual data points plotted in grey beneath it. On the right is a half violin plot, showing the density of the distribution. The Cohen's *d* effect size for group effects is displayed above to aid interpretation, and asterisks represent

significant main effects of group in the ANOVA. Note that the y axis for perseverance is truncated to between -15 and +15, where the majority of the mass is.

2.10.4.6 Choice behaviour

Below, we show data that relates our choice bank data, generated from the original models, to data generated from this best-fitting model. We show that the trial-by-trial accuracy from our bank of choices (generated by feeding original parameters through each papers' best-fitting model for benchmarking tasks) tracks trial-by-trial accuracy for choices generated from the best-fitting model, shown averaged over different participants' choices for different tasks (Supplementary Figure 15).

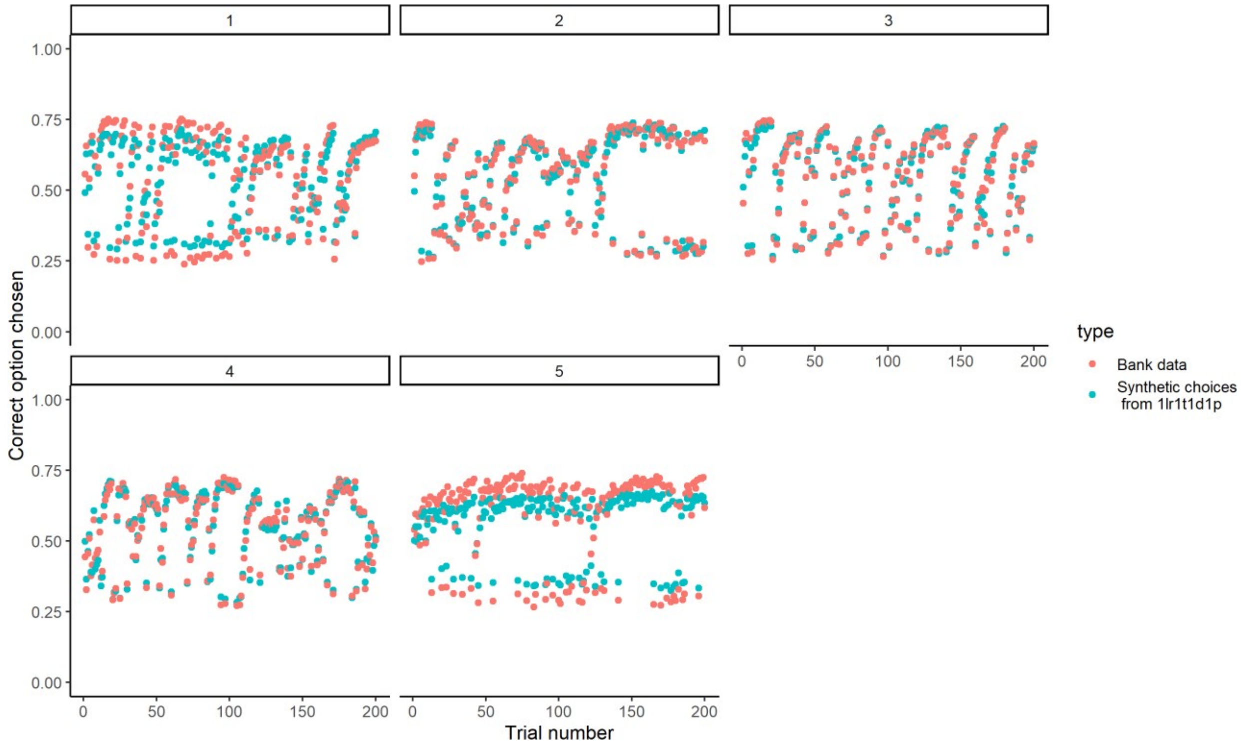

Supplementary Figure 15: Trial-by-trial accuracy (defined as choosing the option with the maximum value on each trial) across participants for each of the tasks, with both the choice bank data (from using original parameters and original models and feeding into the benchmarking tasks) and synthetic choices from the best-fitting model.

2.10.5 Maximum A-Posteriori, Separate Priors

2.10.5.1 Model Selection

The winning model was the same as above, for a single prior: a single learning rate, which governs the rate that learnt stimulus values are updated by prediction errors, and a single inverse temperature parameter, which scale the learnt values, a decay parameter, and a perseverance parameter. This model is substantially better than the next model [logBF10 = 14804.35]. More detail on the other models we compared can be found in Section 1.6, and more detail on the model comparison process can be found above in Section 2.10.1.1.

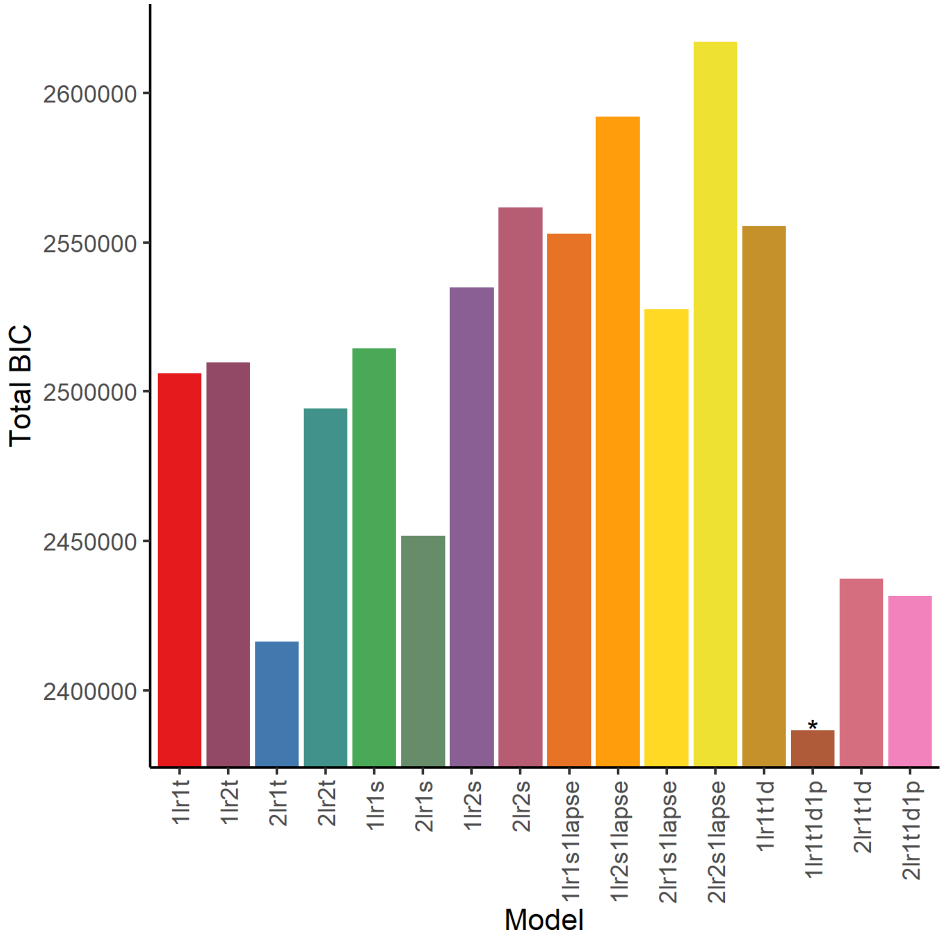

Supplementary Figure 16: Bar graph of BIC for each model after parameter estimation using maximum a-posteriori with separate priors for each group. The best fitting model is marked with an asterisk.

2.10.5.2 Learning rate

	F	num Df	den Df	Pr(>F)	ges
**pat_con**	76.240	1.00	3057.00	0	0.011
**study**	10.492	26.00	3057.00	0	0.037
**task**	9.605	3.89	11891.71	0	0.002
**pat_con:task**	17.756	3.89	11891.71	0	0.003
**study:task**	1.817	101.14	11891.71	0	0.009

Cohen's d: 0.23[0.2,0.27]

2.10.5.3 Inverse temperature

	F	num Df	den Df	Pr(>F)	ges
**pat_con**	1.070	1	3057	0.301	0.000
**study**	1.315	26	3057	0.131	0.002
**task**	1.098	1	3057	0.295	0.000
**pat_con:task**	1.069	1	3057	0.301	0.000

Cohen's d: 0.02[-0.01,0.05]

2.10.5.4 Decay

	F	num Df	den Df	Pr(>F)	ges
**pat_con**	128.395	1.000	3057.00	0	0.012
**study**	4.746	26.000	3057.00	0	0.011
**task**	74.890	3.896	11909.87	0	0.017
**pat_con:task**	9.622	3.896	11909.87	0	0.002
**study:task**	2.998	101.294	11909.87	0	0.018

Cohen's d: 0.19[0.16,0.22]

2.10.5.5 Perseverance

	F	num Df	den Df	Pr(>F)	ges
**pat_con**	3.595	1.000	3057.000	0.058	0.000
**study**	1.019	26.000	3057.000	0.437	0.002
**task**	0.426	1.158	3541.528	0.543	0.000
**pat_con:task**	1.397	1.158	3541.528	0.242	0.000
**study:task**	0.857	30.121	3541.528	0.689	0.006

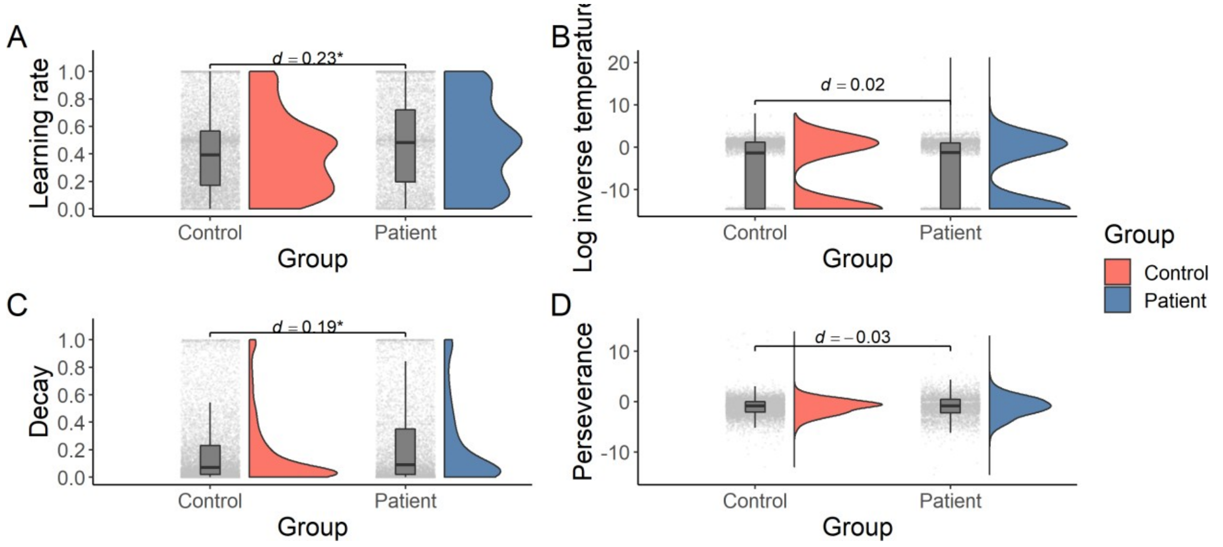

Supplementary Figure 17: Raincloud plots[44] showing the main effects of group on each parameter of the overall winning model fitted using a maximum a-posteriori approach with separate priors for each group. A) Learning rate. B) Log inverse temperature. C) Decay. D) Perseverance. On the left of each tick mark, a boxplot is displayed (showing median, first and third quartiles, with whiskers showing the largest value no more than 1.5*IQR from the third quartile, and the smallest value no more than 1.5*IQR from the first quartile), with the individual data points plotted in grey beneath it. On the right is a half violin plot, showing the density of the distribution. The Cohen's *d* effect size for group effects is displayed above to aid interpretation, and asterisks represent significant main effects of group in the ANOVA. Note that the y axis for perseverance is truncated to between -15 and +15, where the majority of the mass is.

2.10.5.6 Choice behaviour

Below, we show data that relates our choice bank data, generated from the original models, to data generated from this best-fitting model. We show that the trial-by-trial accuracy from our bank of choices (generated by feeding original parameters through each papers' best-fitting model for benchmarking tasks) tracks trial-by-trial accuracy for choices generated from the best-fitting model, shown averaged over different participants' choices for different tasks (Supplementary Figure 18).

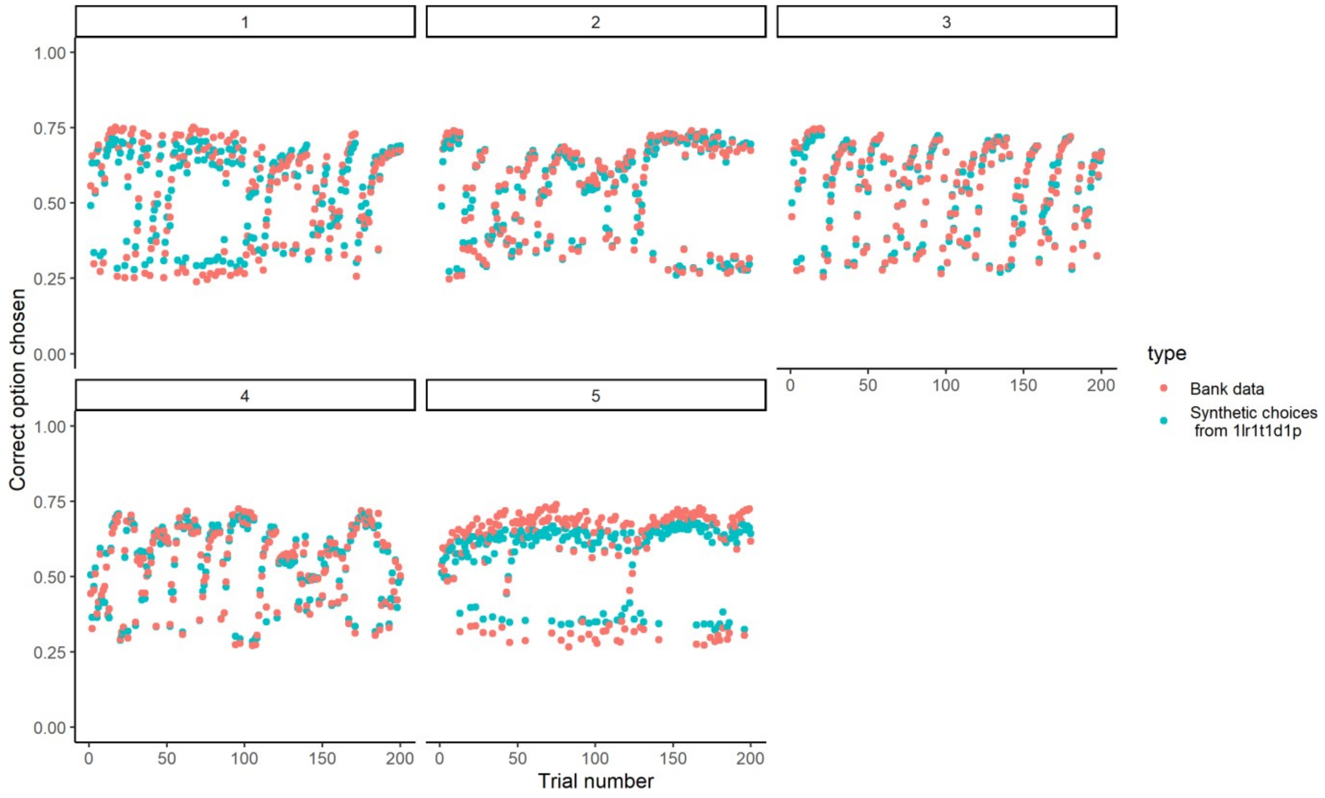

Supplementary Figure 18: Trial-by-trial accuracy (defined as choosing the option with the maximum value on each trial) across participants for each of the tasks, with both the choice bank data (from using original parameters and original models and feeding into the benchmarking tasks) and synthetic choices from the best-fitting model.

2.11 RECOVERABILITY ANALYSIS ON GENERATED TASKS

We tested the ability of the best-fitting models for each type of analysis to recover hypothetical parameters from the simulated tasks. 500 simulated 'agents' with a random combination of simulated parameters 'performed' the tasks in the paper using the overall best model, and the parameters were recovered using the relevant analytic approach (variational Bayes or maximum a-posteriori analysis). All correlations between generated and recovered parameters were positive and statistically different from 0 (Supplementary Figure 19). The simulated parameters were generated based on realistic distributions, informed by the estimates obtained in this paper. Thus, all learning rate parameters were generated using a $Beta(1,1)$ distribution, temperature parameters were generated using a $Gamma(5,1)$ distribution, sensitivity and bias parameters using a $Gamma(3,1)$ distribution, and lapse parameters using a $Gamma(0.5,1.5)$ distribution.

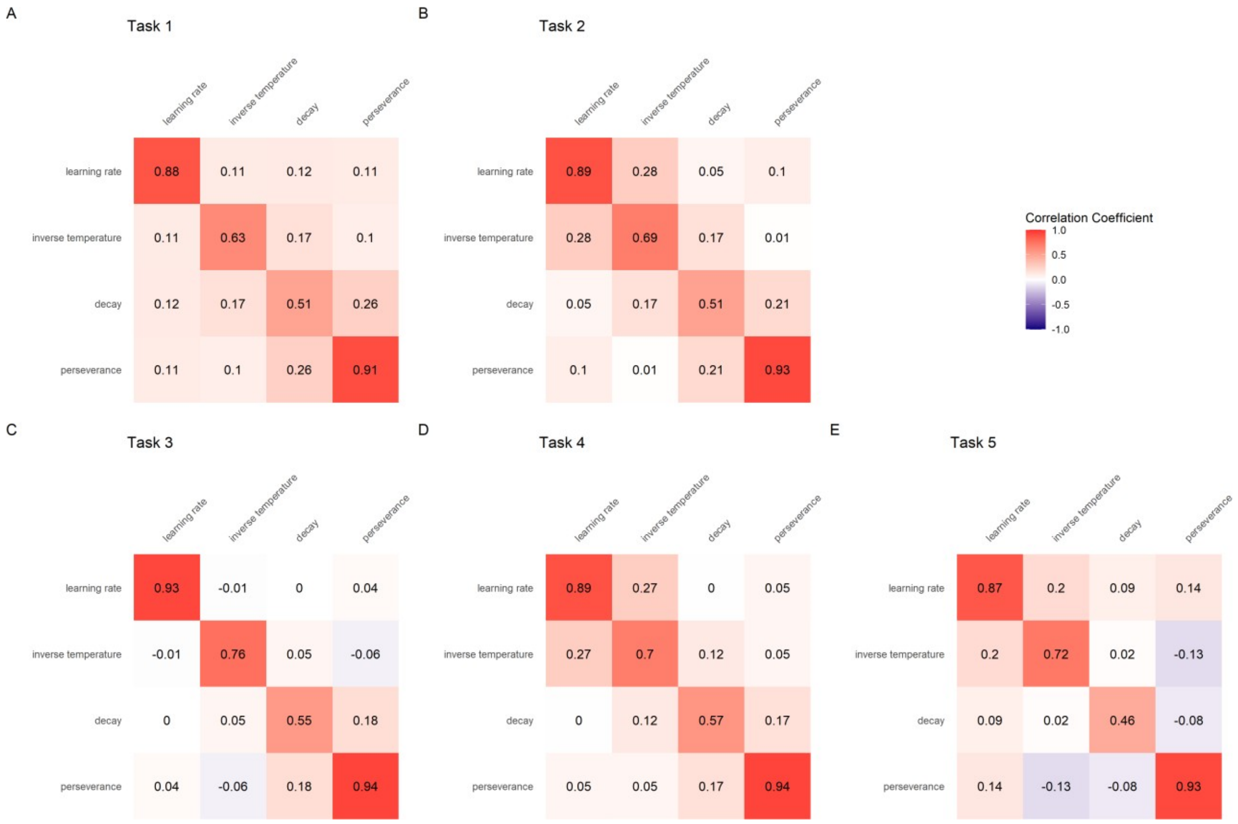

Supplementary Figure 19: Correlations between synthetic and estimated parameters for the overall winning model across all tasks estimated using variational Bayes, which had a single learning rate, an inverse temperature parameter, a decay parameter, and a perseverance parameter. The diagonal is the correlation between the generated and recovered parameters, and all off-diagonal squares are the correlations between two different recovered parameters. A) Task 1, B) Task 2, C) Task 3, D) Task 4. E) Task 5.

We also show for comparison the recovery for the second best models for variational Bayes estimation, for both estimation with a single prior (which was a model with a single learning rate and two inverse temperature parameters), and group-level priors (a model with two learning rates and one sensitivity parameter).

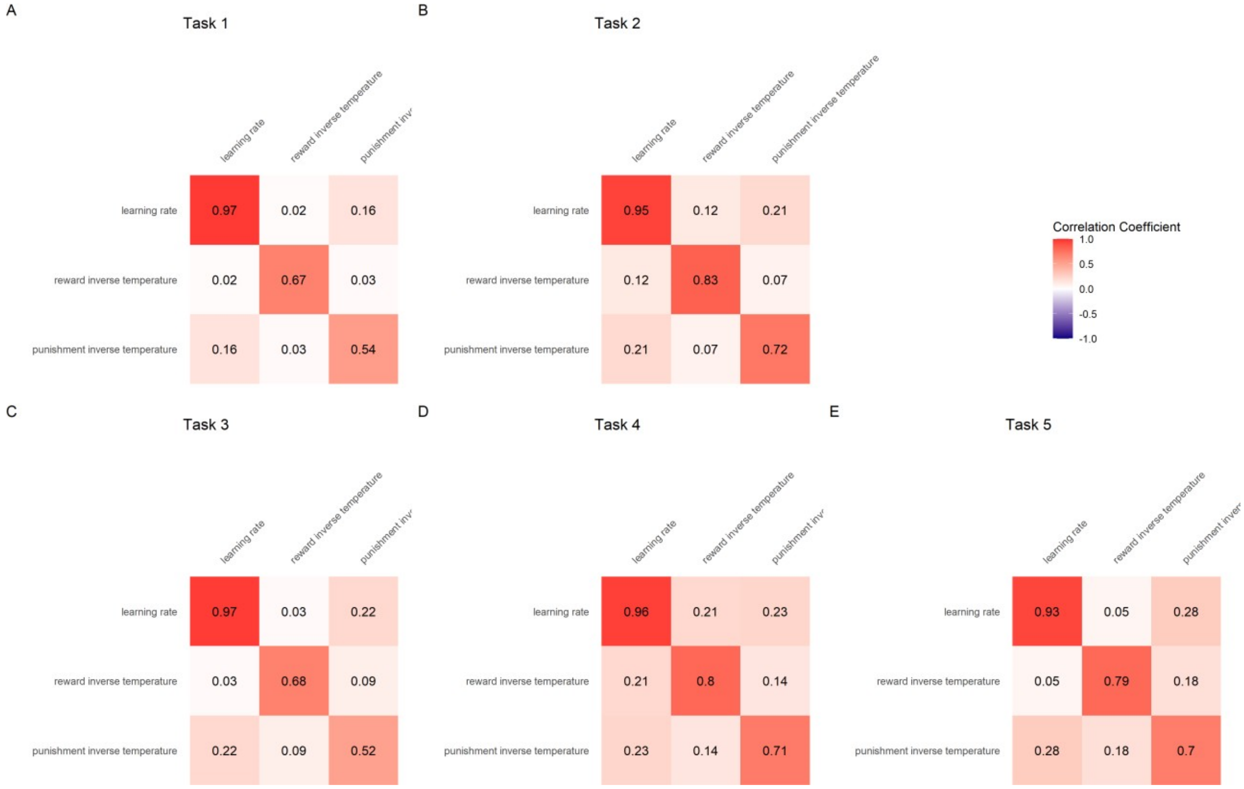

Supplementary Figure 20: Correlations between synthetic and estimated parameters for the second best model across all tasks estimated using variational Bayes with a single prior, which had a single learning rate and two inverse temperature parameters (for reward and punishments). The diagonal is the correlation between the generated and recovered parameters, and all off-diagonal squares are the correlations between two different recovered parameters. A) Task 1, B) Task 2, C) Task 3, D) Task 4. E) Task 5.

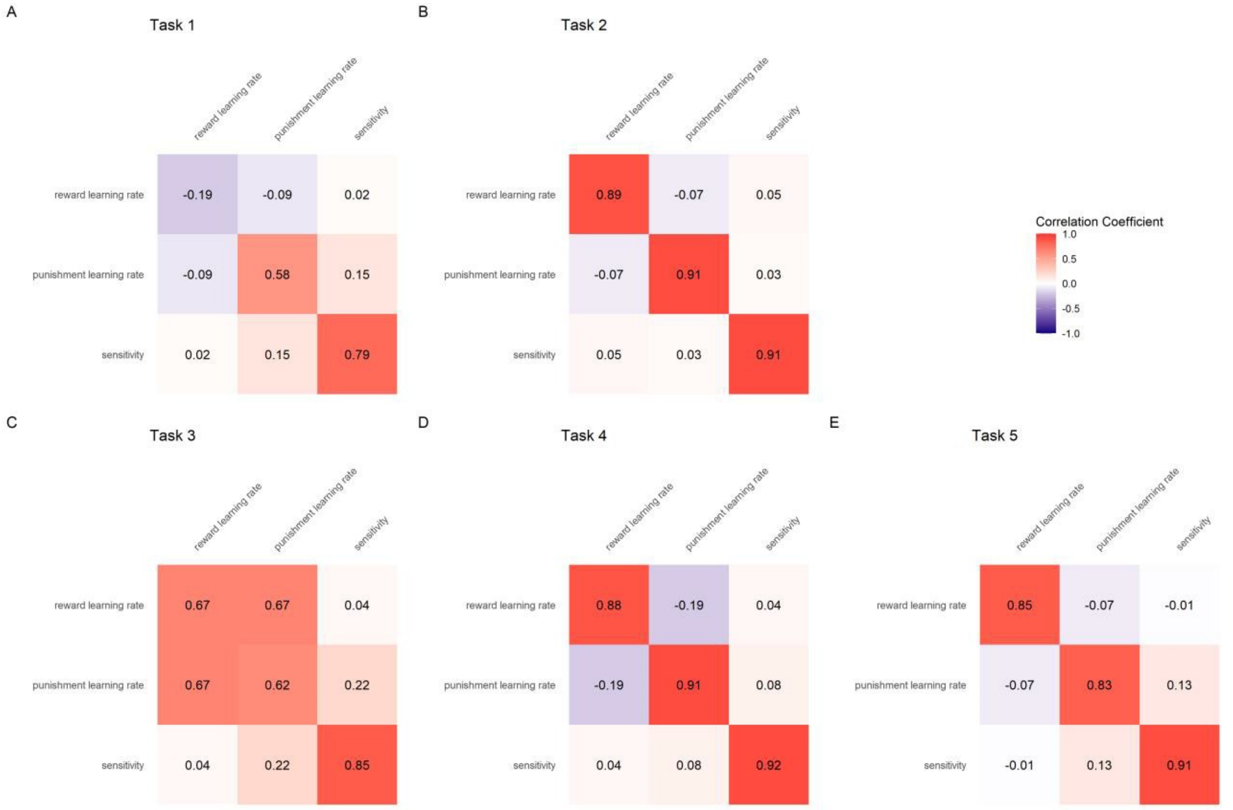

Supplementary Figure 21: Correlations between synthetic and estimated parameters for the second best model across all tasks estimated using variational Bayes with separate group-level priors, which had two learning rates (for rewards and punishments) and one sensitivity parameter. The diagonal is the correlation between the generated and recovered parameters, and all off-diagonal squares are the correlations between two different recovered parameters. A) Task 1, B) Task 2, C) Task 3, D) Task 4. E) Task 5.

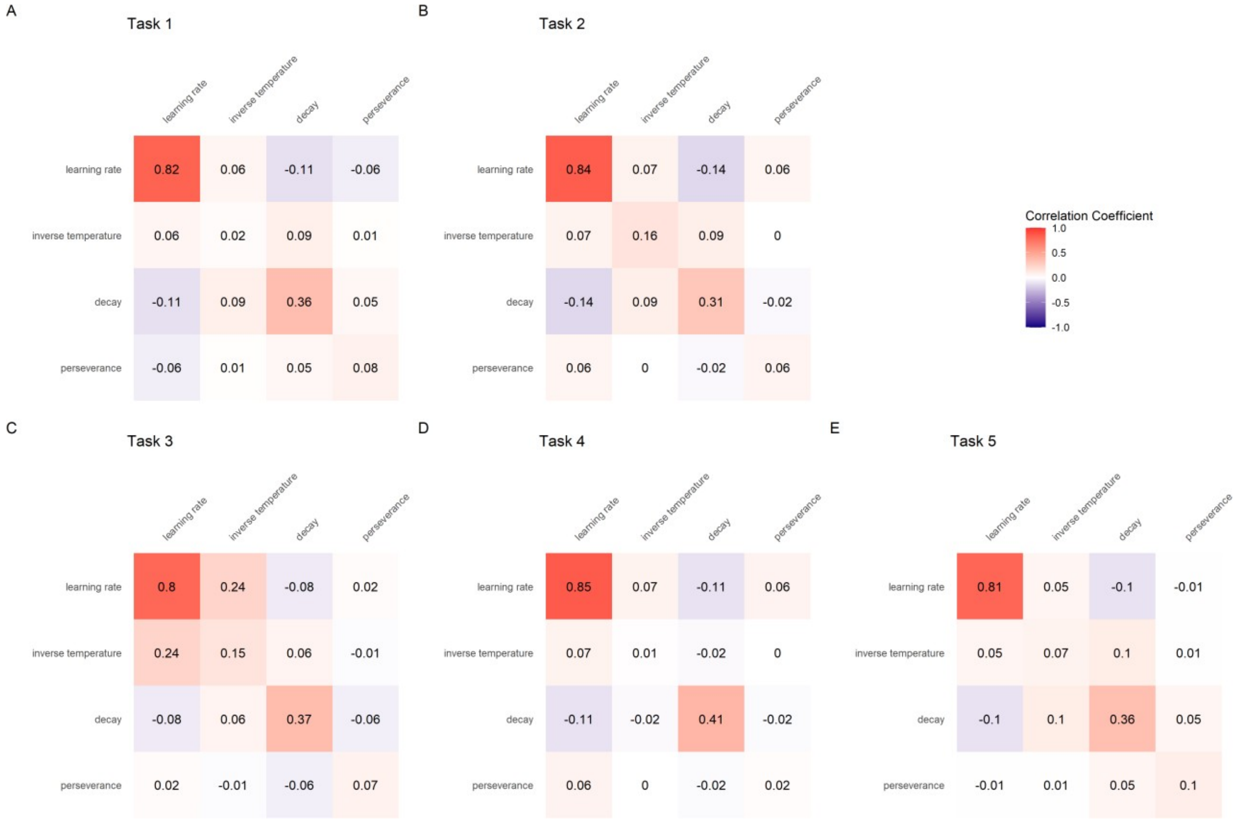

Supplementary Figure 22: Correlations between synthetic and estimated parameters for the overall winning model across all tasks estimated using maximum a-posteriori, which had one learning rate, one inverse temperature, one decay term, and one perseverance term. The diagonal is the correlation between the generated and recovered parameters, and all off-diagonal squares are the correlations between two different recovered parameters. A) Task 1, B) Task 2, C) Task 3, D) Task 4. E) Task 5.

We also include for comparison the model recovery using maximum a-posteriori for a model with two learning rates and one inverse temperature, which was significantly better.

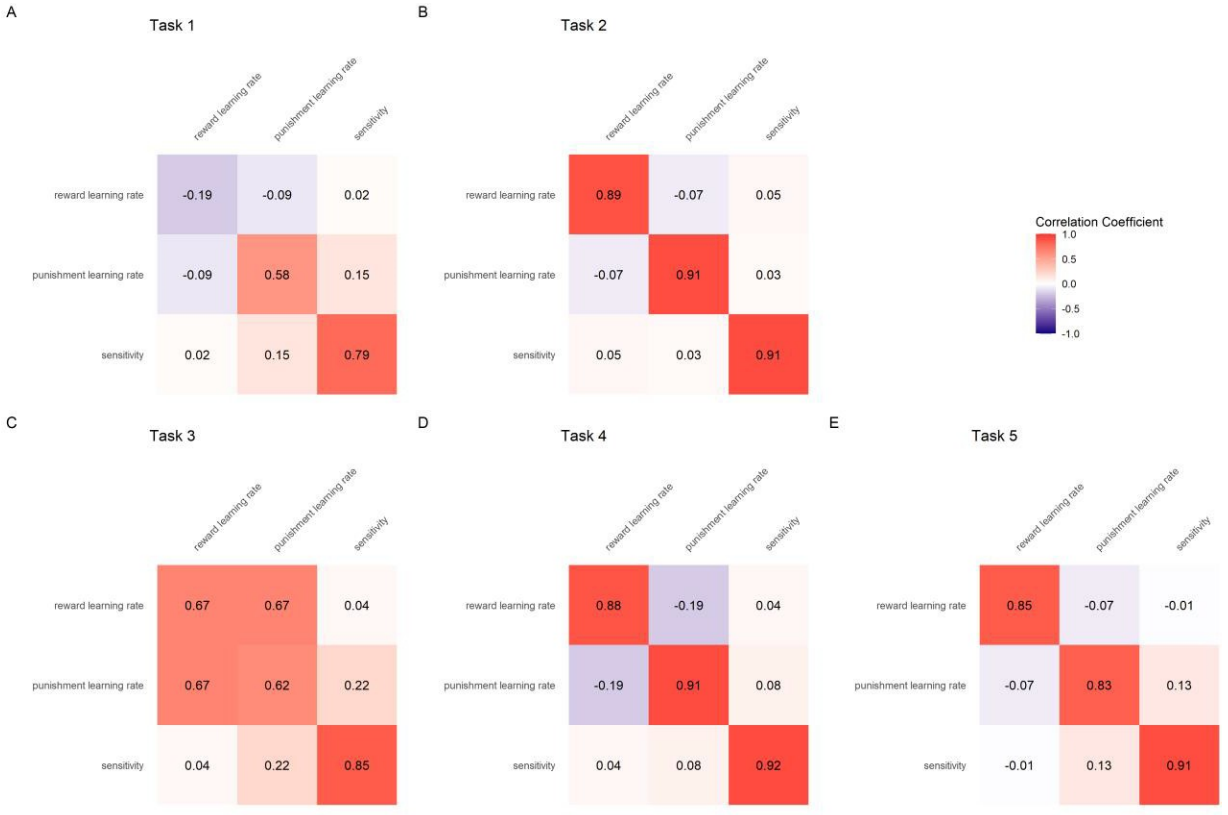

Supplementary Figure 23: Correlations between synthetic and estimated parameters for the second best model across all tasks estimated using variational Bayes with separate group-level priors, which had two learning rates (for rewards and punishments) and one sensitivity parameter. The diagonal is the correlation between the generated and recovered parameters, and all off-diagonal squares are the correlations between two different recovered parameters. A) Task 1, B) Task 2, C) Task 3, D) Task 4. E) Task 5.

3 SUPPLEMENTARY REFERENCES

1. Bramer, W. M., Rethlefsen, M. L., Kleijnen, J. & Franco, O. H. Optimal database combinations for literature searches in systematic reviews: a prospective exploratory study. *Syst. Rev.* **6**, 245 (2017).
2. Millner, A. J. *et al.* Suicidal thoughts and behaviors are associated with an increased decision-making bias for active responses to escape aversive states. *J. Abnorm. Psychol.* **128**, 106–118 (2019).
3. Balduzzi, S., Rücker, G. & Schwarzer, G. How to perform a meta-analysis with R: a practical tutorial. *Evid. Based Ment. Health* **22**, 153–160 (2019).

4. Wells, G. A. *et al. The Newcastle-Ottawa Scale (NOS) for assessing the quality of nonrandomised studies in meta-analyses.* (2000).

5. Halahakoon, D. C. *et al.* Reward-Processing Behavior in Depressed Participants Relative to Healthy Volunteers: A Systematic Review and Meta-analysis. *JAMA Psychiatry* **77**, 1286 (2020).

6. Higgins, J. P. T. Measuring inconsistency in meta-analyses. *BMJ* **327**, 557–560 (2003).

7. Dombrovski, A. Y. *et al.* Reward/Punishment Reversal Learning in Older Suicide Attempters. *Am. J. Psychiatry* **167**, 699–707 (2010).

8. Dombrovski, A. Y., Szanto, K., Clark, L., Reynolds, C. F. & Siegle, G. J. Reward Signals, Attempted Suicide, and Impulsivity in Late-Life Depression. *JAMA Psychiatry* **70**, 1020 (2013).

9. Huang, H., Thompson, W. & Paulus, M. P. Computational Dysfunctions in Anxiety: Failure to Differentiate Signal From Noise. *Biol. Psychiatry* **82**, 440–446 (2017).

10. Mukherjee, D., Filipowicz, A. L. S., Vo, K. D., Sattherwaite, T. & Kable, J. Reward and punishment reversal learning in major depressive disorder. *J. Abnorm. Psychol.* (2020) doi:10.31234/osf.io/aqgx3.

11. Dombrovski, A. Y. *et al.* Corticostriatothalamic reward prediction error signals and executive control in late-life depression. *Psychol. Med.* **45**, 1413–1424 (2015).

12. Ross, M. C., Lenow, J. K., Kilts, C. D. & Cisler, J. M. Altered neural encoding of prediction errors in assault-related posttraumatic stress disorder. *J. Psychiatr. Res.* **103**, 83–90 (2018).

13. Lamba, A., Frank, M. J. & FeldmanHall, O. Anxiety Impedes Adaptive Social Learning Under Uncertainty. *Psychol. Sci.* **31**, 592–603 (2020).

14. Kumar, P. *et al.* Impaired reward prediction error encoding and striatal-midbrain connectivity in depression. *Neuropsychopharmacology* **43**, 1581–1588 (2018).

15. Brown, V. M. *et al.* Associability-modulated loss learning is increased in posttraumatic stress disorder. *eLife* **7**, e30150 (2018).

16. Aylward, J. *et al.* Altered learning under uncertainty in unmedicated mood and anxiety disorders. *Nat. Hum. Behav.* **3**, 1116–1123 (2019).

17. Sutton, R.S; Barto, A. G. *Introduction to reinforcement learning*. vol. 135 (MIT Press, 1998).

18. Huys, Q. J., Pizzagalli, D. A., Bogdan, R. & Dayan, P. Mapping anhedonia onto reinforcement learning: a behavioural meta-analysis. *Biol. Mood Anxiety Disord.* **3**, 12 (2013).

19. Mkrtchian, A., Aylward, J., Dayan, P., Roiser, J. P. & Robinson, O. J. Modeling Avoidance in Mood and Anxiety Disorders Using Reinforcement Learning. *Biol. Psychiatry* **82**, 532–539 (2017).

20. Sugawara, M. & Katahira, K. Dissociation between asymmetric value updating and perseverance in human reinforcement learning. *Sci. Rep.* **11**, 3574 (2021).

21. Wilson, R. C. & Collins, A. G. Ten simple rules for the computational modeling of behavioral data. *eLife* **8**, e49547 (2019).

22. Vehtari, A. *et al. loo: Efficient leave-one-out cross-validation and WAIC for Bayesian models*. (2020).

23. Yao, Y., Vehtari, A., Simpson, D. & Gelman, A. Using stacking to average Bayesian predictive distributions. *Bayesian Anal.* (2017) doi:10.1214/17-BA1091.

24. Kucukelbir, A., Ranganath, R., Gelman, A. & Blei, D. M. Automatic Variational Inference in Stan. *ArXiv150603431 Stat* (2015).

25. Valton, V., Wise, T. & Robinson, O. J. *The Importance of Group Specification in Computational Modelling of Behaviour*. https://osf.io/p7n3h (2020) doi:10.31234/osf.io/p7n3h.

26. Moutoussis, M., Hopkins, A. K. & Dolan, R. J. Hypotheses About the Relationship of Cognition With Psychopathology Should be Tested by Embedding Them Into Empirical Priors. *Front. Psychol.* **9**, 2504 (2018).

27. Moher, D., Liberati, A., Tetzlaff, J., Altman, D. G., & The PRISMA Group. Preferred Reporting Items for Systematic Reviews and Meta-Analyses: The PRISMA Statement. *PLoS Med.* **6**, e1000097 (2009).

28. Blanco, N. J., Otto, A. R., Maddox, W. T., Beevers, C. G. & Love, B. C. The influence of depression symptoms on exploratory decision-making. *Cognition* **129**, 563–568 (2013).

29. Cavanagh, J. F., Bismark, A. W., Frank, M. J. & Allen, J. J. B. Multiple Dissociations Between Comorbid Depression and Anxiety on Reward and Punishment Processing: Evidence From Computationally Informed EEG. *Comput. Psychiatry Camb. Mass* **3**, 1–17 (2019).

30. Chase, H. W. *et al.* Approach and avoidance learning in patients with major depression and healthy controls: relation to anhedonia. *Psychol. Med.* **40**, 433 (2010).

31. Dombrovski, A. Y., Hallquist, M. N., Brown, V. M., Wilson, J. & Szanto, K. Value-Based Choice, Contingency Learning, and Suicidal Behavior in Mid- and Late-Life Depression. *Biol. Psychiatry* **85**, 506–516 (2019).

32. Frey, A.-L., Frank, M. J. & McCabe, C. Social reinforcement learning as a predictor of real-life experiences in individuals with high and low depressive symptomatology. *Psychol. Med.* 1–8 (2019) doi:10.1017/S0033291719003222.

33. Gagne, C., Zika, O., Dayan, P. & Bishop, S. J. Impaired adaptation of learning to contingency volatility in internalizing psychopathology. *eLife* **9**, e61387 (2020).

34. Gradin, V. B. *et al.* Expected value and prediction error abnormalities in depression and schizophrenia. *Brain* **134**, 1751–1764 (2011).

35. Khdour, H. Y. *et al.* Generalized Anxiety Disorder and Social Anxiety Disorder, but Not Panic Anxiety Disorder, Are Associated with Higher Sensitivity to Learning from Negative Feedback: Behavioral and Computational Investigation. *Front. Integr. Neurosci.* **10**, 20 (2016).

36. Kunisato, Y. *et al.* Effects of depression on reward-based decision making and variability of action in probabilistic learning. *J. Behav. Ther. Exp. Psychiatry* **43**, 1088–1094 (2012).

37. Liu, W.-H., Valton, V., Wang, L.-Z., Zhu, Y.-H. & Roiser, J. P. Association between habenula dysfunction and motivational symptoms in unmedicated major depressive disorder. *Soc. Cogn. Affect. Neurosci.* **12**, 1520–1533 (2017).

38. Myers, C. E. *et al.* Learning to Obtain Reward, but Not Avoid Punishment, Is Affected by Presence of PTSD Symptoms in Male Veterans: Empirical Data and Computational Model. *PLOS ONE* **8**, e72508 (2013).

39. Rupprechter, S., Stankevicius, A., Huys, Q. J. M., Steele, J. D. & Seriès, P. Major Depression Impairs the Use of Reward Values for Decision-Making. *Sci. Rep.* **8**, 13798 (2018).

40. Rupprechter, S. *et al.* Blunted medial prefrontal cortico-limbic reward-related effective connectivity and depression. *Brain* **143**, 1946–1956 (2020).

41. White, S. F. *et al.* Prediction Error Representation in Individuals With Generalized Anxiety Disorder During Passive Avoidance. *Am. J. Psychiatry* **174**, 110–117 (2017).

42. Sutton, A. J., Abrams, K. R., Jones, D. R., Sheldon, T. A. & Song, F. *Methods for Meta-analysis in Medical Research*. (John Wiley & Sons, Ltd).

43. Jeffreys, H. *The Theory of Probability*. (OUP Oxford, 1998).

44. Allen, M., Poggiali, D., Whitaker, K., Marshall, T. R. & Kievit, R. A. Raincloud plots: a multi-platform tool for robust data visualization. *Wellcome Open Res.* **4**, 63 (2019).

MOOSE Checklist for Meta-analyses of Observational Studies

Item No	Recommendation	Reported on Page No
Reporting of background should include		
1	Problem definition	5
2	Hypothesis statement	5,6,7
3	Description of study outcome(s)	7,8
4	Type of exposure or intervention used	5,6,7
5	Type of study designs used	5,6,7
6	Study population	5,6,7
Reporting of search strategy should include		
7	Qualifications of searchers (eg, librarians and investigators)	8
8	Search strategy, including time period included in the synthesis and key words	8, S3
9	Effort to include all available studies, including contact with authors	S3
10	Databases and registries searched	S3
11	Search software used, name and version, including special features used (eg, explosion)	S3
12	Use of hand searching (eg, reference lists of obtained articles)	S3
13	List of citations located and those excluded, including justification	OSF
14	Method of addressing articles published in languages other than English	S3
15	Method of handling abstracts and unpublished studies	S3
16	Description of any contact with authors	S3
Reporting of methods should include		
17	Description of relevance or appropriateness of studies assembled for assessing the hypothesis to be tested	7,8
18	Rationale for the selection and coding of data (eg, sound clinical principles or convenience)	7,8,,S3
19	Documentation of how data were classified and coded (eg, multiple raters, blinding and interrater reliability)	8,S3
20	Assessment of confounding (eg, comparability of cases and controls in studies where appropriate)	S27-S29
21	Assessment of study quality, including blinding of quality assessors, stratification or regression on possible predictors of study results	S4-S5
22	Assessment of heterogeneity	S5
23	Description of statistical methods (eg, complete description of fixed or random effects models, justification of whether the chosen models account for predictors of study results, dose-response models, or cumulative meta-analysis) in sufficient detail to be replicated	8-9, S4-S14

24	Provision of appropriate tables and graphics	Y
Reporting of results should include		
25	Graphic summarizing individual study estimates and overall estimate	Figures 2 and 3
26	Table giving descriptive information for each study included	S14-S19
27	Results of sensitivity testing (eg, subgroup analysis)	S27-S30
28	Indication of statistical uncertainty of findings	Figures and 10-11
Reporting of discussion should include		
29	Quantitative assessment of bias (eg, publication bias)	S21
30	Justification for exclusion (eg, exclusion of non-English language citations)	S1
31	Assessment of quality of included studies	S20
Reporting of conclusions should include		
32	Consideration of alternative explanations for observed results	14-16
33	Generalization of the conclusions (ie, appropriate for the data presented and within the domain of the literature review)	11-14
34	Guidelines for future research	14-16
35	Disclosure of funding source	22-23

From: Stroup DF, Berlin JA, Morton SC, et al, for the Meta-analysis Of Observational Studies in Epidemiology (MOOSE) Group. Meta-analysis of Observational Studies in Epidemiology. A Proposal for Reporting. *JAMA*. 2000;283(15):2008-2012. doi: 10.1001/jama.283.15.2008.

Original models and modifications

General principles

1. Reward and punishment learning rates were used to update learnt values in response to reward and punishment outcomes, respectively. Note that some original papers estimated learning rates for rewards and punishment from different blocks or tasks, but our benchmarking tasks (except the go-nogo task) included the potential for reward or punishment outcomes on every trial.
2. Reward/punishment inverse temperatures that had been estimated from different blocks or tasks were averaged. This is because it is not possible to determine whether a reward or punishment outcome will result on each trial until after the inverse temperature has been applied.
3. Bias terms that were specific to stimuli from the original task were removed. This is because these biases are assumed not to generalize to new stimuli in other tasks.
4. Any 'action bias' parameters were added to all relevant calculations for benchmarking tasks where both options involved making an action (e.g. choosing between different stimuli). This, in our opinion, is more true to the original models than removing these bias terms.
5. We used a softmax rather than a sigmoid equation in several places for ease. These are equivalent to a certain level of accuracy (>4 decimal places).

Notation

Parameter	Meaning
α	Learning rate
β	Inverse temperature (> is more deterministic)
τ	Temperature (> is more stochastic)
ξ	Lapse (> is more stochastic)
ρ	Outcome sensitivity
Q	Learnt value
δ	Prediction error (difference between expected and obtained outcomes)
n	Number of stimuli
t	Current trial
a	Action
η	Associability weight
κ	Associability value
γ	Decay

1 AYLWARD *ET AL.*, *NAT HUM BEHAV*. 3, 1116–1123 (2019).

The best-fitting model had six parameters: two learning rate parameters, for reward and punishment outcomes (α_{win} and α_{loss}), two sensitivity parameters (ρ_{win} and ρ_{loss}), a lapse parameter (ξ), and a decay parameter which governed the rate at which unchosen values decayed.

Two models were used for inference in this paper. We chose the simpler of the two models due to concerns about parameter trade-offs in the model with a decay parameter.

There was some fictive updating, in that the prediction error for the unchosen option was set to -1 * learnt value for that option.

$$P_{t(a_t)} = (1 - \xi) * \frac{exp(Q_{t(a_t)})}{\sum_{a=1}^{n} exp\ (Q_{t(a_t)})} + \frac{\xi}{n}$$

Where the learnt values are defined as:

$$Q_{t(a_t)} = Q_{t(a_t),win} - Q_{t(a_t),loss}$$

$$Q_{t+1(a_t),reward} = Q_{t(a_t),reward} + \alpha_{reward} * \delta_{t(a_t),reward}$$

$$Q_{t+1(a_t),punishment} = Q_{t(a_t),punishment} + \alpha_{punishment} * \delta_{t(a_t),punishment}$$

Where the prediction error (δ) for the chosen stimulus

$$\delta_{t(a_t),reward} = \rho_{reward} * reward_t - Q_{t(a_t),reward}$$

$$\delta_{t(a_t),punishment} = \rho_{punishment} * punishment_t - Q_{t(a_t),punishment}$$

For the unchosen stimulus

$$\delta_{t(a_t),reward} = -Q_{t(a_t),reward}$$

$$\delta_{t(a_t),punishment} = -Q_{t(a_t),punishment}$$

Note that t refers to the particular trial for which updating is occurring, and n refers to the number of stimuli. *Q* indicates a learnt value, and δ denotes prediction errors.

2 BLANCO, OTTO, MADDOX, BEEVERS, LOVE, *COGNITION*. 129, 563–568 (2013).

Uses two models, both more clearly defined in Knox et al. (2012) (**https://doi.org/10.3389/fpsyg.2011.00398**).

One is a naïve RL model, and the other is an ideal observer model, which is not based on the framework of reinforcement learning but optimal Bayesian inference so was not used.

The naïve RL model is equivalent to a Rescorla-Wagner model with a learning rate set at 1.

$$P_{t(a_t)} = \frac{exp(\beta * Q_{t(a_t)})}{\sum_{a=1}^{n} exp\ (\beta * Q_{t(a_t)})}$$

$$Q_{t+1(a_t)} = Q_{t(a_t)} + \delta_{t(a_t)}$$
$$\delta_t = outcome_t - Q_{t(a_t)}$$

3 BROWN *ET AL.*, *ELIFE*. 7, E30150 (2018).

The winning model in this paper included associability, such that learning rate was modulated on a trial-by-trial basis by an associability value (κ) for the stimulus that was chosen. An associability weight (η) was a free parameter estimated per participant, and controlled how much previous prediction errors updated the associability value. Note that associability weight was bounded at 0 and 1, and associability values were initialised at 1 and constrained to stay between 0.05 and 1. In their task, blocks where wins and punishment could occur were separate, so all parameters were estimated separately for reward and punishment outcomes. The model with an associability term did not win model comparison for rewards, only for punishment, so this term is not included for reward outcomes.

$$Q_{t+1(a_t),reward} = Q_{t(a_t),reward} + \alpha_{reward} * \delta_{t(a_t),reward}$$

$$Q_{t+1(a_t),punishment} = Q_{t(a_t),punishment} + \alpha_{punishment} * \kappa_{t(a_t),punishment} * \delta_{t(a_t),punishment}$$

Where

$$\delta_{t(a_t),reward} = \rho_{reward} * reward_t - Q_{t(a_t),reward}$$

$$\delta_{t(a_t),punishment} = \rho_{punishment} * punishment_t - Q_{t(a_t),punishment}$$

And

$$\kappa_{t+1(a_t),punishment} = (1 - \eta) * \kappa_{t(a_t),punishment} + \eta * |\delta_{t(a_t),punishment}|$$

And the unchosen option was decayed

$$Q_{t+1(a_t),reward} = \gamma_{reward} * Q_{t(a_t),reward}$$

$$Q_{t+1(a_t),punishment} = \gamma_{punishment} * Q_{t(a_t),punishment}$$

These values were then converted into probabilities of making a choice, with an inverse temperature (β) parameter included. However, this parameter was estimated separately prior to running the model including a sensitivity term, as these trade off. The β parameter for actual model estimation was set to the mean for each group, so we have done the same.

$$P_{t(a_t)} = \frac{exp(\beta * Q_{t(a_t)})}{\sum_{a=1}^{n} exp\ (\beta * Q_{t(a_t)})}$$

Some participants didn't have parameters for both valences, in which case we used the parameters for the valence they did have. To transfer these parameters into our tasks, specifically the ones in which

rewards and punishments could occur together, we used the learning rate that was appropriate given the outcome they had just received – reward learning rate for +1 outcomes, and punishment otherwise for the majority of benchmarking tasks, and reward learning rate for 0 outcomes for negative stimuli in go-nogo tasks.

In tasks that took a go-no-go format, we updated separate associability values for each choice per stimulus – go or no-go. Otherwise, we maintained separate associability values for each stimulus.

4 Cavanagh, Bismark, Frank, Allen, *Comput Psychiatr*. 3, 1–17 (2019).

In this model, there was a single Q value, and the learning rate depended on whether the feedback was positive or negative. There were therefore two separate learning rates for trials on which participants won, and when they did not.

$$Q_{t+1(a_t)} = Q_{t(a_t)} + \alpha * \delta_{t(a_t)}$$

Where

$$outcome_t = 1 \Longrightarrow \alpha = \alpha_{reward}$$

$$outcome_t = 0 \Longrightarrow \alpha = \alpha_{punishment}$$

And

$$\delta_{t(a_t)} = outcome_t - Q_{t(a_t)}$$

These learnt values were combined into a softmax.

$$P_{t(a_t)} = \frac{exp(Q_{t(a_t)} * \beta)}{\sum_{a=1}^{n} exp\ (Q_{t(a_t)} * \beta)}$$

As they had only reward outcomes and no-reward outcomes, their Q values were initialised at 0.5, halfway between the two. We modified this model to allow for it to be fit to our benchmarking tasks in several ways: we initialised Q values at 0, as we had punishments (outcomes of -1), and determined that the reward learning rate would be used for any outcomes that were 1 for all tasks other than go-nogo tasks, for which the reward learning rate was used for successfully obtaining reward or avoiding punishment.

5 Chase et al., Psychological Medicine. 40, 433 (2010).

This model is identical to the one from Cavanagh *et al.*, with the exception that temperature is used rather than inverse temperature.

$$P_{t(a_t)} = \frac{exp(\frac{Q_{t(a_t)}}{\tau})}{\sum_{a=1}^{n} exp\ (\frac{Q_{t(a_t)}}{\tau})}$$

6 Dombrovski *et al.*, *Psychol. Med.* 45, 1413–1424 (2015).

This model used the same model as Dombrovski *et al.* (2010). In this model, learning was as shown above, with the addition of a memory term.

$$Q_{t+1(a_t)} = memory * Q_{t(a_t)} + \alpha * \delta_{t(a_t)}$$

$$\delta_{t(a_t)} = outcome_t - Q_{t(a_t)}$$

Where

$$outcome_t > 0 \Longrightarrow \alpha = \alpha_{reward}$$

$$outcome_t < 0 \Longrightarrow \alpha = \alpha_{punishment}$$

Furthermore, this model had reciprocal updating for the unchosen stimulus (denoted a'), similarly to Aylward *et al.* (2019) but for the entire expected value rather than just the prediction error.

$$Q_{t(a'_t)} = -Q_{t(a_t)}$$

This was then converted into probability of making choices using a sigmoid:

$$P_{t(a_t)} = \frac{1}{1 + \exp((10 - \beta) * Q_{t(a_t)})}$$

As above, where outcomes were 1 in non go-nogo tasks, we used reward learning rates, and otherwise used punishment learning rates.

7 Dombrovski et al., American Journal of Psychiatry. 167, 699–707 (2010).

This model is described under Dombrovski *et al.* (2015).

8 Dombrovski, Hallquist, Brown, Wilson, Szanto, *Biological Psychiatry*. 85, 506–516 (2019).

$$Q_{t+1(a_t)} = Q_{t(a_t)} + \alpha * \delta_{t(a_t)}$$

Where

$$\delta_{t(a_t)} = outcome_t - Q_{t(a_t)}$$

And

$$outcome_t = 1 \Longrightarrow \alpha = \alpha_{reward}$$

$$outcome_t = 0 \Longrightarrow \alpha = \alpha_{punishment}$$

The unchosen stimulus was updated using a decay parameter.

$$Q_{t+1(a'_t)} = \lambda Q_{t(a'_t)}$$

This was converted into action probabilities using a softmax with a temperature parameter.

$$P_{t(a_t)} = \frac{exp(\frac{Q_{t(a_t)}}{\tau})}{\sum_{a=1}^{n} exp\,(\frac{Q_{t(a_t)}}{\tau})}$$

9 Dombrovski, Szanto, Clark, Reynolds, Siegle, *JAMA Psychiatry*. 70, 1020 (2013).

This model is described under Dombrovski *et al.* (2015).

10 Frey, Frank, McCabe, *Psychol. Med.*, 1–8 (2019).

Note that there was a social and non-social task reported in this paper – we used only parameters from the non-social task, as none of our benchmarking tasks included a social element.

$$Q_{t+1(a_t)} = Q_{t(a_t)} + \alpha * \delta_{t(a_t)}$$

Where, as usual,

$$\delta_{t(a_t)} = outcome_t - Q_{t(a_t)}$$

However, note that the value of the outcome was determined by a parameter known as 'choice valuation', such that rewards received the value (1-choice_valuation), punishments were valued at (-1*choice_valuation), and neutral outcomes were valued at the midpoint between these (1-choice_valuation)-((1-choice_valuation)-(-1*choice_valuation))/2.

Subsequently, a softmax was used to transform learnt values into probabilities of choices:

$$P_{t(a_t)} = \frac{exp(\frac{Q_{t(a_t)} + c_{t(a_t)} * \varphi}{\tau})}{\sum_{a=1}^{n} exp\,(\frac{Q_{t(a_t)} + c_{t(a_t)} * \varphi}{\tau})}$$

This is similar to the softmax as shown above, with an additional term - $c_{t(a_t)} * \varphi$. $c_{t(a_t)}$ is an indicator variable which is 1 if the stimulus chosen on the previous trial was that action. So, to calculate the probability that stimulus A will be chosen, $c_{t(a_t)}$ is 1 if A was chosen on the previous trial, and otherwise is 0. φ is a free parameter representing how likely participants are to repeat their choices, or how 'sticky' their behaviour is. Note that the winning model did not contain any decay terms, though these are displayed in the illustrative equations in the supplement.

We initialised all Q values at 0.

11 Gagne, Zika, Dayan, Bishop, *eLife*. 9, e61387 (2020).

For the winning model, probabilities of outcomes were updated using the following:

$$p_{t(a_t)} = p_{t-1(a_t)} + \alpha * \delta_{t-1(a_t)}$$

Where

$$\delta_{t-1(a_t)} = outcome_{t-1} - p_{t-1(a_t)}$$

And value was calculated as follows

$$v_{t(a_t)} = \lambda[p_t - (1 - p_t)] + (1 - \lambda) * [M1_t - M2_t]^r$$

Where M1 is the magnitude of one stimulus, and M2 is the magnitude of the other. For our tasks, magnitude was not manipulated, so we set these to 1. R is a scaling parameter designed to capture the nonlinearity of perceived differences in magnitude. Subsequently, a choice kernel was updated, which governs the possibility that participants tend to repeat choices (or not repeat choices) independently of the outcomes they receive, with η acting as an update rate for how many previous choices are included in the value of the choice kernel:

$$k_{t(a_t)} = k_{t-1(a_t)} + \eta * (C_{t-1} - k_{t-1})$$

And actions were selected using a softmax

$$P_{t(a_t)} = \frac{1}{1 + \exp(-1 * (\beta * v_t + \omega_k\ [k_t - (1 - k_t)]))}$$

In these models, all parameters were broken up into components (for volatile vs. stable blocks, and for reward vs. punishment tasks, and the interaction).

Parameters were broken up into components, as follows:

$$\alpha = logistic(\alpha_{baseline} +$$

$$\alpha_{reward-aversive} * \theta_{reward-aversive} +$$

$$\alpha_{volatile-stable} * \theta_{volatile-stable} +$$

$$\alpha_{good-bad} * \theta_{good-bad} +$$

$$\alpha_{(reward-aversive)*(volatile-stable)} * \theta_{(reward-aversive)*(volatile-stable)} +$$

$$\alpha_{(reward-aversive)*(good-bad)} * \theta_{(reward-aversive)*(good-bad)} +$$

$$\alpha_{(good-bad)*(volatile-stable)} * \theta_{(good-bad)*(volatile-stable)})$$

Where θ indicated an indicator variable, which was 1 or -1 (e.g. $\theta_{volatile-stable}$ was 1 in the volatile block, and -1 in the stable block). The logistic transform was used to constrain the learning rate between 0 and 1, and a logarithmic transform was used for other parameters that constrained to be positive. Here, good-bad indicates learning rate shifts after positive vs. negative feedback. As all our tasks had volatile outcomes, we used learning rates including the volatile component. For the majority of our benchmarking tasks, in which there were no separate blocks for reward and punishment and these outcomes could both occur on any trial, so we set this component to 0, as follows:

$$\alpha_{win} = logistic(\alpha_{baseline} + \alpha_{volatile-stable} + \alpha_{good-bad} + \alpha_{(good-bad)*(volatile-stable)}$$

$$\alpha_{loss} = logistic(\alpha_{baseline} + \alpha_{volatile-stable} - \alpha_{good-bad} - \alpha_{(good-bad)*(volatile-stable)}$$

However, in our go-no-go task, there were stimuli that could either reward or have a neutral outcome, for which we used learning rates including all components, $\theta_{reward-aversive}$ set to 1, and stimuli which could either have a neutral outcome or result in a punishment, for which we used learning rates including all components with $\theta_{reward-aversive}$ set to -1. Notably, in this paper r and ω_k had only two components: baseline and a component for reward-aversive task versions, so we used only the transformed baselines. The update rate for the choice kernel (η) had only a baseline component.

12 Gradin *et al.*, *Brain*. 134, 1751–1764 (2011).

This paper used a SARSA model, a type of temporal difference model which incorporates the subsequent state the participant will be in and the value they expect to obtain from that state, in which the prediction error is defined based not only on the current state and reward obtained, but the next state.

$$Q_{t+1(a_t)} = Q_{t(a_t)} + \alpha * \delta_{t(a_t)}$$

$$\delta_t = outcome_t + \gamma * Q_{t(a_{t+1},s_{t+1})} - Q_{t(a_t,s_t)}$$

The γ parameter, which governs the discounting of future outcomes was set at 1. Q values were initialised at 0. Learnt values were then converted to probabilities using a softmax with inverse temperature.

$$P_{t(a_t)} = \frac{exp(Q_{t(a_t)} * \beta)}{\sum_{a=1}^{n} exp\ (Q_{t(a_t)} * \beta)}$$

As in the original paper, for the majority of our benchmarking tasks we assumed the next state was the same as the current one (although note that for go-nogo tasks the next stimulus presented was unpredictable), so we simply allowed the Q value for the next state to be the one for the current state, assuming the policy of choosing the action with the highest Q value was followed (max $Q_{t(a_t)}$).

13 Huang, Thompson, Paulus, *Biological Psychiatry*. 82, 440–446 (2017).

Here, values were updated using a 'VMax' rule, such that when the stimulus with the highest value changes the learning rate is adjusted.

$$Q_{t+1(a_t)} = Q_{t(a_t)} + \alpha * \delta_{t(a_t)}$$

Where

$$\delta_{t(a_t)} = outcome_t - Q_{t(a_t)}$$

And

$$argmax(Q_t) == argmax(Q_t) \Longrightarrow \alpha = \alpha_0$$

$$argmax(Q_t) =/= argmax(Q_t) \Longrightarrow \alpha = \alpha_0 + \alpha_d$$

These learnt values were then entered into a softmax with an inverse temperature parameter:

$$P_{t(a_t)} = \frac{exp(Q_{t(a_t)} * \beta)}{\sum_{a=1}^{n} exp\ (Q_{t(a_t)} * \beta)}$$

For trial 1, we set the learning rate as α_0.

14 Huys, Pizzagalli, Bogdan, Dayan, *Biology of Mood & Anxiety Disorders*. 3, 12 (2013).

The model used in this paper was a 'belief' model, as the task involved participants responding to identify a stimulus (as either a 'long' or a 'short' stimulus), where one of the two stimuli was more rewarded than another. This model included a form of Q learning, where the values of each action for each stimulus were updated as:

$$Q_{t+1(a_t,s_t)} = Q_{t(a_t,s_t)} + \alpha * \delta_{t(a_t,s_t)}$$

$$\delta_{t(a_t,s_t)} = \rho * outcome_t - Q_{t(a_t,s_t)}$$

And these Q values were combined into weights

$$weight_{t(a_t,s_t)} = \gamma * I_{t(a_t,s_t)} + \zeta * Q_{t(a_t,s_t)} + (1 - \zeta) * Q_{t(a_t,s'_t)}$$

Where $I_{t(a_t,s_t)}$ was 1 if the action was the instructed one for the stimulus, and γ defined participants' ability to follow the instructions. We set $I_{t(a_t,s_t)}$ to 0, as there were no instructions in our benchmarking tasks. ζ governed the weight given to the chosen and unchosen stimulus values, and in this paper this represented the belief they had that they had made the correct choice. To simulate choices on our

benchmarking tasks, we used the belief parameter and Q values for the two options to calculate the weight – so, between the two stimuli in the majority of the tasks, and between go and no-go actions in go-nogo tasks.

These weights were used to determine action probabilities using a sigmoid:

$$P_{t(a_t)} = \frac{1}{1 + \exp(-1 * (Q_{t(a_t)} - Q_{t(a'_t)}))}$$

15 Khdour *et al.*, *Front Integr Neurosci*. 10, 20 (2016).

Whilst actor-critic models were fit to the data in this paper, the best fitting model was a simple Q-learning model.

$$Q_{t+1(a_t)} = Q_{t(a_t)} + \alpha * \delta_{t(a_t)}$$

Where

$$\delta_{t(a_t)} = outcome_t - Q_{t(a_t)}$$

This was then converted into choices as

$$P_{t(a_t)} = \frac{1}{1 + \exp(-\beta(Q_{t(a_t)} - Q_{t(a'_t)}))}$$

Where a' is the other (unchosen) option. Note that when generating choices for the benchmarking tasks we used a softmax rather than a sigmoid, but these equations are reducible to each other when there are only two possible choices.

16 Kumar *et al.*, *Neuropsychopharmacology*. 43, 1581–1588 (2018).

This paper uses Q-learning with a temperature parameter.

$$Q_{t+1(a_t)} = Q_{t(a_t)} + \alpha * \delta_{t(a_t)}$$

$$\delta_{t(a_t)} = outcome_t - Q_{t(a_t)}$$

$$P_{t(a_t)} = \frac{exp(\frac{Q_{t(a_t)}}{\tau})}{\sum_{a=1}^{n} exp(\frac{Q_{t(a_t)}}{\tau})}$$

Notably, they fit separate learning rates and temperatures to the reward and punishment conditions. Many of the stimuli in our benchmarking tasks could receive rewards, punishments or neither; in these tasks we used the reward learning rate if participants received a reward, but otherwise the punishment learning rate; in the go-no-go task, where stimuli were either associated with reward or with punishment, we used the reward learning rate when they received the best outcome possible: rewards for rewarding stimuli, and omissions for punishing stimuli; and the punishment learning rate for omissions for rewarding stimuli and punishment for punishing stimuli. We used the average of the temperature parameters that they had estimated for the reward and punishment conditinos, as this parameter occurs in the model prior to a choice being made and an outcome being received.

17 Kunisato et al., Journal of Behavior Therapy and Experimental Psychiatry. 43, 1088–1094 (2012).

This model is very similar to some of the above, except that the value of the prediction error (whether it is over 0 or under 0) determines which learning rate is used.

$$Q_{t+1(a_t)} = memory * Q_{t(a_t)} + \alpha * \delta_{t(a_t)}$$

Where

$$\delta_t > 0 \Longrightarrow \alpha = \alpha_{win}$$

$$\delta_t < 0 \Longrightarrow \alpha = \alpha_{loss}$$

A softmax was used to convert these learnt values into action probabilities, including a temperature parameter.

$$P_{t(a_t)} = \frac{exp(\frac{Q_{t(a_t)}}{\tau})}{\sum_{a=1}^{n} exp\,(\frac{Q_{t(a_t)}}{\tau})}$$

18 Lamba, Frank, FeldmanHall, *Psychol Sci*. 31, 592–603 (2020).

This paper used a dynamic Bayesian reinforcement learning model for two conditions – a social and non-social condition. We used only parameters from the non-social condition as none of our benchmarking tasks involved a social element.

In this dynamic model, participants kept track of whether a slot machine was worth playing. This belief was approximated by a Beta distribution, with:

$$\mu_{t(a_t)} = (\frac{alpha}{alpha + beta})$$

$$\sigma^2{}_{t(a_t)} = (\frac{alpha * beta}{(alpha + beta)^2 * (alpha + beta + 1)})$$

Each reward incremented alpha by 1, and each punishment incremented beta by 1. In our benchmarking tasks, for stimuli which could receive outcomes of +1, 0 or -1, we incremented alpha for outcomes of +1 only; for the go-nogo task in which different stimuli could either receive +1 or 0; or 0 or -1, we incremented alpha for +1 in the first case and 0 (omission of punishment) in the second case, and beta for 0 in the first case (omission of reward) and -1 in the second case.

These parameters were converted to probabilities using a form of softmax with an inverse temperature:

$$P_{t(a_t)} = \frac{\exp(\beta * \mu_{t(a_t)})}{\exp(\beta * \mu_{t(a_t)}) + \exp(\beta * \psi)}$$

Where ψ is a bias term that governed tendency to invest or play a slot machine. If the subject was optimal the bias would be 0.5, which is the value we used for generation of choices in a benchmarking task as bias terms are likely to be specific to a task context. The probability of taking the other action was calculated as

$$P_{t(a'_t)} = 1 - P_{t(a_t)}$$

Entropy (H_t) and change in entropy (ΔH_t) were also calculated, in order to keep track of the probability of a change-point.

$$H_t = -[P_{t(a_t)} \times log_2(P_{t(a_t)}) - P_{t(a'_t)} \times log_2(P_{t(a'_t)})]$$

$$\Delta H_t = H_t - H_{t-1}$$

These parameters were used to inform the decay of both alpha and beta, such that higher decay meant more forgetting of previous outcomes, which would be optimal just after a change-point.

$$logit(\gamma_{pos}) = \gamma 0_{pos} + \gamma 1_{pos} \cdot \Delta H_t$$

$$logit(\gamma_{neg}) = \gamma 0_{neg} + \gamma 1_{neg} \cdot \Delta H_t$$

$$\alpha_{t+1} = a_t \cdot \gamma_{pos}$$

$$\beta_{t+1} = \beta_t \cdot \gamma_{neg}$$

γ_0 parameters correspond to the overall decay of previous outcomes, and γ_1 captures the extent to which decay is scaled by changes in entropy (ΔH).

19 Liu, Valton, Wang, Zhu, Roiser, *Social Cognitive and Affective Neuroscience*. 12, 1520–1533 (2017).

Standard Q-learning models were used in this paper. Note that one temperature was recorded as being 0.000, which we edited to 0.001, to avoid the intractability of dividing by 0.

$$Q_{t+1(a_t)} = Q_{t(a_t)} + \alpha * \delta_{t(a_t)}$$

$$\delta_{t(a_t)} = outcome_t - Q_{t(a_t)}$$

$$P_{t(a_t)} = \frac{exp(\frac{Q_{t(a_t)}}{\tau})}{\sum_{a=1}^{n} exp\,(\frac{Q_{t(a_t)}}{\tau})}$$

20 MILLNER *ET AL.*, *JOURNAL OF ABNORMAL PSYCHOLOGY*. 128, 106–118 (2019).

This model was a combined reinforcement learning model with a drift diffusion model (DDM). Q learning was used to update learnt values:

$$Q_{t+1(a_t)} = Q_{t(a_t)} + \alpha * \delta_{t(a_t)}$$

$$\delta_{t(a_t)} = outcome_t - Q_{t(a_t)}$$

These learnt values determined the drift rate μ_t

$$\mu_t = \beta_0 + \beta_1[Q_{t(a_t)} - Q_{t(a'_t)}]$$

Note that here the two available actions were go and nogo, and β_0 reflected a constant go bias, and β_1 represents a go bias shared across responses. The drift diffusion process was then calculated as the Wiener first-passage time (WFPT), parameterised by the non-decision time *T*, the boundary separation parameter ω, the starting point *z*, and the drift rate μ_t.

$$c, t \sim WFPT(T, \omega, z, w)$$

Note that they parameterised the starting point as

$$w = \frac{z}{\omega}$$

Which caused it to vary between 0 and 1.

They estimated the starting point separately for their 'escape' and 'avoid' conditions – we took the mean of these.

We were not interested in the reaction time *t* estimated, so we input the reaction time as 10s, to ensure that the majority of the cumulative distribution would be considered.

21 MKRTCHIAN, AYLWARD, DAYAN, ROISER, ROBINSON, *BIOLOGICAL PSYCHIATRY*. 82, 532–539 (2017).

$$Q_{t+1(a_t,s_t)} = Q_{t(a_t,s_t)} + \alpha * \delta_{t(a_t,s_t)}$$

$$V_{t+1(s_t)} = V_{t(s_t)} + \alpha * \delta_{t(s_t)}$$

$$\delta_{t(a_t,s_t)} = \rho * outcome_t - Q_{t(a_t,s_t)}$$

$$\delta_{t(a_t)} = \rho * outcome_t - V_{t(s_t)}$$

Note that values were not updated separately for different actions – therefore, in the majority of our benchmarking tasks, the value was updated based on the outcome received, and didn't differ between stimuli (for all reversal learning tasks and random walk tasks), and differed between stimuli only on go-no-go tasks.

Subsequently, these learnt values were converted into action weights:

$$weight_{t(a_t)} = Q_{t(a_t,s_t)} + ActionBias + PavlovianBias * V_{t(s_t)}$$

$$P_{t(a_t)} = (1 - \xi) * \frac{exp(weight_{t(a_t)})}{\sum_{a=1}^{n} exp\,(weight_{t(a_t)})} + \frac{\xi}{n}$$

Where

$$stimulus = rewarding \Longrightarrow \alpha = \alpha_{reward};\, \rho = \rho_{reward}; PavlovianBias = approach$$

$$stimulus = punishing \Longrightarrow \alpha = \alpha_{punishment};\;\; \rho = \rho_{punishment}; PavlovianBias = avoid$$

Additionally, in the majority of our benchmarking tasks participants had to make a choice between two stimuli rather than to go or not go – in these cases, action weights were calculated including the 'ActionBias' parameter for all choices. For benchmarking tasks where choices could result in reward or punishment, the bias used depended on the value of $V_{t(s_t)}$ – if positive, the approach bias was used, if negative, the avoid bias was used. Similarly, if the outcome was greater than 0, we used the reward learning rate, otherwise, we used the punishment learning rate.

22 Moutoussis *et al.*, *PLOS ONE*. 13, e0201451 (2018).

The model used in this paper is very similar to Mkrtchian et al.

$$Q_{t+1(a_t,s_t)} = Q_{t(a_t,s_t)} + \alpha * \delta_{t(a_t,s_t)}$$

$$V_{t+1(s_t)} = V_{t(s_t)} + \alpha * \delta_{t(s_t)}$$

$$\delta_{t(a_t,s_t)} = \rho * outcome_t - Q_{t(a_t,s_t)}$$

$$\delta_{t(a_t)} = \rho * outcome_t - V_{t(s_t)}$$

Here, however, the bias terms were only used if the state value was greater than 0:

$$weight_{t(a_t)} = Q_{t(a_t)} + ActionBias + PavlovianBias * {V_{t(s_t)}}_t$$

$${V_{t(s_t)}}_t > 0 \Longrightarrow PavlovianBias = TRUE; ActionBias = TRUE$$

$${V_{t(s_t)}}_t > 0 \Longrightarrow PavlovianBias = FALSE;\; ActionBias = TRUE$$

Additionally, the action had to be 'go' for the ActionBias to be used.

$$P_{t(a_t)} = (1 - \xi) * \frac{exp(weight_{t(a_t)})}{\sum_{a=1}^{n} exp\,(weight_{t(a_t)})} + \frac{\xi}{n}$$

Again, as above, we assumed that the ActionBias was true for all tasks in which there was no 'no-go' option. The sensitivity parameter depended on the value of the outcome – if positive, the reward sensitivity was used, otherwise the punishment sensitivity was used.

23 Mukherjee, Filipowicz, Vo, Sattherwaite, Kable, *Journal of Abnormal Psychology* (2020).

In this paper, a form of Q learning was used for both actions and fractals.

$$Q_{t+1(a_t)} = Q_{t(a_t)} + \delta_{t(a_t)}$$

$$Q_{t+1(f_t)} = Q_{t(f_t)} + \alpha * \delta_{t(f_t)}$$

The log odds that the participant would choose a fractal were then calculated as:

$$V_t = \beta_f[Q_{t(f_1)} - Q_{t(f_2)}] + \beta_a[Q_{t(a=f_1)} - Q_{t(a=f_2)}] + \rho_f + \rho_a + \pi_f + \pi_a$$

Which was then converted into probabilities

$$P_{t(a_t)} = \frac{1}{1 + exp(-V_t)}$$

This model was fit separately to reward and punishment conditions. For benchmarking tasks where stimuli could have any outcome (reward, punishment, or neutral), we took the mean of the reward and punishment perseveration terms, and the mean of the reward and punishment β_f and β_a terms. We used the positive learning rate if the outcome was positive, and otherwise we used the negative learning rate. We set all bias terms (indicating preference for a particular stimulus) to 0, as they are unlikely to generalise to entirely new hypothetical stimuli. We only added the perseveration bias terms if the choice was one that had been repeated on the previous trial. Notably there was nothing to distinguish fractal from action learning; nevertheless, both terms were included.

24 Myers *et al.*, *PLOS ONE*. 8, e72508 (2013).

This model is very similar to that used by Kunisato et al. or Frey et al., with the exception that the value of neutral outcomes (0, vs. +1 or -1) was estimated by a free parameter, *r0*.

$$Q_{t+1(a_t)} = Q_{t(a_t)} + \alpha * \delta_{t(a_t)}$$

Where

$$\delta_t > 0 \Longrightarrow \alpha = \alpha_{win}$$

$$\delta_t < 0 \Longrightarrow \alpha = \alpha_{loss}$$

A softmax was used to convert these learnt values into action probabilities, including a temperature parameter.

$$P_{t(a_t)} = \frac{exp(\frac{Q_{t(a_t)}}{\tau})}{\sum_{a=1}^{n} exp\,(\frac{Q_{t(a_t)}}{\tau})}$$

25 Rupprechter *et al.*, *Brain*. 143, 1946–1956 (2020).

$$Q_{t+1(a_t)} = Q_{t(a_t)} + \alpha * \delta_{t(a_t)}$$

$$\delta_{t(a_t)} = outcome_t - Q_{t(a_t)}$$

These values were converted to choice probabilities using a sigmoid:

$$P_{t(a_t)} = \frac{1}{1 + exp(-\beta * (Q_{t(a_t)} - Q_{t(a'_t)}))}$$

In this paper there were two separate learning rates for different conditions - where participants were permitted to make a choice, or where they just had to observe an outcome. We chose to only use the learning rate that was associated with making an active choice, as none of our tasks involved observing choices only.

We used a softmax rather than a sigmoid to convert learnt values to probabilities.

26 Rupprechter, Stankevicius, Huys, Steele, Seriès, *Sci Rep*. 8, 13798 (2018).

The best fitting model for this paper was a leaky beta model.

$$Q_{t+1(a_t)} = A * Q_{t(a_t)} + outcome_t$$

Where A is a memory parameter, bounded between 0 and 1, that suggests the extent to which participants remember previously obtained outcomes.

$$P_{t(a_t)} = \frac{1}{1 + exp(-\beta * (f(Q_{t(a_t)}) - \varphi))}$$

Where φ referred to the displayed reward probability, and f(x) was x/4, to make this similar to the displayed reward probabilities. As none of our benchmarking tasks had one option with a known probability and one unknown, we used a sigmoid function including the difference between the two presented options (either two stimuli, or go and no-go).

27 WHITE *ET AL.*, *AJP*. 174, 110–117 (2017).

The model fit to choice data in this paper was a standard Q learning model:

$$Q_{t+1(a_t)} = Q_{t(a_t)} + \alpha * \delta_{t(a_t)}$$

$$\delta_{t(a_t)} = outcome_t - Q_{t(a_t)}$$

With a sigmoid equation:

$$P_{t(a_t)} = \frac{exp(Q_{t(a_t)} * \beta)}{1 + exp(Q_{t(a_t)} * \beta)}$$

Note that we used a standard softmax to convert probabilities into choices in our paper.
